# Transcriptome Profiling across Five Tissues of Giant Panda

**DOI:** 10.1155/2020/3852586

**Published:** 2020-08-10

**Authors:** Feng Li, Chengdong Wang, Zhongxian Xu, Mingzhou Li, Linhua Deng, Ming Wei, Hemin Zhang, Kai Wu, Ruihong Ning, Diyan Li, Mingyao Yang, Mingwang Zhang, Qingyong Ni, Bo Zeng, Desheng Li, Ying Li

**Affiliations:** ^1^Farm Animal Genetic Resources Exploration and Innovation Key Laboratory of Sichuan Province, Sichuan Agricultural University, Chengdu 611130, China; ^2^Key Laboratory of Southwest China Wildlife Resources Conservation (Ministry of Education), China West Normal University, Nanchong 637002, China; ^3^Key Laboratory of SFGA on Conservation Biology of Rare Animals in the Giant Panda National Park (CCRCGP), Dujiangyan 611830, China

## Abstract

Gene differential expression studies can serve to explore and understand the laws and characteristics of animal life activities, and the difference in gene expression between different animal tissues has been well demonstrated and studied. However, for the world-famous rare and protected species giant panda (*Ailuropoda melanoleuca*), only the transcriptome of the blood and spleen has been reported separately. Here, in order to explore the transcriptome differences between the different tissues of the giant panda, transcriptome profiles of the heart, liver, spleen, lung, and kidney from five captive giant pandas were constructed with Illumina HiSeq 2500 platform. The comparative analysis of the intertissue gene expression patterns was carried out based on the generated RNA sequencing datasets. Analyses of Gene Ontology (GO) enrichment, Kyoto Encyclopedia of Genes and Genomes (KEGG) enrichment, and protein-protein interaction (PPI) network were performed according to the identified differentially expressed genes (DEGs). We generated 194.52 GB clean base data from twenty-five sequencing libraries and identified 18,701 genes, including 3492 novel genes. With corrected *p* value <0.05 and |log_2_FoldChange| >2, we finally obtained 921, 553, 574, 457, and 638 tissue-specific DEGs in the heart, liver, spleen, lung, and kidney, respectively. In addition, we identified TTN, CAV3, LDB3, TRDN, and ACTN2 in the heart; FGA, AHSG, and SERPINC1 in the liver; CD19, CD79B, and IL21R in the spleen; NKX2-4 and SFTPB in the lung; GC and HRG in the kidney as hub genes in the PPI network. The results of the analyses showed a similar gene expression pattern between the spleen and lung. This study provided for the first time the heart, liver, lung, and kidney's transcriptome resources of the giant panda, and it provided a valuable resource for further genetic research or other potential research.

## 1. Introduction

Gene differential expression has been demonstrated to play an important role in animal life activities, e.g., growth, development, metabolism, aging, disease, and immunity. For instance, each developmental stage of life has diverse biological features due to the regulation of differential gene expression [1, 2], and cell specifications during development become evident through differential gene expression [3]. Certain developmental gene expression pathways, including Notch, characterize the survivin gene for differential expression in transformed cells, which is related to tumorigenesis [4, 5]. Several specific pathways demonstrated age-dependent differential gene expression during aging in a cell-specific fashion. For example, genes involved in cell cycle control were upregulated in aging adipose-derived stem cells but not in aging fibroblasts [6]. Chromosome-wide and gene-specific sex differences in DNA methylation are associated with differential gene expression and metabolism [7]. Differential expression of proteins involved in metabolism, transport, and stress response is seen in the kidney from aging male mice [8].

Transcriptome differences between different tissues have been well studied so far. Rats as an extensively used animal model, the comprehensive rat RNA-Seq transcriptomic BodyMap involving 11 organs across four developmental stages from juvenile to old age for both sexes was generated. It was found that organ-enriched, differentially expressed genes (DEGs) reflect the known organ-specific biological activities, and a huge amount of transcripts showed organ-specific, sex-specific, or age-dependent differential expression patterns [9]. Similar to the rat, the comprehensive mouse transcriptomic BodyMap across 17 tissues of six-week-old mice using RNA-seq was constructed and found different expression patterns between protein-coding and noncoding genes [10]. Meanwhile, after the transcriptomes of six major tissues dissected from midgestational mouse embryos were analyzed, 1375 identified genes showed tissue-specific expression, providing gene signatures for each of the six tissues [11]. For humans, a transcriptome abundance atlas of 29 paired healthy human tissues was generated from the Human Protein Atlas project; this analysis revealed that strong mRNA differences within and across various tissues exist [12]. Transcriptome differential studies between different tissues are also widely found in domestic animals, such as transcriptome analysis of brain and liver in the Rongchang pig revealed tissue specificity through the identification of 5575 and 4600 DEGs in brains and livers, respectively [13]. Furthermore, a multiple tissue transcriptome analysis identified feed efficiency variations in related genes and biological pathways in the growing pig [14]. In addition, similar studies have been reported in other economic animals, as an example, four tissues of Atlantic salmon were collected and analyzed the transcriptomes, and the functional profiling identified gene clusters describing the unique functions of each tissue [15].

Giant panda (*Ailuropoda melanoleuca*) is a world-wide rare and flagship species for conservation [16]. According to the latest fourth national survey on giant panda conducted by the State Forestry Administration of China, the number of giant pandas living in the wild throughout China was only 1864 at the end of 2013. Although the giant panda was downlisted from “endangered” to “vulnerable” on the International Union for the Conservation of Nature (IUCN) red list, sustained conservation efforts are still needed to counteract existing and emerging threats [17–20]. These threats include but are not limited to the highly contagious and fatal canine distemper virus (CDV) that still threatens their survival [21, 22]. In addition to being rare, the giant panda itself is a very special species, with some unique physiological characteristics. In spite of the fact that the giant panda is a species of Ursidae family [23], it mainly feeds on bamboo [24], while the gut microbiota diversity and composition of the giant panda are more similar to carnivores [25, 26], it leads to a very low bamboo digestive efficiency [27, 28], and corresponding to exceptionally low daily energy expenditure in the giant panda [29]. Furthermore, the weight of newborn giant panda cubs only 1/1000 of their mothers [30].

We hypothesized that the giant panda, like other animals, has different transcriptome expression profiles between different tissues. However, in addition to blood and spleen tissues, the transcriptome of other tissues or any differences in expression patterns across various tissues of giant panda remains unknown. The giant panda draft genome sequence was generated and assembled using next-generation sequencing technology in 2010 [31], and subsequent gene annotations and protein functions were investigated [32]. However, the integrity of the giant panda genome still needs to be improved. RNA-Seq has been used to improve the global genome assembly completeness, and the novel expressed transcripts of 12 tissues from two giant pandas were analyzed using a transcriptome reconstruction strategy that combined reference-based and *de novo* methods [33]. The first study of blood transcriptomes was performed on three giant panda samples, which were characterized and analyzed using Illumina HiSeq 2000 paired-end sequencing technology. The final transcripts were mapped to the Kyoto Encyclopedia of Genes and Genomes (KEGG) pathways; the best represented functional categories were signal transduction and immune systems [34]. miRNA profiles have also been reported for four giant pandas, and the KEGG enrichment analysis revealed that the genes were mainly involved in host immunity [35]. In recent studies, the whole transcriptomes of mRNA, lncRNA, miRNA, and circRNA in the spleens of two giant pandas were sequenced using the Illumina HiSeq platform; this is also the first report of lncRNAs and circRNAs in the giant panda [36]. RNA-Seq was also performed on old and young pandas, and a comparison of transcriptomes revealed 210 DEGs; this led to the identification of immune-related genes that changed with age and identified ISG15, STAT1, IRF7, and DDX58 as the hub genes in the protein-protein interaction (PPI) network in response to pathogen invasion [37]. These studies have provided immunogenetic information that is useful for the further study of giant panda immunity.

To explore the differences in transcriptome expression profiles between different tissues of the giant panda, in the present work, using Illumina HiSeq 2500 platform, we identified and characterized large-scale transcriptome from heart, liver, spleen, lung, and kidney of five giant pandas. Furthermore, we conducted comparative analyses of DEGs, Gene Ontology (GO) enrichment, KEGG enrichment, and PPI network on these tissues. The data from this study may provide a valuable genetic resource for further bioinformatics research or other potential research.

## 2. Materials and Methods

### 2.1. Ethics Statement and Sample Preparation

Animal care, samples collection and experiments were conducted according to the guidelines established by the Regulations on the Administration of Laboratory Animals (State Science and Technology Commission of the People's Republic of China, 2017) and were approved by the Committee for the Ethics on Animal Care and Experiments at Sichuan Agricultural University under permission number DKY-B20171911-1.

Five giant pandas (four females and one male) that died by accident were used in this study (Table S1). Samples were obtained from the China Conservation and Research Center for Giant Panda in Dujiangyan based, Sichuan, China. Heart, liver, spleen, lung, and kidney samples were collected from each panda and immediately stored in liquid nitrogen. Samples were then stored at −80°C until RNA extraction.

Total RNA was extracted using an RNeasy Mini Kit (Qiagen, Hilden, Germany) according to the manufacturer protocol. RNA degradation and contamination were monitored using 1% agarose gels. A NanoPhotometer® spectrophotometer (IMPLEN, CA, USA) was used to check the purity of RNA, and a Qubit® RNA Assay Kit was used on samples, which were measured using a Qubit® 2.0 Flurometer (Life Technologies, CA, USA) to measure the concentration of RNA. RNA integrity was assessed using an RNA Nano 6000 Assay Kit for the Bioanalyzer 2100 system (Agilent Technologies, CA, USA).

### 2.2. RNA Library Construction and Illumina Sequencing

A total amount of 1 *μ*g RNA per sample was used as input material for sequencing. Twenty-five sequencing libraries (5 tissues ×5 biological repetitions per tissue) were generated using a NEBNext® Ultra™ RNA Library Prep Kit for Illumina® (NEB, USA) following manufacturer's recommendations; index codes were added to the attribute sequences of each sample [38]. Index-coded samples were then clustered on a cBot Cluster Generation System using a TruSeq PE Cluster Kit v3-cBot-HS (Illumina) in accordance with the manufacturer's instructions. After cluster generation, the library preparations were sequenced on an Illumina Hiseq 2500 platform and 150 bp paired-end reads were generated.

To ensure the quality of downstream information analysis, the raw data (raw sequence reads) files in FASTQ format obtained by sequencing were used for quality control processing. The steps for raw data processing were as follows: (1) removing sequence reads with adapter. The RNA-seq adapter information can be found in the Oligonucleotide sequences for TruSeq™ RNA and DNA Sample Prep Kits manufacturer's instructions, including RNA 5′ Adapter (RA5), part #15013205: 5′-AATGATACGGCGACCACCGAGATCTACACTCTTTCCCTACACGACGCTCTTCCGATCT-3′, RNA 3′ Adapter (RA3), part #15013207: 5′-GATCGGAAGAGCACACGTCTGAACTCCAGTCACATCTCGTATGCCGTCTTCTGCTTG-3′. (2) Removing sequence reads that ploy-N sequences with an uncertain base ratio of more than 10%. (3) Removing sequence reads with low-quality reads (reads with Qphred <20 bases accounting for more than 50% of total read length). The Qphred value and error rate of the clean data were calculated at the same time. All downstream analyses were based on the filtered high quality and clean data.

### 2.3. Gene Alignment and Expression Level Quantification

The giant panda reference genome ailMel1 (GCA_000004335.1) and gene model annotation files were downloaded from http://ensembl.org/Ailuropoda_melanoleuca/Info/Index. An index of the reference genome was built, and paired-end clean reads were aligned to the reference genome using HISAT2 v2.0.4 software with default parameters [39]. HISAT2 algorithm was mainly divided into three parts: (1) alignment of the whole sequencing sequences to the single exons of the reference genome, (2) alignment of the segmented sequences to two exons of the reference genome, and (3) alignment of the segmented sequences to three or more exons of the reference genome. The Cufflinks v2.1.1 with default parameters and reference annotation based transcript assembly method was used to construct and identify both known and novel transcripts from TopHat alignment results [40]. The genomic mapping result of all the sequenced reads data was pooled together and assembled using Cufflinks v2.1.1, and then compared with the giant panda reference genome using Cuffcompare (http://cole-trapnell-lab.github.io/cufflinks/cuffcompare/index.html#transfrag-class-codes).

Read numbers were counted and mapped to each gene using HTSeq v0.9.1 with union mode and default parameters. The fragments per kilobase of exon per million mapped fragments (FPKM) value of each gene was then calculated based on the gene length and read count mapped to the gene [41]. If the FPKM value of a gene is greater than 1, the gene is thought to be expressed in the tissue. The gene expression levels of different tissues were compared by the FPKM distribution diagram (violin plot) of all genes. For repeated samples of the same tissue, the final FPKM value of the tissue was the average of all five repeated FPKM values. To verify the credibility and repeatability of this experiment, principal component analysis (PCA) and Pearson correlation analyses of the correlation of gene expression in all samples were performed.

### 2.4. Differential Expression Analyses of each Tissue

To screen for genes that were differentially expressed between tissues, differential expression analysis of each two tissues among the heart, liver, spleen, lung, and kidney was performed using the DESeq v1.18.0 package in R software [42]. DESeq v1.18.0 provides statistical algorithms for determining differential expression from gene expression data using a model based on a negative binomial distribution. The resulting *p* values were adjusted using Benjamini and Hochberg's approach for controlling the false discovery rate (FDR) [43]. Genes with a corrected *p* value <0.05 and |log2FoldChange| >2 found by DESeq v1.18.0 were assigned as DEGs.

### 2.5. GO and KEGG Enrichment Analyses of DEGs

The analyses of DEGs' GO enrichment and KEGG enrichment were carried out to evaluate the biological role and significance of DEGs. GO enrichment analysis of DEGs was implemented using the GOseq package (Release2.12 version) in R software; this method was based on Wallenius noncentral hypergeometric distribution, in which gene length bias was corrected [44]. KOBAS v2.0 software was used to test the statistical enrichment of DEGs against the KEGG pathway database; the statistical test method was the hypergeometric test/Fisher's exact test, and the FDR correction method was Benjamini-Hochberg [45]. GO terms and KEGG pathways with a corrected *p* value <0.05 were considered significantly enriched by DEGs.

### 2.6. Protein-Protein Interaction Analyses of DEGs

To find the hub genes that play a key role in specific tissue biology functions from tissue-specific DEGs, we mainly used the STRING protein interaction database (https://string-db.org/) to analyze the DEGs' PPI network. As giant panda is the species included in the STRING database, interactions of DEGs lists were extracted directly from the database to construct the networks. Then, the network files were imported directly into Cytoscape 3.7.0 software for visual editing. Finally, the identified genes were placed on UniProt (https://www.uniprot.org/) to check out their biological function information.

## 3. Results

### 3.1. Overview of the RNA Sequencing Profiles of Giant Panda

To explore the differences in gene expression patterns between different tissues of the giant panda, as well as genes that play a key role in the specific biological functions of different tissues, in the present study, 25 sequencing libraries (5 tissues ×5 biological repetitions per tissue) were constructed from five giant pandas (Table S2). This study generated 53.63 million raw reads per sample after sequenced with Illumina Hiseq 2500 platform, and 51.87 million clean reads per sample remained after quality control. Finally, 7.78 GB clean bases per sample were generated in total for this study. All the error rates of per base sequencing were lower than 0.02%, and the Phred score Q30 value of all reads was greater than 91.83%. An average of 47.62 million clean reads per sample were successfully mapped to the giant panda Ensembl reference genome, and approximately, 46.71 million reads per sample were uniquely mapped among the total mapped reads (Table S2).

**Figure 1:**
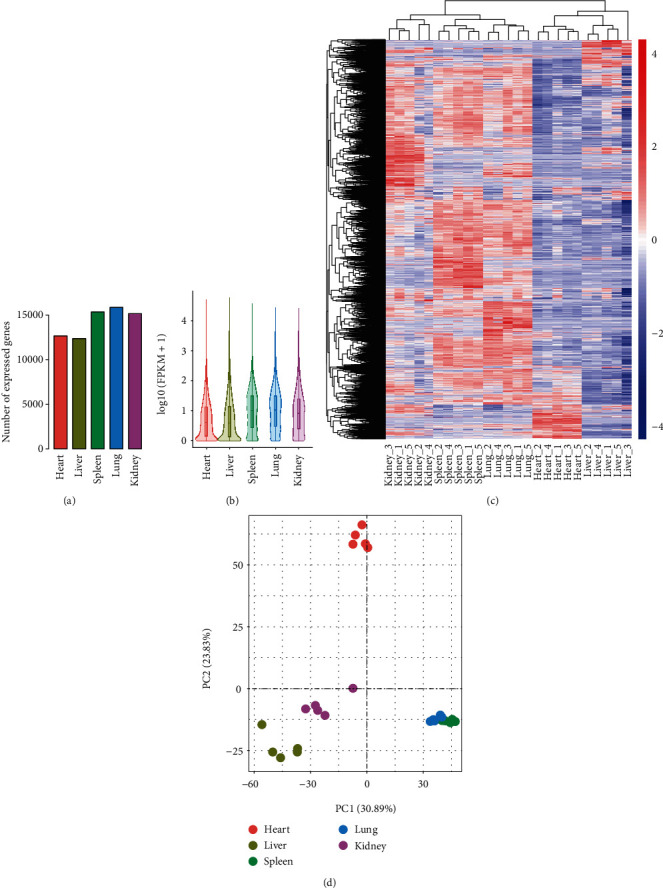
Overview of all genes expressed level in the giant panda. (a) Statistical histogram of the number of genes expressed in each tissue using the R language function barplot. (b) Violin plot generated by the ggplot2 R package based on FPKM value of all genes in each tissue. The ordinate represents log_10_(FPKM+1). Each region of violin plot corresponds to five statistics (top-down, maximum, upper quartile, median, lower quartile, and minimum). The width of each violin represents the number of genes under that expression. (c) Hierarchical clustering heatmap of all expressed genes based on the normalized FPKM values. It generated by the pheatmap R package and the red-blue spectrum represents the normalized FPKM values. (d) Principal component analysis based on the FPKM value of all expressed genes using the ggplot2 R package.

A total of 25376 genes were successfully mapped to the giant panda reference genome, which consisted of 19343 known genes (including 13 mitochondrial genes) and 6033 novel genes (Table S3). Based on the universal standard of FPKM >1, under which a gene is expressed in the tissue, a total of 18,701 genes were identified which were expressed in at least one of the five tissues. These genes were comprised of 3,492 novel genes and 15,209 known annotated genes identified in the giant panda reference genome. There were 12,657, 12,355, 15,346, 15,858, and 15,173 genes expressed in the heart, liver, spleen, lung, and kidney tissues, respectively (Figure 1(a)). The expression levels of all genes in each tissue were assessed. Overall, among the five tissues, the expression of the spleen and lung were higher than the other three (Figure 1(b)). Furthermore, in the hierarchical cluster analysis of all genes' expression levels, overall, the cluster which contained kidney, spleen, and lung had a relatively higher gene expression level (Figure 1(c)).

In the results of PCA based on the gene expression level, the five biological repetitions of each tissue in this study gathered well, while the biological repetitions of lung and spleen overlapped (Figure 1(d)). These results, together with the five biological repetitions of each tissue, were gathered into a cluster in the hierarchical cluster analysis (Figure 1(c)), indicating that the samples in this study had a good repeatability; the resulting data was believed to be credible, and also suggested that there may be a closer correlation of gene expression patterns between the spleen and lung tissues.

### 3.2. The Identified DEGs Showed Tissue Specificity

**Figure 2:**
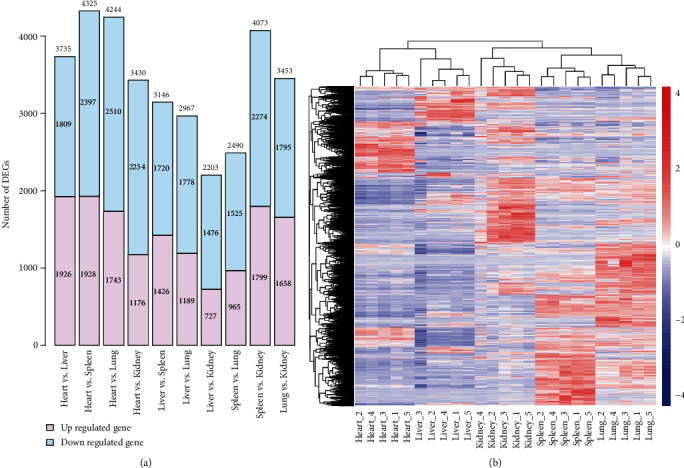
Statistics of differentially expressed genes between different tissues. (a) Histogram of differentially expressed genes number statistics between different tissues with corrected *p* value <0.05 and |log_2_FoldChange| >2. It generated by the R language function barplot. (b) Hierarchical clustering heatmap of all differentially expressed genes based on the normalized FPKM values generated by the pheatmap R package and the red-blue spectrum represents the normalized FPKM values.

Under the conditions of corrected *p* value <0.05 and |log_2_FoldChange| >2, DEGs between each two different tissues were identified (Figure 2(a)). In all tissue comparisons, the heart vs. spleen group had the largest number of DEGs (4,325, consisting of 1,928 up- and 2,397 downregulated genes), whereas the number of DEGs between liver and kidney was the least (2,203, consisting of 727 up- and 1,476 downregulated genes). The hierarchical cluster analysis of all obtained DEGs shown tissue-specific gene expressed patterns, and the five biological repetitions of each tissue were well assembled into one cluster (Figure 2(b)). Similarly, there was a closer relationship between the two clusters of the spleen and lung than other tissues.

**Figure 3:**
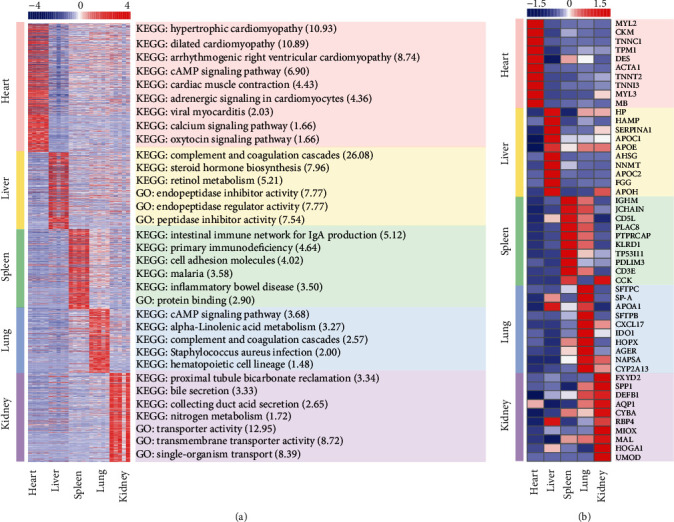
Statistics of tissue-specific differentially expressed genes between different tissues. (a) The heatmap of all tissue-specific differentially expressed genes of each tissue (left) and part of significantly enriched GO terms and KEGG pathways (right, with corrected *p* value <0.05, number in the right parentheses represents −log_10_(corrected *p* value)). (b) The heatmap of the top 10 highly expressed tissue-specific differentially expressed genes of each tissue. It generated by the pheatmap R package, and the red-blue spectrum represents the normalized FPKM values.

Based on the DEGs identified by comparison between every two tissues, we analyzed the tissue-specific DEGs in each tissue. Finally, there were 921, 553, 574, 457, and 638 tissue-specific DEGs obtained in the heart, liver, spleen, lung, and kidney, respectively (Figure 3, Figure S1, Table S4). All the tissue-specific DEGs also showed a higher gene expression level based on FPKM values (Figure 3(a)). As we expected, those tissue-specific DEGs, generally, were highly related to certain structural components or biological functions of a specific tissue (Figure 3(b), Table S4). For example, the heart-specific DEGs, like myosin light chain 2 (MYL2), were closely related to heart development and disease resistance functions [46]. The liver-specific DEGs, such as apolipoprotein C-I (APOC1), had a close relationship with the liver's energy and metabolic function [47]. As an important immune organ, most spleen-specific DEGs were related to immune and disease resistance functions, such as immunoglobulin heavy constant mu (IGHM) [48]. The lung-specific DEGs, including surfactant protein C (SFTPC), were not only directly related to respiratory function but also to disease prevention [49]. For the kidney, the tissue-specific DEGs were mainly associated with the kidney function substance reabsorption and urine formation, including the FXYD domain-containing ion transport regulator 2 (FXYD2) [50]. In addition, some tissue-specific DEGs of one tissue may also be highly expressed in another tissue at the same time, such as the gene IGHM highly expressed in spleen and lung tissues. This is probably because both tissues were closely related to immune function.

### 3.3. DEGs Significantly Enriched to Tissue-Specific GO Terms and KEGG Pathways

Using the above tissue-specific DEGs, the GO enrichment and KEGG enrichment analyses were performed separately. Overall, with corrected *p* value <0.05, tissue-specific DEGs significantly enriched to 24, 1, and 26 GO terms in the liver, spleen, and kidney, respectively (Table S5), and none of GO term was significantly enriched in the heart and lung tissues. Additionally, it significantly enriched to 9, 23, 22, 6, and 11 KEGG pathways in the heart, liver, spleen, and kidney tissues (Table S6). As expected, GO terms or KEGG pathways of tissue-specific DEGs enriched were related to tissue-specific activities (Figure 3(a), Table S5~S6), such as in the heart tissue, the KEGG pathway hypertrophic cardiomyopathy (aml05410) was significantly enriched to those heart related disease, while pathways such as cardiac muscle contraction (aml04260), were closely related to the maintenance of the heart's own essential functions. For the liver tissue, either the significantly enriched KEGG pathways steroid hormone biosynthesis (aml00140) or the significantly enriched GO terms endopeptidase regulator activity (GO: 0061135) was associated with the biological function of the liver as a major energy metabolic tissue. For the spleen tissue, the mainly significantly enriched KEGG pathways and GO term, including intestinal immune network for IgA production (aml04672), were closely related to immunity or disease. The most significantly enriched KEGG pathways in lung tissue included cAMP signaling pathway (aml04024) and complement and coagulation cascades (aml04610); those pathways had a close relationship with lung functions. For example, metabolism and immune function and the tissue-specific DEGs also significantly enriched to the KEGG pathway hematopoietic cell lineage (aml04640). For the last tissue, the kidney, significantly enriched KEGG pathways like nitrogen metabolism (aml00910), or GO terms like transporter activity (GO: 0005215), all were closely related to the secretion and reabsorption functions of the kidney.

### 3.4. Hub Genes of each Tissue Obtained from PPI Analysis of DEGs

**Figure 4:**
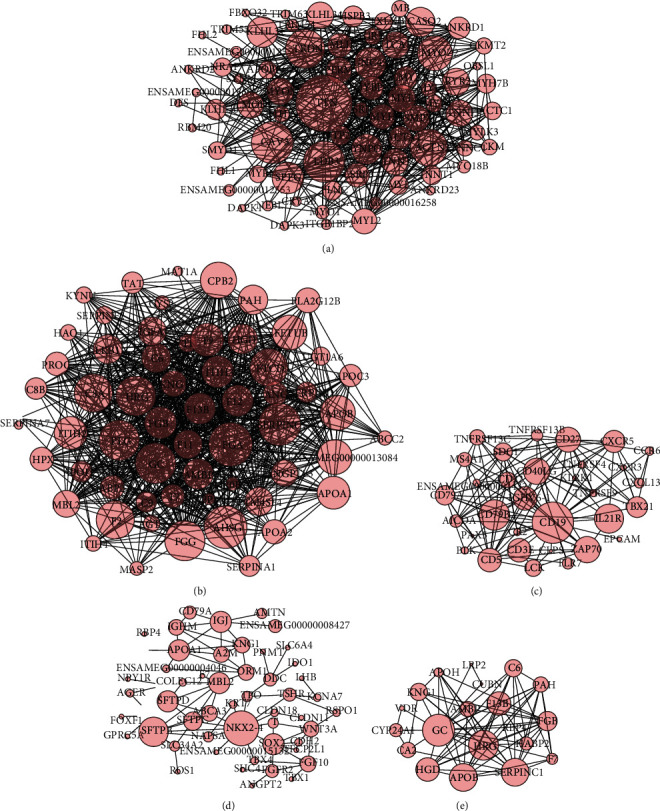
Protein-protein interaction networks of the heart (a), liver (b), spleen (c), lung (e), and kidney (e). The size of each node in the protein-protein interaction networks presents the connect degree of each gene. Those nodes that were not connected to any node were omitted in the network. The networks were first generated in the STRING database and then visually edited in Cytoscape software.

The tissue-specific DEGs obtained from previous differential expression analysis were placed in the STRING database for PPI analysis. And then, after network analysis and visualization in Cytoscape software, we obtained the PPI network of all DEGs and hub genes. All the hub genes of each tissue were at key positions in the interaction networks (Figure 4). The genes including titin (TTN), caveolin 3 (CAV3), LIM domain binding 3 (LDB3), triadin (TRDN), and actinin alpha 2 (ACTN2) in the heart; fibrinogen alpha chain (FGA), alpha 2-HS glycoprotein (AHSG), and serpin family C member 1 (SERPINC1) in the liver; CD19 molecule (CD19), CD79b molecule (CD79B), and interleukin 21 receptor (IL21R) in the spleen; NK2 homeobox 4 (NKX2-4) and surfactant protein B (SFTPB) in the lung; GC vitamin D binding protein (GC) and histidine-rich glycoprotein (HRG) in the kidney play a key role in tissue-specific functions, respectively.

## 4. Discussion

Previous studies have shown that there are significant gene expression differences between different tissues, include studies on humans [51], pigs [52], mice [10], and rats [9]. Although the heart, liver, spleen, lung, and kidney are vital tissues for the giant panda, transcriptome analyses have only been reported for the blood and spleen [34–37]. Thus, this is the first time that the transcriptomes of the giant panda heart, liver, lung, and kidney tissues have been reported. It is also the first comparative transcriptome analysis for the heart, liver, spleen, lung, and kidney of the giant panda.

In the previous studies, a total of 160 million clean reads and 13.61 GB clean bases were generated from three giant panda blood samples; in addition, two spleens from one newborn and one adult giant panda yielded 36.58 million and 52.05 million clean reads, respectively [36]. In the present study, an average of 51.87 million clean reads per sample and 7.78 GB clean base per sample were obtained (Table S2); this output was equivalent or superior to the reported sequencing results in the giant panda [34, 36]. Furthermore, whether in the hierarchical cluster analysis of all genes (Figure 1(c)) or in the PCA of all genes (Figure 1(d)), the five biological repetitions of each tissue were well grouped together or highly relevant, indicating that the RNA-Seq results obtained in this study had high credibility and repeatability, which ensured that the follow-up analyses were based on an operational and meaningful basis.

Overall, the results of the differential expression analysis of each tissue showed the unique gene expression patterns. However, two tissues, the spleen and lung were highly similar in gene expression patterns. In terms of all gene expression statistics based on FPKM values, both the spleen and lung had relatively high and similar distributions of expression levels (Figures 1(b) and 1(c)) and were simultaneously clustered on a small branch in a hierarchical clustering analysis of all genes (Figure 1(c)) and all DEGs (Figure 2(b)). The gene expression level correlation revealed that the biological replicate samples for spleen and lung tissues were clustered in the PCA graph (Figure 1(d)). Among the results of differential expression gene analysis in all five tissues, the number of DEGs between the spleen and lung was one of the smallest, only 2,490 (Figure 2(a)), and some of the tissue-specific DEGs in the spleen, such as IGHM, JCHAIN, were also highly expressed in the lung (Figure 3(b)). Furthermore, in KEGG enrichment results, DEGs in the spleen and lung were enriched in the same KEGG pathways, such as hematopoietic cell lineage (aml04640) (Table S6). Our results suggest that the spleen and lung tissues may share very close biological similarity or may be closely related to the same immune responses, which is consistent with previous studies, such as coparticipation in heavy metal cadmium-induced poisoning [53] or nonlethal systemic *Aspergillus fumigatus* infection [54].

The hub genes obtained from the PPI network analyses were closely related to the unique structure or function of the tissue (Figure 4). In those hub genes identified in PPI analysis, the protein Titin encoded by the gene TTN is a key component in the assembly and functioning of vertebrate striated muscles, and a mutation in the TTN gene can cause familial dilated cardiomyopathy (DCM) [55], centronuclear myopathy [56] and is involved in progressive muscular dystrophies during early human development [57]. The TTN originated stiffness decrease will lead to systolic dysfunction in DCM heart muscles [58, 59]. By influencing the elasticity of myocardial muscle, TTN associated with preserved ejection fraction with heart failure [60, 61]. CAV3 may act as a scaffolding protein within caveolar membranes, interacting directly with G-protein alpha subunits and can functionally regulate their activity (https://www.uniprot.org/), which can regulate caveolae function [62]. LDB3 gene mutations cause idiopathic dilated cardiomyopathy (IDCM) [63] and sometimes occur simultaneously with both prominent left ventricular trabeculation and congenital left ventricular aneurysms [64]. TRDN contributes to the regulation of lumenal Ca2+ release via the sarcoplasmic reticulum calcium release channels RYR1 and RYR2, a key step in triggering skeletal and heart muscle contraction, and TRDN play a role in excitation-contraction coupling in the heart and in regulating the rate of heartbeats (https://www.uniprot.org/). TRDN is required for the triplet connection of normal tissues, where the T-tube and sarcoplasmic reticulum terminal cisternae are in close contact and are required for normal skeletal muscle strength [65–68]. ACTN2 is linked with heart failure [69], especially its mutation that can cause hypertrophic cardiomyopathy [70, 71]. FGA first produces monomers from protease thrombin cleavage and then polymerizes with fibrinogen beta (FGB) and fibrinogen gamma (FGG) to form an insoluble fibrin matrix, and fibrin plays an important role in hemostasis as one of the main components of blood clots [72]. The mutation of FGA will cause the clinical disorder of amyloidosis in the liver or other tissues [73]. Although AHSG is mainly expressed in the liver, it can affect type2 diabetes and tumor progression [74]. SERPINC1 is the most important serine protease inhibitor in plasma that regulates the blood coagulation cascade [75]. CD19 serves as a coreceptor for the B-cell antigen receptor complex (BCR) on B-lymphocytes and can reduce the threshold for activating the downstream signaling pathway and triggering the B-cell antigen response. It is required for normal levels of serum immunoglobulin and to produce high-affinity antibodies in response to antigen challenge [76, 77]. Heterodimer composed of CD79b and CD79a is an important signaling component of the B-cell receptor complex and plays a crucial role in the B-cell development and antibody production [78]. IL21R signaling plays a significant role in promoting follicular helper T (Tfh) cell-mediated cardiac injury in viral myocarditis (VMC) [79] and response to chronic allograft nephropathy (CAN) [80]. NKX2-4 may be related to DNA-binding transcription factor activity and cell differentiation [81]. SFTPB promotes alveolar stability by lowering the surface tension at the air-liquid interface in the peripheral air spaces [81, 82]. GC is involved in vitamin D transport and storage, scavenging of extracellular G-actin, enhancement of the chemotactic activity of C5 alpha for neutrophils in inflammation, and macrophage activation [83]. HRG plays an important role in heavy metal ion transport, such as cadmium adsorption [84].

Though studies have shown differences in transcriptome patterns among animals of different ages [37, 85, 86], physiological conditions [87] or sex [86, 88], studying differences of different ages, conditions, or genders requires a certain amount of sample size to make the corresponding results meaningful. For instance, Yu et al. used at least 320 rat samples to study transcriptome expression patterns between 11 different organs, two genders, and four different developmental stages [9]. As the giant panda is the national treasure of China and is a world-famous rare and conserved animal, the method of obtaining the sample involved in this study is special; we cannot take the method of euthanasia to collect the sample like is used in collecting samples from rats or other animals, and the corresponding sample can only be obtained in the case of accidental death of the giant panda. Our samples collected from five individual giant pandas, consisting of four females and one male, or three young (death from virus infection), one adult (death from ileac pssion), and one aged (death from ovarian cancer) (Table S1). As the sample size is limited, the study of transcriptome differences between different ages, conditions, or genders is greatly affected by the individual and quantity and has little significance. Furthermore, the results of this study, such as the five biological repeats of each tissue in the heatmap and PCA of all tissue samples were gathered together, while the five tissues that were separated from each other (Figures 1(c) and 1(d)) have shown that the differences between the biological repetitions within tissues, compared with the differences between tissues, appear to be very small, and are not enough to affect the differences between tissues. Moreover, in the previous transcriptome study on the blood and spleen of giant panda [34, 36, 37], the number of giant pandas used was only four at most, so we believe that the use of five giant pandas as biological repetitions of each tissue sample in this study is reference-based and has certain biological significance.

Although, the samples used in this study were all from captive giant pandas, and studies have shown that there are differences in physiological characteristics between captive and wild giant pandas, such as changes in the gut microbiome, where fecal microbiome diversity was significantly lower in captive giant panda, as was the diversity of functional genes [89]. We think this difference may be due to the different diets between captive and wild giant pandas. The main diet for captive giant pandas is bamboo, fruit, carrot, steamed grain mixture, and some animal products [90], while almost 99% of the diet for wild giant panda is bamboo [91]. Therefore, we speculate that the tissue transcriptome pattern of the wild giant panda may be no different from that of the captive giant panda.

## 5. Conclusions

In the present study, we first reported the transcriptome of four tissues (heart, liver, lung, and kidney) of the giant panda, we also found differences in gene expression patterns among the five tissues (add spleen). This dataset provided a valuable resource for further research on the genetics and immune/disease of the giant panda.

## Data Availability

The datasets generated for this study can be found in the Gene Expression Omnibus (GEO) repository at the National Center for Biotechnology Information (NCBI) with accession number GSE138294.

## Supplementary Materials

Supplementary Materials is available for this paper at https://new.hindawi.com/journals/bmri/. Supplementary Table S1: individual information of the giant panda used in this study. Supplementary Table S2: quality summary of RNA-seq results in all samples of the giant panda. Supplementary Table S3: details of all genes mapped to the giant panda reference genome in the present study. Supplementary Table S4: list of tissue-specific differentially expressed genes of each tissue of giant panda. Supplementary Table S5: list of significantly enriched GO terms of tissue-specific differentially expressed genes of each tissue. Supplementary Table S6: list of significantly enriched KEGG pathways of tissue-specific differentially expressed genes of each tissue. Supplementary Figure S1: four-way Venn diagram of tissue-specific differentially expressed 466 genes of each tissue.
