# Corrigendum to “Transcriptome Profiling across Five Tissues of Giant Panda”

**DOI:** 10.1155/2022/9846545

**Published:** 2022-05-19

**Authors:** Feng Li, Chengdong Wang, Zhongxian Xu, Mingzhou Li, Linhua Deng, Ming Wei, Hemin Zhang, Kai Wu, Ruihong Ning, Diyan Li, Mingyao Yang, Mingwang Zhang, Qingyong Ni, Bo Zeng, Desheng Li, Ying Li

**Affiliations:** ^1^Farm Animal Genetic Resources Exploration and Innovation Key Laboratory of Sichuan Province, Sichuan Agricultural University, Chengdu 611130, China; ^2^Key Laboratory of Southwest China Wildlife Resources Conservation (Ministry of Education), China West Normal University, Nanchong 637002, China; ^3^Key Laboratory of SFGA on Conservation Biology of Rare Animals in the Giant Panda National Park (CCRCGP), Dujiangyan 611830, China

Following the publication of the article titled “Transcriptome Profiling across Five Tissues of Giant Panda” [1], the authors identified that the published Supplementary Material is not correct, and the correct Supplementary Materials are provided as follows.

## Supplementary Materials

Supplementary 1 Table S1: individual information of the giant panda used in this study.

Supplementary 2 Table S2: quality summary of RNA-seq results in all samples of the giant panda.

Supplementary 3 Table S3: details of all genes mapped to the giant panda reference genome in the present study.

Supplementary 4 Table S4: list of tissue-specific differentially expressed genes of each tissue of giant panda.

Supplementary 5 Table S5: list of significantly enriched GO terms of tissue-specific differentially expressed genes of each tissue.

Supplementary 6 Table S6: list of significantly enriched KEGG pathways of tissue-specific differentially expressed genes of each tissue.

Supplementary 7 Figure S1: four-way Venn diagram of tissue-specific differentially expressed genes of each tissue (Supplementary Materials).

## References

[1] Li F., Wang C., Xu Z. (2020). Transcriptome Profiling across Five Tissues of Giant Panda. *BioMed Research International*.

