# Supplementary material for: Transcriptome Profiling across Five Tissues of Giant Panda
Source: Biomed Res Int. 2020 Aug 10;2020:3852586. doi: 10.1155/2020/3852586 (PMC7436357; doi:10.1155/2020/3852586)
Supplement: Supplementary Materials — is available for this paper at https://new.hindawi.com/journals/bmri/. Supplementary Table S1: individual information of the giant panda used in this study. Supplementary Table S2: quality summary of RNA-seq results in all samples of the giant panda. Supplementary Table S3: details of all genes mapped to the giant panda reference genome in the present study. Supplementary Table S4: list of tissue-specific differentially expressed genes of each tissue of giant panda. Supplementary Table S5: list of significantly enriched GO terms of tissue-specific differentially expressed genes of each tissue. Supplementary Table S6: list of significantly enriched KEGG pathways of tissue-specific differentially expressed genes of each tissue. Supplementary Figure S1: four-way Venn diagram of tissue-specific differentially expressed 466 genes of each tissue. [file 3852586.f1.zip › Supplementary Table S5 List of significantly enriched KEGG pathways of tis.pdf]

Supplementary Information

BioMed Research International

Transcriptome profiling across five tissues of giant panda

Feng Li[1,2,4], Chengdong Wang[3,4], Zhongxian Xu[1,4], Mingzhou Li[1], Linhua Deng[3], Ming Wei[3], Hemin Zhang[3], Kai Wu[3], Ruihong Ning[1], Diyan Li[1], Mingyao Yang[1], Mingwang Zhang[1], Qingyong Ni[1], Bo Zeng[1*], Desheng Li[3*] and Ying Li[1*]

[1] Farm Animal Genetic Resources Exploration and Innovation Key Laboratory of Sichuan Province, Sichuan Agricultural University, Chengdu 611130, China.

[2] Key Laboratory of Southwest China Wildlife Resources Conservation (Ministry of Education), China West Normal University, Nanchong 637002, China.

[3] Key Laboratory of SFGA on Conservation Biology of Rare Animals in the Giant Panda National Park (CCRCGP), Dujiangyan 611830, China.

[4] These authors contributed equally to this work.

[*] Correspondence should be addressed to Ying Li, yingli@sicau.edu.cn; Desheng Li, 1050133153@qq.com; Bo Zeng, apollobovey@163.com.

Supplementary Table S5: List of significantly enriched KEGG pathways of tissue-specific differential expressed genes of each tissue.

Tissue	KEGG ID	Pathway	Input number	Background number	-log10 (Corrected p-Value)
Heart	aml05410	Hypertrophic cardiomyopathy (HCM)	29	78	10.93
Heart	aml05414	Dilated cardiomyopathy (DCM)	29	81	10.89
Heart	aml05412	Arrhythmogenic right ventricular cardiomyopathy (ARVC)	24	69	8.74
Heart	aml04024	cAMP signaling pathway	35	187	6.90
Heart	aml04260	Cardiac muscle contraction	18	75	4.43
Heart	aml04261	Adrenergic signaling in cardiomyocytes	25	142	4.36
Heart	aml05416	Viral myocarditis	11	52	2.03
Heart	aml04921	Oxytocin signaling pathway	19	148	1.66
Heart	aml04020	Calcium signaling pathway	21	173	1.66
Kidney	aml04964	Proximal tubule bicarbonate reclamation	7	22	26.08
Kidney	aml04976	Bile secretion	10	64	7.96

Kidney	aml04610	Complement and coagulation cascades	9	63	5.74
Kidney	aml04971	Gastric acid secretion	9	70	5.21
Kidney	aml04966	Collecting duct acid secretion	6	26	3.82
Kidney	aml04961	Endocrine and other factor-regulated calcium reabsorption	7	42	3.70
Kidney	aml00460	Cyanoamino acid metabolism	3	5	3.70
Kidney	aml00910	Nitrogen metabolism	4	16	3.70
Kidney	aml04977	Vitamin digestion and absorption	4	20	3.60
Kidney	aml00330	Arginine and proline metabolism	6	56	3.32
Kidney	aml00340	Histidine metabolism	4	23	3.22
Liver	aml04610	Complement and coagulation cascades	37	63	2.92
Liver	aml00140	Steroid hormone biosynthesis	15	38	2.53
Liver	aml05204	Chemical carcinogenesis	14	52	2.25
Liver	aml00830	Retinol metabolism	12	41	2.08
Liver	aml01100	Metabolic pathways	70	1131	2.02
Liver	aml05150	Staphylococcus aureus infection	10	42	1.91
Liver	aml00591	Linoleic acid metabolism	8	24	1.91
Liver	aml00980	Metabolism of xenobiotics by cytochrome P450	10	43	1.91
Liver	aml00982	Drug metabolism - cytochrome P450	10	45	1.60
Liver	aml00500	Starch and sucrose metabolism	9	39	1.51
Liver	aml00053	Ascorbate and aldarate metabolism	6	14	1.46
Liver	aml01230	Biosynthesis of amino acids	11	70	1.44
Liver	aml00590	Arachidonic acid metabolism	9	53	5.12
Liver	aml00120	Primary bile acid biosynthesis	5	15	4.64
Liver	aml04975	Fat digestion and absorption	7	38	4.02
Liver	aml00983	Drug metabolism - other enzymes	6	28	3.58
Liver	aml02010	ABC transporters	7	42	3.50
Liver	aml00040	Pentose and glucuronate interconversions	5	20	3.31
Liver	aml00330	Arginine and proline metabolism	8	56	3.26
Liver	aml04976	Bile secretion	8	64	2.95
Liver	aml03320	PPAR signaling pathway	8	67	2.81
Liver	aml00260	Glycine, serine and threonine metabolism	6	40	2.81
Liver	aml00860	Porphyrin and chlorophyll metabolism	5	28	2.60
Lung	aml04024	cAMP signaling pathway	16	187	2.59
Lung	aml00592	alpha-Linolenic acid metabolism	6	20	2.52
Lung	aml04610	Complement and coagulation cascades	8	63	2.35
Lung	aml05150	Staphylococcus aureus infection	6	42	2.35
Lung	aml04640	Hematopoietic cell lineage	7	78	2.07

Lung	aml00591	Linoleic acid metabolism	4	24	1.79
Spleen	aml04672	Intestinal immune network for IgA production	12	40	1.70
Spleen	aml05340	Primary immunodeficiency	10	30	1.70
Spleen	aml04514	Cell adhesion molecules (CAMs)	17	124	1.53
Spleen	aml05144	Malaria	10	45	1.38
Spleen	aml05321	Inflammatory bowel disease (IBD)	11	59	1.37
Spleen	aml05330	Allograft rejection	8	30	3.68
Spleen	aml05310	Asthma	7	22	3.27
Spleen	aml04940	Type I diabetes mellitus	8	36	2.57
Spleen	aml04020	Calcium signaling pathway	17	173	2.00
Spleen	aml04640	Hematopoietic cell lineage	11	78	1.48
Spleen	aml04060	Cytokine-cytokine receptor interaction	18	201	1.34
Spleen	aml04064	NF-kappa B signaling pathway	11	85	3.34
Spleen	aml04660	T cell receptor signaling pathway	12	103	3.33
Spleen	aml05320	Autoimmune thyroid disease	7	37	2.76
Spleen	aml05332	Graft-versus-host disease	6	26	2.65
Spleen	aml05142	Chagas disease (American trypanosomiasis)	11	103	2.65
Spleen	aml04612	Antigen processing and presentation	7	49	2.55
Spleen	aml05162	Measles	11	118	1.86
Spleen	aml05416	Viral myocarditis	7	52	1.72
Spleen	aml05150	Staphylococcus aureus infection	6	42	1.46
Spleen	aml05323	Rheumatoid arthritis	8	78	1.36
Spleen	aml05166	HTLV-I infection	16	237	1.35
