# Supplementary material for: Transcriptome Profiling across Five Tissues of Giant Panda
Source: Biomed Res Int. 2020 Aug 10;2020:3852586. doi: 10.1155/2020/3852586 (PMC7436357; doi:10.1155/2020/3852586)
Supplement: Supplementary Materials — is available for this paper at https://new.hindawi.com/journals/bmri/. Supplementary Table S1: individual information of the giant panda used in this study. Supplementary Table S2: quality summary of RNA-seq results in all samples of the giant panda. Supplementary Table S3: details of all genes mapped to the giant panda reference genome in the present study. Supplementary Table S4: list of tissue-specific differentially expressed genes of each tissue of giant panda. Supplementary Table S5: list of significantly enriched GO terms of tissue-specific differentially expressed genes of each tissue. Supplementary Table S6: list of significantly enriched KEGG pathways of tissue-specific differentially expressed genes of each tissue. Supplementary Figure S1: four-way Venn diagram of tissue-specific differentially expressed 466 genes of each tissue. [file 3852586.f1.zip › Supplementary Table S4 List of significantly enriched GO terms of tissue-specifi.pdf]

Supplementary Information

BioMed Research International

Transcriptome profiling across five tissues of giant panda

Feng Li[1,2,4], Chengdong Wang[3,4], Zhongxian Xu[1,4], Mingzhou Li[1], Linhua Deng[3], Ming Wei[3], Hemin Zhang[3], Kai Wu[3], Ruihong Ning[1], Diyan Li[1], Mingyao Yang[1], Mingwang Zhang[1], Qingyong Ni[1], Bo Zeng[1*], Desheng Li[3*] and Ying Li[1*]

[1] Farm Animal Genetic Resources Exploration and Innovation Key Laboratory of Sichuan Province, Sichuan Agricultural University, Chengdu 611130, China.

[2] Key Laboratory of Southwest China Wildlife Resources Conservation (Ministry of Education), China West Normal University, Nanchong 637002, China.

[3] Key Laboratory of SFGA on Conservation Biology of Rare Animals in the Giant Panda National Park (CCRCGP), Dujiangyan 611830, China.

[4] These authors contributed equally to this work.

[*] Correspondence should be addressed to Ying Li, yingli@sicau.edu.cn; Desheng Li, 1050133153@qq.com; Bo Zeng, apollobovey@163.com.

Supplementary Table S4: List of significantly enriched GO terms of tissue-specific differential expressed genes of each tissue.

MF: molecular function, CC: cellular component, BP: biological process

Tissue	GO ID	Terms	Term type	-log10 (Corrected *p*-Value)	DEG item	DEG list
Liver	GO:0004866	endopeptidase inhibitor activity	MF	7.77	18	417
Liver	GO:0061135	endopeptidase regulator activity	MF	7.77	18	417
Liver	GO:0030414	peptidase inhibitor activity	MF	7.54	20	417
Liver	GO:0061134	peptidase regulator activity	MF	7.54	20	417
Liver	GO:0004857	enzyme inhibitor activity	MF	4.89	21	417
Liver	GO:0004252	serine-type endopeptidase activity	MF	4.25	21	417
Liver	GO:0005576	extracellular region	CC	3.75	56	417
Liver	GO:0020037	heme binding	MF	3.62	14	417
Liver	GO:0005506	iron ion binding	MF	3.43	16	417
Liver	GO:0046906	tetrapyrrole binding	MF	3.43	14	417
Liver	GO:0016705	oxidoreductase activity, acting on paired donors, with incorporation or reduction of molecular oxygen	MF	3.43	17	417
Liver	GO:0008236	serine-type peptidase activity	MF	3.36	22	417

Liver	GO:0017171	serine hydrolase activity	MF	3.36	22	417
Liver	GO:0004867	serine-type endopeptidase inhibitor activity	MF	2.99	9	417
Liver	GO:0006869	lipid transport	BP	2.71	11	417
Liver	GO:0030212	hyaluronan metabolic process	BP	2.71	4	417
Liver	GO:1903510	mucopolysaccharide metabolic process	BP	2.71	4	417
Liver	GO:0010876	lipid localization	BP	2.18	11	417
Liver	GO:0030234	enzyme regulator activity	MF	2.06	24	417
Liver	GO:0005615	extracellular space	CC	2.03	15	417
Liver	GO:0016491	oxidoreductase activity	MF	1.84	49	417
Liver	GO:0055114	oxidation-reduction process	BP	1.75	46	417
Liver	GO:0004869	cysteine-type endopeptidase inhibitor activity	MF	1.36	4	417
Liver	GO:0004175	endopeptidase activity	MF	1.34	30	417
Spleen	GO:0005515	protein binding	MF	2.90	167	395
Kidney	GO:0005215	transporter activity	MF	12.95	91	431
Kidney	GO:0022857	transmembrane transporter activity	MF	8.72	74	431
Kidney	GO:0044765	single-organism transport	BP	8.39	95	431
Kidney	GO:1902578	single-organism localization	BP	8.30	96	431
Kidney	GO:0022891	substrate-specific transmembrane transporter activity	MF	6.15	61	431
Kidney	GO:0055085	transmembrane transport	BP	6.09	52	431
Kidney	GO:0015075	ion transmembrane transporter activity	MF	6.09	59	431
Kidney	GO:0022892	substrate-specific transporter activity	MF	6.09	63	431
Kidney	GO:0051234	establishment of localization	BP	6.09	106	431
Kidney	GO:0006810	transport	BP	6.01	105	431
Kidney	GO:0006811	ion transport	BP	5.75	57	431
Kidney	GO:0051179	localization	BP	5.20	106	431
Kidney	GO:0006820	anion transport	BP	4.16	17	431
Kidney	GO:0008509	anion transmembrane transporter activity	MF	4.08	14	431
Kidney	GO:0015698	inorganic anion transport	BP	3.11	10	431
Kidney	GO:0015103	inorganic anion transmembrane transporter activity	MF	2.86	8	431
Kidney	GO:0005216	ion channel activity	MF	2.17	26	431
Kidney	GO:0022838	substrate-specific channel activity	MF	2.17	26	431
Kidney	GO:0015267	channel activity	MF	2.17	26	431
Kidney	GO:0022803	passive transmembrane transporter activity	MF	2.17	26	431
Kidney	GO:0016020	membrane	CC	2.15	172	431
Kidney	GO:0001533	cornified envelope	CC	2.01	4	431

Kidney	GO:0015321	sodium-dependent phosphate transmembrane transporter activity	MF	1.91	3	431
Kidney	GO:0044341	sodium-dependent phosphate transport	BP	1.91	3	431
Kidney	GO:0008324	cation transmembrane transporter activity	MF	1.41	35	431
Kidney	GO:0005509	calcium ion binding	MF	1.35	27	431
