# Supplementary material for: Transcriptome Profiling across Five Tissues of Giant Panda
Source: Biomed Res Int. 2020 Aug 10;2020:3852586. doi: 10.1155/2020/3852586 (PMC7436357; doi:10.1155/2020/3852586)
Supplement: Supplementary Materials — is available for this paper at https://new.hindawi.com/journals/bmri/. Supplementary Table S1: individual information of the giant panda used in this study. Supplementary Table S2: quality summary of RNA-seq results in all samples of the giant panda. Supplementary Table S3: details of all genes mapped to the giant panda reference genome in the present study. Supplementary Table S4: list of tissue-specific differentially expressed genes of each tissue of giant panda. Supplementary Table S5: list of significantly enriched GO terms of tissue-specific differentially expressed genes of each tissue. Supplementary Table S6: list of significantly enriched KEGG pathways of tissue-specific differentially expressed genes of each tissue. Supplementary Figure S1: four-way Venn diagram of tissue-specific differentially expressed 466 genes of each tissue. [file 3852586.f1.zip › Supplementary Table S3 List of tissue-specific differentiala.pdf]

Supplementary Information

BioMed Research International

Transcriptome profiling across five tissues of giant panda

Feng Li[1,2,4], Chengdong Wang[3,4], Zhongxian Xu[1,4], Mingzhou Li[1], Linhua Deng[3], Ming Wei[3], Hemin Zhang[3], Kai Wu[3], Ruihong Ning[1], Diyan Li[1], Mingyao Yang[1], Mingwang Zhang[1], Qingyong Ni[1], Bo Zeng[1*], Desheng Li[3*] and Ying Li[1*]

[1] Farm Animal Genetic Resources Exploration and Innovation Key Laboratory of Sichuan Province, Sichuan Agricultural University, Chengdu 611130, China.

[2] Key Laboratory of Southwest China Wildlife Resources Conservation (Ministry of Education), China West Normal University, Nanchong 637002, China.

[3] Key Laboratory of SFGA on Conservation Biology of Rare Animals in the Giant Panda National Park (CCRCGP), Dujiangyan 611830, China.

[4] These authors contributed equally to this work.

[*] Correspondence should be addressed to Ying Li, yingli@sicau.edu.cn; Desheng Li, 1050133153@qq.com; Bo Zeng, apollobovey@163.com.

Supplementary Table S3: List of tissue-specific differential expressed genes of each tissue of giant panda.

Tissue	Gene id	Gene name	Gene description
Heart	ENSAMEG00000000017	ILDR2	immunoglobulin like domain containing receptor 2
Heart	ENSAMEG00000000020	DUSP27	dual specificity phosphatase 27, atypical
Heart	ENSAMEG00000000028	CTH	cystathionine gamma-lyase
Heart	ENSAMEG00000000066	ACP5	acid phosphatase 5, tartrate resistant
Heart	ENSAMEG00000000091	BTBD6	BTB domain containing 6
Heart	ENSAMEG00000000101	MORN4	MORN repeat containing 4
Heart	ENSAMEG00000000108	ANKRD2	ankyrin repeat domain 2
Heart	ENSAMEG00000000136	TRPC4	transient receptor potential cation channel subfamily C member 4
Heart	ENSAMEG00000000156	SEPTIN6	septin 6
Heart	ENSAMEG00000000212	FXYD1	FXYD domain containing ion transport regulator 1
Heart	ENSAMEG00000000218	--	--
Heart	ENSAMEG00000000228	LSR	lipolysis stimulated lipoprotein receptor
Heart	ENSAMEG00000000285	HOOK1	hook microtubule tethering protein 1
Heart	ENSAMEG00000000317	--	--
Heart	ENSAMEG00000000366	G6PD	glucose-6-phosphate dehydrogenase

Heart	ENSAMEG00000000377	CCDC141	coiled-coil domain containing 141
Heart	ENSAMEG00000000394	TTN	titin
Heart	ENSAMEG00000000462	ZNF280B	zinc finger protein 280B
Heart	ENSAMEG00000000470	DTNA	dystrobrevin alpha
Heart	ENSAMEG00000000485	LRRC8B	leucine rich repeat containing 8 VRAC subunit B
Heart	ENSAMEG00000000519	OSR2	odd-skipped related transciption factor 2
Heart	ENSAMEG00000000522	EDN2	endothelin 2
Heart	ENSAMEG00000000543	TM6SF1	transmembrane 6 superfamily member 1
Heart	ENSAMEG00000000546	TECRL	trans-2,3-enoyl-CoA reductase like
Heart	ENSAMEG00000000576	EPHX4	epoxide hydrolase 4
Heart	ENSAMEG00000000582	BTBD1	BTB domain containing 1
Heart	ENSAMEG00000000610	LARGE2	LARGE xylosyl- and glucuronyltransferase 2
Heart	ENSAMEG00000000624	TULP1	tubby like protein 1
Heart	ENSAMEG00000000640	HSPB6	heat shock protein family B (small) member 6
Heart	ENSAMEG00000000671	PLPPR1	phospholipid phosphatase related 1
Heart	ENSAMEG00000000710	--	--
Heart	ENSAMEG00000000711	CAP2	cyclase associated actin cytoskeleton regulatory protein 2
Heart	ENSAMEG00000000763	TP63	tumor protein p63
Heart	ENSAMEG00000000775	DMPK	DM1 protein kinase
Heart	ENSAMEG00000000776	KIAA1324L	KIAA1324 like
Heart	ENSAMEG00000000866	MYLK3	myosin light chain kinase 3
Heart	ENSAMEG00000000923	ABCB4	ATP binding cassette subfamily B member 4
Heart	ENSAMEG00000000931	FHOD3	formin homology 2 domain containing 3
Heart	ENSAMEG00000000988	PACSIN1	protein kinase C and casein kinase substrate in neurons 1
Heart	ENSAMEG00000001051	COX7A1	cytochrome c oxidase subunit 7A1
Heart	ENSAMEG00000001117	ACTN2	actinin alpha 2
Heart	ENSAMEG00000001161	ST3GAL3	ST3 beta-galactoside alpha-2,3-sialyltransferase 3
Heart	ENSAMEG00000001296	XIRP2	xin actin binding repeat containing 2
Heart	ENSAMEG00000001314	MET	MET proto-oncogene, receptor tyrosine kinase
Heart	ENSAMEG00000001332	DNAJB6	DnaJ heat shock protein family (Hsp40) member B6
Heart	ENSAMEG00000001404	MB	myoglobin
Heart	ENSAMEG00000001424	ADAMTSL4	ADAMTS like 4
Heart	ENSAMEG00000001435	NUAK2	NUAK family kinase 2
Heart	ENSAMEG00000001476	CDK18	cyclin dependent kinase 18
Heart	ENSAMEG00000001550	PTP4A3	protein tyrosine phosphatase type IVA, member 3
Heart	ENSAMEG00000001552	EEF1A2	eukaryotic translation elongation factor 1 alpha 2
Heart	ENSAMEG00000001578	ADAMDEC1	ADAM like decysin 1
Heart	ENSAMEG00000001616	UCN	urocortin
Heart	ENSAMEG00000001625	TRIM54	tripartite motif containing 54
Heart	ENSAMEG00000001638	IQGAP2	IQ motif containing GTPase activating protein 2
Heart	ENSAMEG00000001662	TBX20	T-box 20
Heart	ENSAMEG00000001730	DUSP13	dual specificity phosphatase 13
Heart	ENSAMEG00000001745	ASB18	ankyrin repeat and SOCS box containing 18

Heart	ENSAMEG00000001771	AKR1C8P	aldo-keto reductase family 1 member C8, pseudogene
Heart	ENSAMEG00000001779	GBX2	gastrulation brain homeobox 2
Heart	ENSAMEG00000001819	NDUFV3	NADH:ubiquinone oxidoreductase subunit V3
Heart	ENSAMEG00000001864	STAU2	staufen double-stranded RNA binding protein 2
Heart	ENSAMEG00000001897	PPP2R3B	protein phosphatase 2 regulatory subunit B''beta
Heart	ENSAMEG00000001943	PLIN4	perilipin 4
Heart	ENSAMEG00000002012	DPYSL5	dihydropyrimidinase like 5
Heart	ENSAMEG00000002134	CACNA1D	calcium voltage-gated channel subunit alpha1 D
Heart	ENSAMEG00000002200	ST14	suppression of tumorigenicity 14
Heart	ENSAMEG00000002238	DRC1	dynein regulatory complex subunit 1
Heart	ENSAMEG00000002250	TMEM144	transmembrane protein 144
Heart	ENSAMEG00000002265	METTL7A	methyltransferase like 7A
Heart	ENSAMEG00000002298	NT5M	5',3'-nucleotidase, mitochondrial
Heart	ENSAMEG00000002387	--	--
Heart	ENSAMEG00000002489	SLC25A35	solute carrier family 25 member 35
Heart	ENSAMEG00000002508	TMEM45B	transmembrane protein 45B
Heart	ENSAMEG00000002547	ANK1	ankyrin 1
Heart	ENSAMEG00000002550	GALNT3	polypeptide N-acetylgalactosaminyltransferase 3
Heart	ENSAMEG00000002556	KCNJ5	potassium voltage-gated channel subfamily J member 5
Heart	ENSAMEG00000002573	LOC100468405	N-acetyllactosaminide beta-1,6-N-acetylglucosaminyl-transferase
Heart	ENSAMEG00000002575	CFAP65	cilia and flagella associated protein 65
Heart	ENSAMEG00000002577	PPP1R3A	protein phosphatase 1 regulatory subunit 3A
Heart	ENSAMEG00000002582	RYR2	ryanodine receptor 2
Heart	ENSAMEG00000002608	PLCH1	phospholipase C eta 1
Heart	ENSAMEG00000002674	SPATA6L	spermatogenesis associated 6 like
Heart	ENSAMEG00000002676	PRKAG3	protein kinase AMP-activated non-catalytic subunit gamma 3
Heart	ENSAMEG00000002701	NAT16	N-acetyltransferase 16 (putative)
Heart	ENSAMEG00000002706	MOGAT3	monoacylglycerol O-acyltransferase 3
Heart	ENSAMEG00000002804	SDC1	syndecan 1
Heart	ENSAMEG00000002890	MIOX	myo-inositol oxygenase
Heart	ENSAMEG00000002953	RNF150	ring finger protein 150
Heart	ENSAMEG00000002973	FHL1	four and a half LIM domains 1
Heart	ENSAMEG00000002999	CPT1B	carnitine palmitoyltransferase 1B
Heart	ENSAMEG00000003000	PINK1	PTEN induced putative kinase 1
Heart	ENSAMEG00000003026	ABCC8	ATP binding cassette subfamily C member 8
Heart	ENSAMEG00000003040	ARID3A	AT-rich interaction domain 3A
Heart	ENSAMEG00000003064	GPR39	G protein-coupled receptor 39
Heart	ENSAMEG00000003082	PDLIM2	PDLIM2
Heart	ENSAMEG00000003158	ITGA4	integrin subunit alpha 4
Heart	ENSAMEG00000003181	USH1C	USH1 protein network component harmonin
Heart	ENSAMEG00000003204	NIPAL1	NIPA like domain containing 1

Heart	ENSAMEG00000003217	PAOX	polyamine oxidase
Heart	ENSAMEG00000003272	CORIN	corin, serine peptidase
Heart	ENSAMEG00000003321	SHC3	SHC adaptor protein 3
Heart	ENSAMEG00000003341	LGI3	leucine rich repeat LGI family member 3
Heart	ENSAMEG00000003360	REEP4	receptor accessory protein 4
Heart	ENSAMEG00000003381	FPGT	fucose-1-phosphate guanylyltransferase
Heart	ENSAMEG00000003388	--	--
Heart	ENSAMEG00000003417	TNNT1	troponin T1, slow skeletal type
Heart	ENSAMEG00000003421	--	--
Heart	ENSAMEG00000003423	GPNMB	glycoprotein nmb
Heart	ENSAMEG00000003436	PYGB	glycogen phosphorylase B
Heart	ENSAMEG00000003454	ADAMTSL5	ADAMTS like 5
Heart	ENSAMEG00000003486	TNNI3	troponin I3, cardiac type
Heart	ENSAMEG00000003492	SLC44A5	solute carrier family 44 member 5
Heart	ENSAMEG00000003512	MREG	melanoregulin
Heart	ENSAMEG00000003543	--	--
Heart	ENSAMEG00000003546	SPTB	spectrin beta, erythrocytic
Heart	ENSAMEG00000003591	TMEM26	transmembrane protein 26
Heart	ENSAMEG00000003633	CDS1	CDP-diacylglycerol synthase 1
Heart	ENSAMEG00000003736	ACE	angiotensin I converting enzyme
Heart	ENSAMEG00000003762	COQ10A	coenzyme Q10A
Heart	ENSAMEG00000003817	COL4A3	collagen type IV alpha 3 chain
Heart	ENSAMEG00000003831	MYBL2	MYB proto-oncogene like 2
Heart	ENSAMEG00000003853	SBK3	SH3 domain binding kinase family member 3
Heart	ENSAMEG00000003860	CCDC152	coiled-coil domain containing 152
Heart	ENSAMEG00000003863	--	--
Heart	ENSAMEG00000003907	JPH2	junctophilin 2
Heart	ENSAMEG00000003928	CLEC1B	C-type lectin domain family 1 member B
Heart	ENSAMEG00000004085	CORO6	coronin 6
Heart	ENSAMEG00000004173	--	--
Heart	ENSAMEG00000004179	CRYAB	Ailuropoda melanoleuca crystallin alpha B (CRYAB), mRNA.
Heart	ENSAMEG00000004234	LRTM2	leucine rich repeats and transmembrane domains 2
Heart	ENSAMEG00000004245	GMPR	guanosine monophosphate reductase
Heart	ENSAMEG00000004263	WNT5B	Wnt family member 5B
Heart	ENSAMEG00000004278	ABHD15	abhydrolase domain containing 15
Heart	ENSAMEG00000004365	PHLDB3	pleckstrin homology like domain family B member 3
Heart	ENSAMEG00000004412	MYCL	MYCL proto-oncogene, bHLH transcription factor
Heart	ENSAMEG00000004418	PRKCG	protein kinase C gamma
Heart	ENSAMEG00000004451	CACNG6	calcium voltage-gated channel auxiliary subunit gamma 6
Heart	ENSAMEG00000004467	APOBEC2	apolipoprotein B mRNA editing enzyme catalytic subunit 2
Heart	ENSAMEG00000004517	ASB15	ankyrin repeat and SOCS box containing 15
Heart	ENSAMEG00000004543	LMOD2	leiomodin 2
Heart	ENSAMEG00000004573	ATP1A3	ATPase Na+/K+ transporting subunit alpha 3

Heart	ENSAMEG00000004596	CAND2	cullin associated and neddylation dissociated 2 (putative)
Heart	ENSAMEG00000004640	IQSEC3	IQ motif and Sec7 domain 3
Heart	ENSAMEG00000004687	DCUN1D2	defective in cullin neddylation 1 domain containing 2
Heart	ENSAMEG00000004699	--	--
Heart	ENSAMEG00000004712	ADPRHL1	ADP-ribosylhydrolase like 1
Heart	ENSAMEG00000004756	CAMK2A	calcium/calmodulin dependent protein kinase II alpha
Heart	ENSAMEG00000004856	GPAT3	glycerol-3-phosphate acyltransferase 3
Heart	ENSAMEG00000004865	ALDOC	aldolase, fructose-bisphosphate C
Heart	ENSAMEG00000004894	ARHGAP9	Rho GTPase activating protein 9
Heart	ENSAMEG00000004913	--	--
Heart	ENSAMEG00000004945	CCDC136	coiled-coil domain containing 136
Heart	ENSAMEG00000004952	FLNC	filamin C
Heart	ENSAMEG00000005005	--	--
Heart	ENSAMEG00000005018	LOC100464625	placenta-specific gene 8 protein
Heart	ENSAMEG00000005152	PHOSPHO1	phosphoethanolamine/phosphocholine phosphatase
Heart	ENSAMEG00000005175	SLC26A10	solute carrier family 26 member 10
Heart	ENSAMEG00000005179	TLR3	toll like receptor 3
Heart	ENSAMEG00000005186	ACTC1	actin, alpha, cardiac muscle 1
Heart	ENSAMEG00000005191	SORBS2	sorbin and SH3 domain containing 2
Heart	ENSAMEG00000005193	ENPP5	ectonucleotide pyrophosphatase/phosphodiesterase 5 (putative)
Heart	ENSAMEG00000005244	--	--
Heart	ENSAMEG00000005259	FOLH1	folate hydrolase 1
Heart	ENSAMEG00000005285	STRIP2	striatin interacting protein 2
Heart	ENSAMEG00000005321	ANKRD23	ankyrin repeat domain 23
Heart	ENSAMEG00000005409	RAB11FIP4	RAB11 family interacting protein 4
Heart	ENSAMEG00000005530	BICDL2	BICD family like cargo adaptor 2
Heart	ENSAMEG00000005545	MMP25	matrix metallopeptidase 25
Heart	ENSAMEG00000005568	SYNM	synemin
Heart	ENSAMEG00000005581	HAAO	3-hydroxyanthranilate 3,4-dioxygenase
Heart	ENSAMEG00000005601	KCNJ3	potassium voltage-gated channel subfamily J member 3
Heart	ENSAMEG00000005631	GNG7	G protein subunit gamma 7
Heart	ENSAMEG00000005637	LSMEM1	leucine rich single-pass membrane protein 1
Heart	ENSAMEG00000005675	WIPF3	WAS/WASL interacting protein family member 3
Heart	ENSAMEG00000005679	CALHM6	calcium homeostasis modulator family member 6
Heart	ENSAMEG00000005697	DENND2D	DENN domain containing 2D
Heart	ENSAMEG00000005814	KLHL31	kelch like family member 31
Heart	ENSAMEG00000005833	CITED1	Cbp/p300 interacting transactivator with Glu/Asp rich carboxy-terminal domain 1
Heart	ENSAMEG00000005890	PDLIM5	PDZ and LIM domain 5
Heart	ENSAMEG00000005897	DKK3	dickkopf WNT signaling pathway inhibitor 3
Heart	ENSAMEG00000005900	TRPV4	transient receptor potential cation channel subfamily V member 4

Heart	ENSAMEG00000005941	DHX58	DExH-box helicase 58
Heart	ENSAMEG00000005948	SNX10	sorting nexin 10
Heart	ENSAMEG00000006005	USP28	ubiquitin specific peptidase 28
Heart	ENSAMEG00000006050	EPM2A	EPM2A, laforin glucan phosphatase
Heart	ENSAMEG00000006056	DAPK3	death associated protein kinase 3
Heart	ENSAMEG00000006071	NEBL	nebulette
Heart	ENSAMEG00000006223	EPCAM	epithelial cell adhesion molecule
Heart	ENSAMEG00000006291	PPP4R4	protein phosphatase 4 regulatory subunit 4
Heart	ENSAMEG00000006321	--	--
Heart	ENSAMEG00000006323	ENO3	enolase 3
Heart	ENSAMEG00000006354	FAH	fumarylacetoacetate hydrolase
Heart	ENSAMEG00000006366	SAPCD1	suppressor APC domain containing 1
Heart	ENSAMEG00000006372	VWA7	von Willebrand factor A domain containing 7
Heart	ENSAMEG00000006380	--	--
Heart	ENSAMEG00000006439	EGLN3	egl-9 family hypoxia inducible factor 3
Heart	ENSAMEG00000006482	FAM83H	family with sequence similarity 83 member H
Heart	ENSAMEG00000006487	CDH13	cadherin 13
Heart	ENSAMEG00000006563	ADRA1B	adrenoceptor alpha 1B
Heart	ENSAMEG00000006607	--	--
Heart	ENSAMEG00000006647	--	--
Heart	ENSAMEG00000006681	--	--
Heart	ENSAMEG00000006692	CRHR2	corticotropin releasing hormone receptor 2
Heart	ENSAMEG00000006710	LOC100476424	death-associated protein kinase 1
Heart	ENSAMEG00000006735	SCD	stearoyl-CoA desaturase
Heart	ENSAMEG00000006751	PGD	phosphogluconate dehydrogenase
Heart	ENSAMEG00000006816	LOC105240557	sushi domain-containing protein 1
Heart	ENSAMEG00000006823	BAG2	BCL2 associated athanogene 2
Heart	ENSAMEG00000006849	ESYT3	extended synaptotagmin 3
Heart	ENSAMEG00000006972	WWC1	WW and C2 domain containing 1
Heart	ENSAMEG00000006976	PDE1C	phosphodiesterase 1C
Heart	ENSAMEG00000006983	ARHGDIG	Rho GDP dissociation inhibitor gamma
Heart	ENSAMEG00000006994	PDIA2	protein disulfide isomerase family A member 2
Heart	ENSAMEG00000007003	CLTB	clathrin light chain B
Heart	ENSAMEG00000007007	DDN	dendrin
Heart	ENSAMEG00000007033	--	--
Heart	ENSAMEG00000007055	MFAP5	microfibril associated protein 5
Heart	ENSAMEG00000007062	TMEM171	transmembrane protein 171
Heart	ENSAMEG00000007087	MAATS1	MYCBP associated and testis expressed 1
Heart	ENSAMEG00000007101	DENND1C	DENN domain containing 1C
Heart	ENSAMEG00000007118	GDF3	growth differentiation factor 3
Heart	ENSAMEG00000007148	POPDC2	popeye domain containing 2
Heart	ENSAMEG00000007223	SLC7A7	solute carrier family 7 member 7
Heart	ENSAMEG00000007224	TPM1	tropomyosin 1

Heart	ENSAMEG00000007231	AK5	adenylate kinase 5
Heart	ENSAMEG00000007238	SMTNL2	smoothelin like 2
Heart	ENSAMEG00000007248	KCNK1	potassium two pore domain channel subfamily K member 1
Heart	ENSAMEG00000007311	--	--
Heart	ENSAMEG00000007328	XK	X-linked Kx blood group
Heart	ENSAMEG00000007337	NEXN	nexilin F-actin binding protein
Heart	ENSAMEG00000007362	MYBPC3	myosin binding protein C, cardiac
Heart	ENSAMEG00000007430	SHANK2	SH3 and multiple ankyrin repeat domains 2
Heart	ENSAMEG00000007487	TRDN	triadin
Heart	ENSAMEG00000007526	SYNC	syncoilin, intermediate filament protein
Heart	ENSAMEG00000007591	RAB31	RAB31, member RAS oncogene family
Heart	ENSAMEG00000007739	CDH1	cadherin 1
Heart	ENSAMEG00000007820	CDH3	cadherin 3
Heart	ENSAMEG00000007918	NDUFA11	NADH:ubiquinone oxidoreductase subunit A11
Heart	ENSAMEG00000007927	ADSSL1	adenylosuccinate synthase like 1
Heart	ENSAMEG00000007941	ZC2HC1A	zinc finger C2HC-type containing 1A
Heart	ENSAMEG00000008002	MAP3K9	mitogen-activated protein kinase kinase kinase 9
Heart	ENSAMEG00000008013	--	--
Heart	ENSAMEG00000008041	LNX1	ligand of numb-protein X 1
Heart	ENSAMEG00000008069	SLC12A7	solute carrier family 12 member 7
Heart	ENSAMEG00000008182	SGCB	sarcoglycan beta
Heart	ENSAMEG00000008217	--	--
Heart	ENSAMEG00000008255	--	--
Heart	ENSAMEG00000008308	TADA2B	transcriptional adaptor 2B
Heart	ENSAMEG00000008322	ID2	inhibitor of DNA binding 2
Heart	ENSAMEG00000008339	SLC9A3	solute carrier family 9 member A3
Heart	ENSAMEG00000008373	MBOAT2	membrane bound O-acyltransferase domain containing 2
Heart	ENSAMEG00000008429	--	--
Heart	ENSAMEG00000008481	DUSP26	dual specificity phosphatase 26
Heart	ENSAMEG00000008489	RPL3L	ribosomal protein L3 like
Heart	ENSAMEG00000008513	RAMP1	receptor activity modifying protein 1
Heart	ENSAMEG00000008518	SCTR	secretin receptor
Heart	ENSAMEG00000008533	STXBP2	syntaxin binding protein 2
Heart	ENSAMEG00000008571	FRY	FRY microtubule binding protein
Heart	ENSAMEG00000008587	PCDH8	protocadherin 8
Heart	ENSAMEG00000008605	RBFOX1	RNA binding fox-1 homolog 1
Heart	ENSAMEG00000008717	FBXO17	F-box protein 17
Heart	ENSAMEG00000008743	SLC25A4	solute carrier family 25 member 4
Heart	ENSAMEG00000008763	TNNC1	troponin C, slow skeletal and cardiac muscles
Heart	ENSAMEG00000008769	ASB5	ankyrin repeat and SOCS box containing 5
Heart	ENSAMEG00000008809	RGMA	repulsive guidance molecule BMP co-receptor a
Heart	ENSAMEG00000008818	RASAL1	RAS protein activator like 1

Heart	ENSAMEG00000008838	COLQ	collagen like tail subunit of asymmetric acetylcholinesterase
Heart	ENSAMEG00000008850	THSD4	thrombospondin type 1 domain containing 4
Heart	ENSAMEG00000008879	PKP2	plakophilin 2
Heart	ENSAMEG00000008898	CYP1A1	cytochrome P450 family 1 subfamily A member 1
Heart	ENSAMEG00000008901	LRRC39	leucine rich repeat containing 39
Heart	ENSAMEG00000008917	TRMT13	tRNA methyltransferase 13 homolog
Heart	ENSAMEG00000008934	MTUS2	microtubule associated scaffold protein 2
Heart	ENSAMEG00000009011	USP50	ubiquitin specific peptidase 50
Heart	ENSAMEG00000009119	KIF26B	kinesin family member 26B
Heart	ENSAMEG00000009131	ACTA1	actin, alpha 1, skeletal muscle
Heart	ENSAMEG00000009148	--	--
Heart	ENSAMEG00000009153	TMEM35A	transmembrane protein 35A
Heart	ENSAMEG00000009193	DMXL2	Dmx like 2
Heart	ENSAMEG00000009200	CAMK2D	calcium/calmodulin dependent protein kinase II delta
Heart	ENSAMEG00000009310	ABRA	actin binding Rho activating protein
Heart	ENSAMEG00000009361	MYH7B	myosin heavy chain 7B
Heart	ENSAMEG00000009393	YBX3	Y-box binding protein 3
Heart	ENSAMEG00000009440	LOC100472452	cytochrome P450 2D15
Heart	ENSAMEG00000009448	SYTL3	synaptotagmin like 3
Heart	ENSAMEG00000009471	--	--
Heart	ENSAMEG00000009531	MCCD1	mitochondrial coiled-coil domain 1
Heart	ENSAMEG00000009536	FAM110C	family with sequence similarity 110 member C
Heart	ENSAMEG00000009581	SYNJ2	synaptojanin 2
Heart	ENSAMEG00000009592	HEY2	hes related family bHLH transcription factor with YRPW motif 2
Heart	ENSAMEG00000009617	SMIM5	small integral membrane protein 5
Heart	ENSAMEG00000009672	RBM47	RNA binding motif protein 47
Heart	ENSAMEG00000009691	BCAR3	BCAR3, NSP family adaptor protein
Heart	ENSAMEG00000009740	OSBPL10	oxysterol binding protein like 10
Heart	ENSAMEG00000009835	FUCA1	alpha-L-fucosidase 1
Heart	ENSAMEG00000009857	RNF207	ring finger protein 207
Heart	ENSAMEG00000009867	LOC105236178	putative ATP-dependent RNA helicase TDRD12
Heart	ENSAMEG00000009873	NPPB	natriuretic peptide B
Heart	ENSAMEG00000009891	MYOM3	myomesin 3
Heart	ENSAMEG00000009901	GRB7	growth factor receptor bound protein 7
Heart	ENSAMEG00000009912	OPN4	opsin 4
Heart	ENSAMEG00000009931	TTC22	tetratricopeptide repeat domain 22
Heart	ENSAMEG00000009934	REEP1	receptor accessory protein 1
Heart	ENSAMEG00000009941	LDB3	LIM domain binding 3
Heart	ENSAMEG00000009967	GRAMD2B	GRAM domain containing 2B
Heart	ENSAMEG00000009998	EMILIN2	elastin microfibril interfacer 2
Heart	ENSAMEG00000010023	MAP7	microtubule associated protein 7

Heart	ENSAMEG00000010030	PNMT	phenylethanolamine N-methyltransferase
Heart	ENSAMEG00000010033	MFN2	mitofusin 2
Heart	ENSAMEG00000010035	SMYD1	SET and MYND domain containing 1
Heart	ENSAMEG00000010039	TCAP	titin-cap
Heart	ENSAMEG00000010068	MMP7	matrix metallopeptidase 7
Heart	ENSAMEG00000010085	SV2A	synaptic vesicle glycoprotein 2A
Heart	ENSAMEG00000010097	MYL9	myosin light chain 9
Heart	ENSAMEG00000010108	TMEM38A	transmembrane protein 38A
Heart	ENSAMEG00000010121	LGALS1	galectin 1
Heart	ENSAMEG00000010132	TNFRSF8	TNF receptor superfamily member 8
Heart	ENSAMEG00000010183	CHST15	carbohydrate sulfotransferase 15
Heart	ENSAMEG00000010197	FGF1	fibroblast growth factor 1
Heart	ENSAMEG00000010277	TANC1	tetratricopeptide repeat, ankyrin repeat and coiled-coil containing 1
Heart	ENSAMEG00000010346	--	--
Heart	ENSAMEG00000010380	TRIM63	tripartite motif containing 63
Heart	ENSAMEG00000010420	ANO9	anoctamin 9
Heart	ENSAMEG00000010473	BZW2	basic leucine zipper and W2 domains 2
Heart	ENSAMEG00000010512	CEP85	centrosomal protein 85
Heart	ENSAMEG00000010530	MBP	myelin basic protein
Heart	ENSAMEG00000010550	GCOM1	GRINL1A complex locus 1
Heart	ENSAMEG00000010570	MYOM1	myomesin 1
Heart	ENSAMEG00000010606	EPOR	erythropoietin receptor
Heart	ENSAMEG00000010611	HACD1	3-hydroxyacyl-CoA dehydratase 1
Heart	ENSAMEG00000010622	KLHL41	kelch like family member 41
Heart	ENSAMEG00000010699	EPS8L2	EPS8 like 2
Heart	ENSAMEG00000010803	FAM174B	family with sequence similarity 174 member B
Heart	ENSAMEG00000010852	CASQ2	calsequestrin 2
Heart	ENSAMEG00000010858	TACC2	transforming acidic coiled-coil containing protein 2
Heart	ENSAMEG00000010862	SRL	sarcalumenin
Heart	ENSAMEG00000010870	STYXL1	serine/threonine/tyrosine interacting like 1
Heart	ENSAMEG00000010890	GYG1	glycogenin 1
Heart	ENSAMEG00000010896	USP13	ubiquitin specific peptidase 13
Heart	ENSAMEG00000010933	IRX4	iroquois homeobox 4
Heart	ENSAMEG00000010959	FGFR2	fibroblast growth factor receptor 2
Heart	ENSAMEG00000010966	CP	ceruloplasmin
Heart	ENSAMEG00000011080	ARL6IP1	ADP ribosylation factor like GTPase 6 interacting protein 1
Heart	ENSAMEG00000011145	FBXO32	F-box protein 32
Heart	ENSAMEG00000011186	KLHL38	kelch like family member 38
Heart	ENSAMEG00000011223	PROB1	proline rich basic protein 1
Heart	ENSAMEG00000011266	SESN2	sestrin 2
Heart	ENSAMEG00000011277	CUNH4orf19	chromosome unknown C4orf19 homolog
Heart	ENSAMEG00000011367	TRNAU1AP	tRNA selenocysteine 1 associated protein 1

Heart	ENSAMEG00000011403	F5	coagulation factor V
Heart	ENSAMEG00000011404	ROBO2	roundabout guidance receptor 2
Heart	ENSAMEG00000011407	LRRC7	leucine rich repeat containing 7
Heart	ENSAMEG00000011433	BVES	blood vessel epicardial substance
Heart	ENSAMEG00000011452	CDNF	cerebral dopamine neurotrophic factor
Heart	ENSAMEG00000011509	--	--
Heart	ENSAMEG00000011514	TRAK1	trafficking kinesin protein 1
Heart	ENSAMEG00000011537	PTHLH	parathyroid hormone like hormone
Heart	ENSAMEG00000011558	C3orf18	chromosome 3 open reading frame 18
Heart	ENSAMEG00000011616	NT5C1A	5'-nucleotidase, cytosolic IA
Heart	ENSAMEG00000011654	PDE4DIP	phosphodiesterase 4D interacting protein
Heart	ENSAMEG00000011759	ART3	ADP-ribosyltransferase 3
Heart	ENSAMEG00000011766	ASB14	ankyrin repeat and SOCS box containing 14
Heart	ENSAMEG00000011773	TXLNB	taxilin beta
Heart	ENSAMEG00000011778	LSMEM2	leucine rich single-pass membrane protein 2
Heart	ENSAMEG00000011779	HSPB7	heat shock protein family B (small) member 7
Heart	ENSAMEG00000011799	ABRACL	ABRA C-terminal like
Heart	ENSAMEG00000011804	ACSF2	acyl-CoA synthetase family member 2
Heart	ENSAMEG00000011855	ITGA7	integrin subunit alpha 7
Heart	ENSAMEG00000011861	SLMAP	sarcolemma associated protein
Heart	ENSAMEG00000011870	SLC38A3	solute carrier family 38 member 3
Heart	ENSAMEG00000011890	LOC100470069	protein phosphatase 1 regulatory subunit 12B
Heart	ENSAMEG00000011902	SSPN	sarcospan
Heart	ENSAMEG00000011916	CDH2	cadherin 2
Heart	ENSAMEG00000012004	TLN2	talin 2
Heart	ENSAMEG00000012039	MRAP2	melanocortin 2 receptor accessory protein 2
Heart	ENSAMEG00000012057	SGCA	sarcoglycan alpha
Heart	ENSAMEG00000012065	--	--
Heart	ENSAMEG00000012103	DHRS7C	dehydrogenase/reductase 7C
Heart	ENSAMEG00000012137	CLEC3B	C-type lectin domain family 3 member B
Heart	ENSAMEG00000012143	CDCP1	CUB domain containing protein 1
Heart	ENSAMEG00000012160	TNNT2	troponin T2, cardiac type
Heart	ENSAMEG00000012195	S100A1	S100 calcium binding protein A1
Heart	ENSAMEG00000012237	RPRD1B	regulation of nuclear pre-mRNA domain containing 1B
Heart	ENSAMEG00000012267	PPFIA4	PTPRF interacting protein alpha 4
Heart	ENSAMEG00000012308	RANBP3L	RAN binding protein 3 like
Heart	ENSAMEG00000012312	CRIP3	cysteine rich protein 3
Heart	ENSAMEG00000012338	TRIM55	tripartite motif containing 55
Heart	ENSAMEG00000012346	ESPL1	extra spindle pole bodies like 1, separase
Heart	ENSAMEG00000012356	CAV3	caveolin 3
Heart	ENSAMEG00000012357	CHI3L1	chitinase 3 like 1
Heart	ENSAMEG00000012369	ADAMTS7	ADAM metallopeptidase with thrombospondin type 1 motif 7

Heart	ENSAMEG00000012393	MST1	macrophage stimulating 1
Heart	ENSAMEG00000012440	HHATL	hedgehog acyltransferase like
Heart	ENSAMEG00000012471	KLHL40	kelch like family member 40
Heart	ENSAMEG00000012478	LRRC2	leucine rich repeat containing 2
Heart	ENSAMEG00000012484	ZBTB47	zinc finger and BTB domain containing 47
Heart	ENSAMEG00000012508	C19orf47	chromosome 19 open reading frame 47
Heart	ENSAMEG00000012517	TESC	tescalcin
Heart	ENSAMEG00000012518	ACSBG1	acyl-CoA synthetase bubblegum family member 1
Heart	ENSAMEG00000012531	SEC22C	SEC22 homolog C, vesicle trafficking protein
Heart	ENSAMEG00000012540	DAG1	dystroglycan 1
Heart	ENSAMEG00000012543	--	--
Heart	ENSAMEG00000012553	--	--
Heart	ENSAMEG00000012554	CKM	creatine kinase, M-type
Heart	ENSAMEG00000012559	--	--
Heart	ENSAMEG00000012576	DNAJA4	DnaJ heat shock protein family (Hsp40) member A4
Heart	ENSAMEG00000012586	MYL3	myosin light chain 3
Heart	ENSAMEG00000012612	NEURL2	neuralized E3 ubiquitin protein ligase 2
Heart	ENSAMEG00000012726	DEFB1	beta-defensin 103
Heart	ENSAMEG00000012737	APOC1	apolipoprotein C1
Heart	ENSAMEG00000012740	APOE	apolipoprotein E
Heart	ENSAMEG00000012755	SH3BGRL2	SH3 domain binding glutamate rich protein like 2
Heart	ENSAMEG00000012814	PRPH2	peripherin 2
Heart	ENSAMEG00000012933	CACNB2	calcium voltage-gated channel auxiliary subunit beta 2
Heart	ENSAMEG00000012970	JOSD2	Josephin domain containing 2
Heart	ENSAMEG00000012992	SNTA1	syntrophin alpha 1
Heart	ENSAMEG00000013171	ASB12	ankyrin repeat and SOCS box containing 12
Heart	ENSAMEG00000013245	TMEM182	transmembrane protein 182
Heart	ENSAMEG00000013259	MAMDC4	MAM domain containing 4
Heart	ENSAMEG00000013297	FHL2	four and a half LIM domains 2
Heart	ENSAMEG00000013354	SOX11	SRY-box 11
Heart	ENSAMEG00000013371	PARD6B	par-6 family cell polarity regulator beta
Heart	ENSAMEG00000013410	TRIM6	tripartite motif containing 6
Heart	ENSAMEG00000013468	C1orf21	chromosome 1 open reading frame 21
Heart	ENSAMEG00000013506	PRKACB	protein kinase cAMP-activated catalytic subunit beta
Heart	ENSAMEG00000013510	CENPP	centromere protein P
Heart	ENSAMEG00000013511	PTX3	pentraxin 3
Heart	ENSAMEG00000013527	MLF1	myeloid leukemia factor 1
Heart	ENSAMEG00000013533	SMARCD3	SWI/SNF related, matrix associated, actin dependent regulator of chromatin, subfamily d, member 3
Heart	ENSAMEG00000013547	--	--
Heart	ENSAMEG00000013559	C1RL	complement C1r subcomponent like
Heart	ENSAMEG00000013564	TMEM52	transmembrane protein 52
Heart	ENSAMEG00000013581	DNASE2B	deoxyribonuclease 2 beta

Heart	ENSAMEG00000013620	IQCA1L	IQ motif containing with AAA domain 1 like
Heart	ENSAMEG00000013644	ASB10	ankyrin repeat and SOCS box containing 10
Heart	ENSAMEG00000013673	--	--
Heart	ENSAMEG00000013677	--	--
Heart	ENSAMEG00000013680	PDGFRL	platelet derived growth factor receptor like
Heart	ENSAMEG00000013682	DERL3	derlin 3
Heart	ENSAMEG00000013784	LOC100480698	glutathione S-transferase theta-1
Heart	ENSAMEG00000013791	PFKM	phosphofructokinase, muscle
Heart	ENSAMEG00000013825	MYL2	myosin light chain 2
Heart	ENSAMEG00000013845	CCDC63	coiled-coil domain containing 63
Heart	ENSAMEG00000013928	PNLIP	pancreatic lipase
Heart	ENSAMEG00000013991	--	--
Heart	ENSAMEG00000013992	KCNH2	potassium voltage-gated channel subfamily H member 2
Heart	ENSAMEG00000013993	GNB3	G protein subunit beta 3
Heart	ENSAMEG00000014004	PNLIPRP2	pancreatic lipase related protein 2 (gene/pseudogene)
Heart	ENSAMEG00000014007	ATP2A2	ATPase sarcoplasmic/endoplasmic reticulum Ca2+ transporting 2
Heart	ENSAMEG00000014015	NMB	neuromedin B
Heart	ENSAMEG00000014026	TTC39C	tetratricopeptide repeat domain 39C
Heart	ENSAMEG00000014038	ALPK3	alpha kinase 3
Heart	ENSAMEG00000014046	ENO4	enolase family member 4
Heart	ENSAMEG00000014060	SHTN1	shootin 1
Heart	ENSAMEG00000014092	SGCG	sarcoglycan gamma
Heart	ENSAMEG00000014116	SH3BGR	SH3 domain binding glutamate rich protein
Heart	ENSAMEG00000014131	LGALS3BP	galectin 3 binding protein
Heart	ENSAMEG00000014164	CASQ1	calsequestrin 1
Heart	ENSAMEG00000014165	--	--
Heart	ENSAMEG00000014235	HAND2	heart and neural crest derivatives expressed 2
Heart	ENSAMEG00000014238	--	--
Heart	ENSAMEG00000014245	ILDR1	immunoglobulin like domain containing receptor 1
Heart	ENSAMEG00000014250	GLRB	glycine receptor beta
Heart	ENSAMEG00000014341	--	--
Heart	ENSAMEG00000014348	EREG	epiregulin
Heart	ENSAMEG00000014363	HRC	histidine rich calcium binding protein
Heart	ENSAMEG00000014365	EDA2R	ectodysplasin A2 receptor
Heart	ENSAMEG00000014426	MRPL51	mitochondrial ribosomal protein L51
Heart	ENSAMEG00000014490	IL5RA	interleukin 5 receptor subunit alpha
Heart	ENSAMEG00000014491	GALNT16	polypeptide N-acetylgalactosaminyltransferase 16
Heart	ENSAMEG00000014525	MYOM2	myomesin 2
Heart	ENSAMEG00000014564	RFFL	ring finger and FYVE like domain containing E3 ubiquitin protein ligase
Heart	ENSAMEG00000014582	FTL	Ailuropoda melanoleuca ferritin light chain (FTL), mRNA.
Heart	ENSAMEG00000014616	DHDH	dihydrodiol dehydrogenase

Heart	ENSAMEG00000014637	UNC45B	unc-45 myosin chaperone B
Heart	ENSAMEG00000014670	KERA	keratocan
Heart	ENSAMEG00000014674	LUM	lumican
Heart	ENSAMEG00000014684	MYL4	myosin light chain 4
Heart	ENSAMEG00000014750	GRIA3	glutamate ionotropic receptor AMPA type subunit 3
Heart	ENSAMEG00000014822	RASL10B	RAS like family 10 member B
Heart	ENSAMEG00000014823	FAM241A	family with sequence similarity 241 member A
Heart	ENSAMEG00000014870	POLQ	DNA polymerase theta
Heart	ENSAMEG00000014942	CAVIN4	caveolae associated protein 4
Heart	ENSAMEG00000014952	FBXO40	F-box protein 40
Heart	ENSAMEG00000014960	KLHL30	kelch like family member 30
Heart	ENSAMEG00000014978	ABCB9	ATP binding cassette subfamily B member 9
Heart	ENSAMEG00000014986	ABLIM1	actin binding LIM protein 1
Heart	ENSAMEG00000015037	LOC100478250	solute carrier family 22 member 3
Heart	ENSAMEG00000015072	ABLIM2	actin binding LIM protein family member 2
Heart	ENSAMEG00000015076	--	--
Heart	ENSAMEG00000015099	KBTBD12	kelch repeat and BTB domain containing 12
Heart	ENSAMEG00000015190	KIAA1147	KIAA1147
Heart	ENSAMEG00000015212	ALPK2	alpha kinase 2
Heart	ENSAMEG00000015250	--	--
Heart	ENSAMEG00000015252	RILPL1	Rab interacting lysosomal protein like 1
Heart	ENSAMEG00000015302	NRAP	nebulin related anchoring protein
Heart	ENSAMEG00000015361	MPZ	myelin protein zero
Heart	ENSAMEG00000015374	C3orf14	chromosome 3 open reading frame 14
Heart	ENSAMEG00000015381	PCP4L1	Purkinje cell protein 4 like 1
Heart	ENSAMEG00000015419	ACSL5	acyl-CoA synthetase long chain family member 5
Heart	ENSAMEG00000015633	CMYA5	cardiomyopathy associated 5
Heart	ENSAMEG00000015667	--	--
Heart	ENSAMEG00000015670	RBM20	RNA binding motif protein 20
Heart	ENSAMEG00000015685	AKAP6	A-kinase anchoring protein 6
Heart	ENSAMEG00000015719	JPH1	junctophilin 1
Heart	ENSAMEG00000015725	TIGAR	TP53 induced glycolysis regulatory phosphatase
Heart	ENSAMEG00000015746	ENAH	ENAH, actin regulator
Heart	ENSAMEG00000015788	SMYD2	SET and MYND domain containing 2
Heart	ENSAMEG00000015798	ZNF106	zinc finger protein 106
Heart	ENSAMEG00000015818	ASB2	ankyrin repeat and SOCS box containing 2
Heart	ENSAMEG00000015838	CKMT2	creatine kinase, mitochondrial 2
Heart	ENSAMEG00000015891	TMEM65	transmembrane protein 65
Heart	ENSAMEG00000015901	DNAJB5	DnaJ heat shock protein family (Hsp40) member B5
Heart	ENSAMEG00000015907	PHF24	PHD finger protein 24
Heart	ENSAMEG00000015921	ACE2	angiotensin I converting enzyme 2
Heart	ENSAMEG00000015924	EYA4	EYA transcriptional coactivator and phosphatase 4
Heart	ENSAMEG00000016006	SORBS1	sorbin and SH3 domain containing 1

Heart	ENSAMEG00000016056	PHKG1	phosphorylase kinase catalytic subunit gamma 1
Heart	ENSAMEG00000016081	ITGB1BP2	integrin subunit beta 1 binding protein 2
Heart	ENSAMEG00000016087	PDLIM1	PDZ and LIM domain 1
Heart	ENSAMEG00000016096	YIPF7	Yip1 domain family member 7
Heart	ENSAMEG00000016128	WSCD2	WSC domain containing 2
Heart	ENSAMEG00000016175	LDHB	lactate dehydrogenase B
Heart	ENSAMEG00000016190	AAK1	AP2 associated kinase 1
Heart	ENSAMEG00000016226	DGKD	diacylglycerol kinase delta
Heart	ENSAMEG00000016258	--	--
Heart	ENSAMEG00000016276	AIF1L	allograft inflammatory factor 1 like
Heart	ENSAMEG00000016284	COX6A2	cytochrome c oxidase subunit 6A2
Heart	ENSAMEG00000016286	--	--
Heart	ENSAMEG00000016321	MYOT	myotilin
Heart	ENSAMEG00000016331	MAOB	monoamine oxidase B
Heart	ENSAMEG00000016338	PLA2G4E	phospholipase A2 group IVE
Heart	ENSAMEG00000016352	PLPP7	phospholipid phosphatase 7 (inactive)
Heart	ENSAMEG00000016398	PHEX	phosphate regulating endopeptidase homolog X-linked
Heart	ENSAMEG00000016415	IRX6	iroquois homeobox 6
Heart	ENSAMEG00000016467	SMPX	small muscle protein, X-linked
Heart	ENSAMEG00000016475	FITM1	fat storage inducing transmembrane protein 1
Heart	ENSAMEG00000016476	KLHL34	kelch like family member 34
Heart	ENSAMEG00000016542	SLC2A4	solute carrier family 2 member 4
Heart	ENSAMEG00000016569	--	--
Heart	ENSAMEG00000016574	HHIP	hedgehog interacting protein
Heart	ENSAMEG00000016582	CACNA2D1	calcium voltage-gated channel auxiliary subunit alpha2delta 1
Heart	ENSAMEG00000016588	DHRS2	dehydrogenase/reductase 2
Heart	ENSAMEG00000016591	GNAO1	G protein subunit alpha o1
Heart	ENSAMEG00000016616	AP1G2	adaptor related protein complex 1 gamma 2 subunit
Heart	ENSAMEG00000016650	TNK1	tyrosine kinase non receptor 1
Heart	ENSAMEG00000016662	DPYD	dihydropyrimidine dehydrogenase
Heart	ENSAMEG00000016678	MYH7	myosin heavy chain 7
Heart	ENSAMEG00000016692	HAND1	heart and neural crest derivatives expressed 1
Heart	ENSAMEG00000016717	CDH8	cadherin 8
Heart	ENSAMEG00000016730	PDHA1	pyruvate dehydrogenase E1 alpha 1 subunit
Heart	ENSAMEG00000016731	KIF1A	kinesin family member 1A
Heart	ENSAMEG00000016742	SPEG	SPEG complex locus
Heart	ENSAMEG00000016743	SLC12A3	solute carrier family 12 member 3
Heart	ENSAMEG00000016756	CPLX1	complexin 1
Heart	ENSAMEG00000016767	ANKRD1	ankyrin repeat domain 1
Heart	ENSAMEG00000016775	FXR2	FMR1 autosomal homolog 2
Heart	ENSAMEG00000016787	HTR7	5-hydroxytryptamine receptor 7
Heart	ENSAMEG00000016792	TTC16	tetratricopeptide repeat domain 16

Heart	ENSAMEG00000016801	DES	desmin
Heart	ENSAMEG00000016819	SLC16A12	solute carrier family 16 member 12
Heart	ENSAMEG00000016827	SYNPO2L	synaptopodin 2 like
Heart	ENSAMEG00000016871	OBSL1	obscurin like 1
Heart	ENSAMEG00000016879	INHA	inhibin alpha subunit
Heart	ENSAMEG00000016900	EFNB3	ephrin B3
Heart	ENSAMEG00000016909	SLC4A3	solute carrier family 4 member 3
Heart	ENSAMEG00000016925	MYOZ2	myozenin 2
Heart	ENSAMEG00000016958	DGAT2	diacylglycerol O-acyltransferase 2
Heart	ENSAMEG00000017112	ASB11	ankyrin repeat and SOCS box containing 11
Heart	ENSAMEG00000017136	CDH16	cadherin 16
Heart	ENSAMEG00000017153	LOC105234678	catenin alpha-3
Heart	ENSAMEG00000017157	GPC5	glypican 5
Heart	ENSAMEG00000017159	RRAD	RRAD, Ras related glycolysis inhibitor and calcium channel regulator
Heart	ENSAMEG00000017177	RAB27B	RAB27B, member RAS oncogene family
Heart	ENSAMEG00000017262	NDRG4	NDRG family member 4
Heart	ENSAMEG00000017295	MCOLN2	mucolipin 2
Heart	ENSAMEG00000017354	DSP	desmoplakin
Heart	ENSAMEG00000017376	GATA5	GATA binding protein 5
Heart	ENSAMEG00000017417	MLIP	muscular LMNA interacting protein
Heart	ENSAMEG00000017433	NRN1	neuritin 1
Heart	ENSAMEG00000017441	TUBA8	tubulin alpha 8
Heart	ENSAMEG00000017469	FCMR	Fc fragment of IgM receptor
Heart	ENSAMEG00000017493	PPP1R27	protein phosphatase 1 regulatory subunit 27
Heart	ENSAMEG00000017522	PGAM2	phosphoglycerate mutase 2
Heart	ENSAMEG00000017553	--	--
Heart	ENSAMEG00000017554	PPM1L	protein phosphatase, Mg2+/Mn2+ dependent 1L
Heart	ENSAMEG00000017567	SH3RF2	SH3 domain containing ring finger 2
Heart	ENSAMEG00000017578	MYL7	myosin light chain 7
Heart	ENSAMEG00000017582	PLAC8L1	PLAC8 like 1
Heart	ENSAMEG00000017648	KCNIP2	potassium voltage-gated channel interacting protein 2
Heart	ENSAMEG00000017650	SCN5A	sodium voltage-gated channel alpha subunit 5
Heart	ENSAMEG00000017720	AP1S2	adaptor related protein complex 1 sigma 2 subunit
Heart	ENSAMEG00000017749	--	--
Heart	ENSAMEG00000017831	PYGM	glycogen phosphorylase, muscle associated
Heart	ENSAMEG00000017850	LRRC20	leucine rich repeat containing 20
Heart	ENSAMEG00000017870	MBOAT1	membrane bound O-acyltransferase domain containing 1
Heart	ENSAMEG00000017899	CLEC2L	C-type lectin domain family 2 member L
Heart	ENSAMEG00000017910	TSPAN15	tetraspanin 15
Heart	ENSAMEG00000017946	CSRP3	cysteine and glycine rich protein 3
Heart	ENSAMEG00000017964	PTGR1	prostaglandin reductase 1
Heart	ENSAMEG00000017984	IRF6	interferon regulatory factor 6

Heart	ENSAMEG00000017987	MYO18B	myosin XVIIIB
Heart	ENSAMEG00000018031	LEPR	leptin receptor
Heart	ENSAMEG00000018037	CAMTA1	calmodulin binding transcription activator 1
Heart	ENSAMEG00000018059	--	--
Heart	ENSAMEG00000018087	MYPN	myopalladin
Heart	ENSAMEG00000018115	PPP1R13B	protein phosphatase 1 regulatory subunit 13B
Heart	ENSAMEG00000018130	--	--
Heart	ENSAMEG00000018146	--	--
Heart	ENSAMEG00000018159	CKB	creatine kinase B
Heart	ENSAMEG00000018183	MSRB3	methionine sulfoxide reductase B3
Heart	ENSAMEG00000018258	PPP1R14C	protein phosphatase 1 regulatory inhibitor subunit 14C
Heart	ENSAMEG00000018339	FLVCR2	feline leukemia virus subgroup C cellular receptor family member 2
Heart	ENSAMEG00000018349	TGFB3	transforming growth factor beta 3
Heart	ENSAMEG00000018434	GATB	glutamyl-tRNA amidotransferase subunit B
Heart	ENSAMEG00000018467	SRPK3	SRSF protein kinase 3
Heart	ENSAMEG00000018531	RAB9B	RAB9B, member RAS oncogene family
Heart	ENSAMEG00000018576	HSPB3	heat shock protein family B (small) member 3
Heart	ENSAMEG00000018579	LOC100467187	histone H2A type 1-E
Heart	ENSAMEG00000018627	--	--
Heart	ENSAMEG00000018695	C10orf71	chromosome 10 open reading frame 71
Heart	ENSAMEG00000018705	DIRAS1	DIRAS family GTPase 1
Heart	ENSAMEG00000018725	KCNE1	potassium voltage-gated channel subfamily E regulatory subunit 1
Heart	ENSAMEG00000018771	SLITRK6	SLIT and NTRK like family member 6
Heart	ENSAMEG00000018801	--	--
Heart	ENSAMEG00000018819	C1orf210	chromosome 1 open reading frame 210
Heart	ENSAMEG00000018822	TMEM125	transmembrane protein 125
Heart	ENSAMEG00000018830	GREM2	gremlin 2, DAN family BMP antagonist
Heart	ENSAMEG00000018834	MYADML2	myeloid associated differentiation marker like 2
Heart	ENSAMEG00000018844	TMEM37	transmembrane protein 37
Heart	ENSAMEG00000018850	PLN	phospholamban
Heart	ENSAMEG00000018994	--	--
Heart	ENSAMEG00000018995	UBXN10	UBX domain protein 10
Heart	ENSAMEG00000019008	--	--
Heart	ENSAMEG00000019019	PCDH9	protocadherin 9
Heart	ENSAMEG00000019029	GPR22	G protein-coupled receptor 22
Heart	ENSAMEG00000019176	--	--
Heart	ENSAMEG00000019267	KCNF1	potassium voltage-gated channel modifier subfamily F member 1
Heart	ENSAMEG00000019285	OXER1	oxoeicosanoid receptor 1
Heart	ENSAMEG00000019317	--	--
Heart	ENSAMEG00000019373	TMEM246	transmembrane protein 246

Heart	ENSAMEG00000019381	GPR182	G protein-coupled receptor 182
Heart	ENSAMEG00000019420	GJA3	gap junction protein alpha 3
Heart	ENSAMEG00000019470	LRRC10	leucine rich repeat containing 10
Heart	ENSAMEG00000019510	KCNJ11	potassium voltage-gated channel subfamily J member 11
Heart	ENSAMEG00000019531	TMEM200A	transmembrane protein 200A
Heart	ENSAMEG00000019547	KCNJ2	potassium voltage-gated channel subfamily J member 2
Heart	ENSAMEG00000019608	LOC100480801	hydroxycarboxylic acid receptor 2
Heart	ENSAMEG00000019692	GJA1	gap junction protein alpha 1
Heart	ENSAMEG00000019705	RNASE12	ribonuclease A family member 12 (inactive)
Heart	ENSAMEG00000019733	CHRM2	cholinergic receptor muscarinic 2
Heart	ENSAMEG00000019745	LOC100482289	histone H4
Heart	ENSAMEG00000019789	ACKR3	atypical chemokine receptor 3
Heart	ENSAMEG00000019910	GCNT2	glucosaminyl (N-acetyl) transferase 2, I-branching enzyme (I blood group)
Heart	ENSAMEG00000019975	XIRP1	xin actin binding repeat containing 1
Heart	ENSAMEG00000020121	TIGD6	tigger transposable element derived 6
Heart	Novel00001	--	hypothetical protein M91_07955
Heart	Novel00008	--	--
Heart	Novel00012	--	--
Heart	Novel00039	--	--
Heart	Novel00064	--	PREDICTED: alpha-1B adrenergic receptor
Heart	Novel00082	--	hypothetical protein PANDA_000426, partial
Heart	Novel00089	--	hCG2041078, partial
Heart	Novel00098	--	--
Heart	Novel00099	--	PREDICTED: heart- and neural crest derivatives-expressed protein 2 isoform X1
Heart	Novel00100	--	PREDICTED: uncharacterized protein LOC102163811
Heart	Novel00107	--	hypothetical protein M91_09901
Heart	Novel00149	--	--
Heart	Novel00155	--	--
Heart	Novel00164	--	--
Heart	Novel00165	--	--
Heart	Novel00215	--	--
Heart	Novel00288	--	PREDICTED: myosin light chain kinase 3 isoform X1
Heart	Novel00311	--	unnamed protein product
Heart	Novel00312	--	endonuclease/reverse transcriptase
Heart	Novel00320	--	--
Heart	Novel00326	--	unnamed protein product
Heart	Novel00327	--	--
Heart	Novel00328	--	--
Heart	Novel00381	--	--
Heart	Novel00382	--	--

Heart	Novel00396	--	PREDICTED: LOW QUALITY PROTEIN: dnaJ homolog subfamily C member 5G
Heart	Novel00404	--	--
Heart	Novel00417	--	PREDICTED: Kv channel-interacting protein 2 isoform X7
Heart	Novel00475	--	unnamed protein product
Heart	Novel00476	--	--
Heart	Novel00499	--	--
Heart	Novel00502	--	--
Heart	Novel00503	--	--
Heart	Novel00509	--	--
Heart	Novel00553	--	--
Heart	Novel00569	--	PREDICTED: uncharacterized protein C6orf106 homolog isoform X2
Heart	Novel00599	--	endonuclease/reverse transcriptase
Heart	Novel00658	--	PREDICTED: FYN-binding protein-like
Heart	Novel00659	--	--
Heart	Novel00692	--	unknown protein, partial
Heart	Novel00721	--	--
Heart	Novel00787	--	hypothetical protein M91_20110
Heart	Novel00790	--	PREDICTED: 60S ribosomal protein L39-like
Heart	Novel00791	--	hypothetical protein M91_11778
Heart	Novel00830	--	--
Heart	Novel00839	--	--
Heart	Novel00845	--	--
Heart	Novel00851	--	hypothetical protein EGM_19048, partial
Heart	Novel00868	--	--
Heart	Novel00910	--	PREDICTED: LOW QUALITY PROTEIN: melanopsin
Heart	Novel00917	--	hypothetical protein PANDA_003430
Heart	Novel00944	--	PREDICTED: Z-DNA-binding protein 1 isoform X5
Heart	Novel00977	--	Ac1262
Heart	Novel00987	--	--
Heart	Novel01035	--	PREDICTED: N-acetyllactosaminide beta-1,6-N-acetylglucosaminyl-transferase, isoform A-like
Heart	Novel01040	--	PREDICTED: N-acetyllactosaminide beta-1,6-N-acetylglucosaminyl-transferase, isoform A-like
Heart	Novel01048	--	--
Heart	Novel01060	--	--
Heart	Novel01116	--	PREDICTED: LOW QUALITY PROTEIN: ovochymase-2
Heart	Novel01131	--	PREDICTED: uncharacterized protein LOC103013146
Heart	Novel01157	--	--
Heart	Novel01158	--	--
Heart	Novel01183	--	Unknown (protein for MGC:16614)
Heart	Novel01257	--	--

Heart	Novel01292	--	--
Heart	Novel01299	--	--
Heart	Novel01370	--	--
Heart	Novel01385	--	endonuclease/reverse transcriptase
Heart	Novel01427	--	--
Heart	Novel01465	--	--
Heart	Novel01468	--	--
Heart	Novel01503	--	--
Heart	Novel01521	--	--
Heart	Novel01527	--	--
Heart	Novel01567	--	--
Heart	Novel01572	--	--
Heart	Novel01613	--	endonuclease/reverse transcriptase
Heart	Novel01624	--	--
Heart	Novel01653	--	--
Heart	Novel01657	--	hypothetical protein M91_02115
Heart	Novel01679	--	--
Heart	Novel01681	--	--
Heart	Novel01685	--	hypothetical protein
Heart	Novel01694	--	hypothetical protein PANDA_008428, partial
Heart	Novel01703	--	PREDICTED: cAMP-specific 3',5'-cyclic phosphodiesterase 4D-like
Heart	Novel01712	--	PREDICTED: killer cell lectin-like receptor 2
Heart	Novel01728	--	Matrix-remodeling-associated protein 7
Heart	Novel01732	--	--
Heart	Novel01745	--	--
Heart	Novel01763	--	--
Heart	Novel01855	--	--
Heart	Novel01857	--	--
Heart	Novel01858	--	PREDICTED: sodium/calcium exchanger 1 isoform X5
Heart	Novel01978	--	--
Heart	Novel01981	--	--
Heart	Novel02015	--	PREDICTED: LOW QUALITY PROTEIN: endogenous retrovirus group K member 113 Pol protein-like
Heart	Novel02017	--	--
Heart	Novel02025	--	--
Heart	Novel02032	--	--
Heart	Novel02116	--	--
Heart	Novel02121	--	hypothetical protein EGM_19048, partial
Heart	Novel02131	--	hypothetical protein
Heart	Novel02210	--	--
Heart	Novel02257	--	PREDICTED: regulator of G-protein signaling 6 isoform X3
Heart	Novel02274	--	--

Heart	Novel02286	--	PREDICTED: dapper homolog 3-like
Heart	Novel02318	--	PREDICTED: neuropilin-2 isoform X5
Heart	Novel02323	--	--
Heart	Novel02328	--	hypothetical protein M91_12176, partial
Heart	Novel02355	--	--
Heart	Novel02361	--	--
Heart	Novel02497	--	--
Heart	Novel02498	--	--
Heart	Novel02530	--	--
Heart	Novel02541	--	hypothetical protein PANDA_010201, partial
Heart	Novel02611	--	--
Heart	Novel02646	--	PREDICTED: LOW QUALITY PROTEIN: protein phosphatase 1 regulatory subunit 3A
Heart	Novel02648	--	hypothetical protein M91_14620
Heart	Novel02662	--	--
Heart	Novel02675	--	--
Heart	Novel02697	--	RecName: Full=LINE-1 reverse transcriptase homolog
Heart	Novel02703	--	Retrovirus-related Pol polyprotein LINE-1
Heart	Novel02741	--	--
Heart	Novel02825	--	--
Heart	Novel02846	--	unnamed protein product
Heart	Novel02866	--	--
Heart	Novel02887	--	--
Heart	Novel02905	--	PREDICTED: glypican-5
Heart	Novel02936	--	PREDICTED: LOW QUALITY PROTEIN: UPF0378 protein KIAA0100 homolog, partial
Heart	Novel02950	--	--
Heart	Novel02979	--	hypothetical protein M91_18376, partial
Heart	Novel03003	--	unnamed protein product
Heart	Novel03004	--	hypothetical protein M91_02115
Heart	Novel03005	--	hypothetical protein M91_18376, partial
Heart	Novel03006	--	putative p150
Heart	Novel03007	--	--
Heart	Novel03042	--	--
Heart	Novel03044	--	PREDICTED: LOW QUALITY PROTEIN: muscular LMNA-interacting protein
Heart	Novel03102	--	--
Heart	Novel03127	--	PREDICTED: roundabout homolog 2-like
Heart	Novel03159	--	hypothetical protein H920_06064
Heart	Novel03283	--	--
Heart	Novel03335	--	--
Heart	Novel03379	--	unnamed protein product
Heart	Novel03387	--	--

Heart	Novel03388	--	--
Heart	Novel03410	--	--
Heart	Novel03441	--	Retrovirus-related Pol polyprotein LINE-1
Heart	Novel03453	--	PREDICTED: 40S ribosomal protein SA-like
Heart	Novel03517	--	--
Heart	Novel03525	--	--
Heart	Novel03531	--	PREDICTED: FH1/FH2 domain-containing protein 3 isoform X9
Heart	Novel03537	--	--
Heart	Novel03598	--	--
Heart	Novel03631	--	--
Heart	Novel03636	--	--
Heart	Novel03690	--	--
Heart	Novel03699	--	--
Heart	Novel03701	--	--
Heart	Novel03702	--	--
Heart	Novel03713	--	PREDICTED: alpha-parvin isoform X3
Heart	Novel03715	--	--
Heart	Novel03716	--	hypothetical protein MDA_GLEAN10024817
Heart	Novel03736	--	PREDICTED: transcription factor 15, partial
Heart	Novel03757	--	--
Heart	Novel03790	--	Ribosomal protein S6
Heart	Novel03799	--	PREDICTED: zinc finger and BTB domain-containing protein 47
Heart	Novel03841	--	PREDICTED: cadherin-8 isoform X2
Heart	Novel03843	--	hypothetical protein PANDA_015316, partial
Heart	Novel03858	--	--
Heart	Novel03878	--	PREDICTED: integrin alpha-D
Heart	Novel03885	--	--
Heart	Novel03898	--	hypothetical protein M91_09901
Heart	Novel03902	--	hypothetical protein PANDA_015499
Heart	Novel03918	--	--
Heart	Novel03935	--	--
Heart	Novel03966	--	PREDICTED: uncharacterized protein LOC103679605
Heart	Novel04009	--	hypothetical protein
Heart	Novel04059	--	PREDICTED: glypican-5
Heart	Novel04089	--	--
Heart	Novel04095	--	PREDICTED: LOW QUALITY PROTEIN: cGMP-inhibited 3',5'-cyclic phosphodiesterase A
Heart	Novel04170	--	--
Heart	Novel04208	--	putative p150
Heart	Novel04222	--	--
Heart	Novel04231	--	--

Heart	Novel04233	--	--
Heart	Novel04303	--	--
Heart	Novel04305	--	--
Heart	Novel04306	--	--
Heart	Novel04373	--	hypothetical protein M91_15722
Heart	Novel04400	--	PREDICTED: catenin alpha-3-like, partial
Heart	Novel04454	--	--
Heart	Novel04457	--	--
Heart	Novel04482	--	--
Heart	Novel04539	--	hypothetical protein M91_00785, partial
Heart	Novel04540	--	hypothetical protein M91_14419, partial
Heart	Novel04561	--	PREDICTED: radial spoke head 1 homolog isoform X1
Heart	Novel04577	--	--
Heart	Novel04582	--	--
Heart	Novel04589	--	--
Heart	Novel04594	--	--
Heart	Novel04598	--	--
Heart	Novel04662	--	--
Heart	Novel04682	--	--
Heart	Novel04711	--	PREDICTED: histone H2A type 1-J isoform X1
Heart	Novel04714	--	--
Heart	Novel04716	--	unnamed protein product
Heart	Novel04730	--	PREDICTED: receptor expression-enhancing protein 1 isoform X2
Heart	Novel04731	--	PREDICTED: catenin alpha-3-like, partial
Heart	Novel04753	--	Da1-12
Heart	Novel04794	--	--
Heart	Novel04795	--	--
Heart	Novel04806	--	--
Heart	Novel04837	--	--
Heart	Novel04846	--	--
Heart	Novel04855	--	PREDICTED: melanoma inhibitory activity protein 3-like
Heart	Novel04874	--	tRNA-splicing endonuclease subunit Sen15
Heart	Novel04895	--	--
Heart	Novel04910	--	--
Heart	Novel04930	--	--
Heart	Novel04934	--	unnamed protein product
Heart	Novel04977	--	unnamed protein product
Heart	Novel05015	--	--
Heart	Novel05017	--	--
Heart	Novel05024	--	--
Heart	Novel05047	--	--
Heart	Novel05093	--	--

Heart	Novel05099	--	PREDICTED: RNA binding protein fox-1 homolog 2 isoform X8
Heart	Novel05103	--	--
Heart	Novel05122	--	--
Heart	Novel05127	--	PREDICTED: uncharacterized protein LOC101368442
Heart	Novel05162	--	PREDICTED: uncharacterized protein LOC102156706
Heart	Novel05204	--	PREDICTED: RNA binding protein fox-1 homolog 1
Heart	Novel05205	--	endonuclease/reverse transcriptase
Heart	Novel05219	--	LINE-1 element ORF2 (predicted)
Heart	Novel05249	--	PREDICTED: LOW QUALITY PROTEIN: obscurin-like
Heart	Novel05250	--	PREDICTED: LOW QUALITY PROTEIN: obscurin-like
Heart	Novel05256	--	hypothetical protein CB1_000794010
Heart	Novel05283	--	--
Heart	Novel05294	--	--
Heart	Novel05299	--	--
Heart	Novel05304	--	--
Heart	Novel05319	--	PREDICTED: putative uncharacterized protein C14orf132 homolog
Heart	Novel05328	--	--
Heart	Novel05341	--	--
Heart	Novel05361	--	--
Heart	Novel05378	--	--
Heart	Novel05379	--	--
Heart	Novel05417	--	--
Heart	Novel05490	--	--
Heart	Novel05516	--	--
Heart	Novel05641	--	--
Heart	Novel05684	--	--
Heart	Novel05783	--	--
Heart	Novel05813	--	--
Heart	Novel05817	--	PREDICTED: interferon-inducible GTPase 1-like
Heart	Novel05824	--	--
Heart	Novel05839	--	unnamed protein product
Heart	Novel05866	--	--
Heart	Novel05911	--	--
Heart	Novel05929	--	tetratricopeptide repeat protein 39B-like protein, partial
Heart	Novel05940	--	slow myosin heavy chain
Heart	Novel05971	--	--
Heart	Novel06017	--	PREDICTED: epidermal growth factor receptor isoform X2
Liver	ENSAMEG00000000008	SLC13A3	solute carrier family 13 member 3
Liver	ENSAMEG00000000041	SYT11	synaptotagmin 11
Liver	ENSAMEG00000000094	JAG2	jagged 2
Liver	ENSAMEG00000000107	HOGA1	4-hydroxy-2-oxoglutarate aldolase 1

Liver	ENSAMEG00000000141	CLU	clusterin
Liver	ENSAMEG00000000147	LOC100478547	L-gulonolactone oxidase
Liver	ENSAMEG00000000189	SCN1B	sodium voltage-gated channel beta subunit 1
Liver	ENSAMEG00000000244	HAMP	hepcidin antimicrobial peptide
Liver	ENSAMEG00000000245	MAG	myelin associated glycoprotein
Liver	ENSAMEG00000000261	TF	transferrin
Liver	ENSAMEG00000000315	RAPGEF4	Rap guanine nucleotide exchange factor 4
Liver	ENSAMEG00000000316	TRHDE	thyrotropin releasing hormone degrading enzyme
Liver	ENSAMEG00000000317	--	--
Liver	ENSAMEG00000000341	LOC100468106	inhibitor of carbonic anhydrase
Liver	ENSAMEG00000000342	CYP2J2	cytochrome P450 family 2 subfamily J member 2
Liver	ENSAMEG00000000398	COLEC10	collectin subfamily member 10
Liver	ENSAMEG00000000438	MS4A4A	membrane spanning 4-domains A4A
Liver	ENSAMEG00000000451	--	--
Liver	ENSAMEG00000000470	DTNA	dystrobrevin alpha
Liver	ENSAMEG00000000656	--	--
Liver	ENSAMEG00000000671	PLPPR1	phospholipid phosphatase related 1
Liver	ENSAMEG00000000677	FOXA3	forkhead box A3
Liver	ENSAMEG00000000923	ABCB4	ATP binding cassette subfamily B member 4
Liver	ENSAMEG00000000931	FHOD3	formin homology 2 domain containing 3
Liver	ENSAMEG00000000982	GOLT1A	golgi transport 1A
Liver	ENSAMEG00000000994	KNG1	kininogen 1
Liver	ENSAMEG00000001013	HRG	histidine rich glycoprotein
Liver	ENSAMEG00000001033	MTTP	microsomal triglyceride transfer protein
Liver	ENSAMEG00000001036	FETUB	fetuin B
Liver	ENSAMEG00000001041	AHSG	alpha 2-HS glycoprotein
Liver	ENSAMEG00000001045	C5	complement C5
Liver	ENSAMEG00000001179	SLC30A1	solute carrier family 30 member 1
Liver	ENSAMEG00000001253	GCKR	glucokinase regulator
Liver	ENSAMEG00000001290	STEAP4	STEAP4 metalloreductase
Liver	ENSAMEG00000001367	SPINK8	serine peptidase inhibitor, Kazal type 8 (putative)
Liver	ENSAMEG00000001428	FGF7	fibroblast growth factor 7
Liver	ENSAMEG00000001439	LOC100465875	zinc finger protein 671
Liver	ENSAMEG00000001451	FTCD	formimidoyltransferase cyclodeaminase
Liver	ENSAMEG00000001528	MFSD4A	major facilitator superfamily domain containing 4A
Liver	ENSAMEG00000001588	FGFBP1	fibroblast growth factor binding protein 1
Liver	ENSAMEG00000001662	TBX20	T-box 20
Liver	ENSAMEG00000001767	--	--
Liver	ENSAMEG00000001793	ADK	adenosine kinase
Liver	ENSAMEG00000001835	WASF3	WAS protein family member 3
Liver	ENSAMEG00000001848	PDE9A	phosphodiesterase 9A
Liver	ENSAMEG00000001851	--	--
Liver	ENSAMEG00000001910	GLT1D1	glycosyltransferase 1 domain containing 1

Liver	ENSAMEG00000001937	--	--
Liver	ENSAMEG00000001939	BRSK2	BR serine/threonine kinase 2
Liver	ENSAMEG00000001970	PLCXD1	phosphatidylinositol specific phospholipase C X domain containing 1
Liver	ENSAMEG00000001986	FGF19	fibroblast growth factor 19
Liver	ENSAMEG00000002007	SLC24A5	solute carrier family 24 member 5
Liver	ENSAMEG00000002096	ADAMTS8	ADAM metallopeptidase with thrombospondin type 1 motif 8
Liver	ENSAMEG00000002098	HAL	histidine ammonia-lyase
Liver	ENSAMEG00000002115	AMDHD1	amidohydrolase domain containing 1
Liver	ENSAMEG00000002167	IGF2	insulin like growth factor 2
Liver	ENSAMEG00000002176	INS-IGF2	INS-IGF2 readthrough
Liver	ENSAMEG00000002199	CREB3L3	cAMP responsive element binding protein 3 like 3
Liver	ENSAMEG00000002202	CLDN19	claudin 19
Liver	ENSAMEG00000002213	PGLYRP2	peptidoglycan recognition protein 2
Liver	ENSAMEG00000002287	LOC100482523	haptoglobin
Liver	ENSAMEG00000002316	LOC109488752	cytochrome P450 3A12-like
Liver	ENSAMEG00000002320	LOC100471068	glutathione S-transferase A1-like
Liver	ENSAMEG00000002374	TAT	tyrosine aminotransferase
Liver	ENSAMEG00000002383	PROS1	protein S
Liver	ENSAMEG00000002399	--	--
Liver	ENSAMEG00000002423	CCDC198	coiled-coil domain containing 198
Liver	ENSAMEG00000002450	PLCB4	phospholipase C beta 4
Liver	ENSAMEG00000002508	TMEM45B	transmembrane protein 45B
Liver	ENSAMEG00000002540	--	--
Liver	ENSAMEG00000002548	DHODH	dihydroorotate dehydrogenase (quinone)
Liver	ENSAMEG00000002691	ELOVL2	ELOVL fatty acid elongase 2
Liver	ENSAMEG00000002751	RIPPLY1	ripply transcriptional repressor 1
Liver	ENSAMEG00000002772	SLC51B	solute carrier family 51 beta subunit
Liver	ENSAMEG00000002950	ADAM23	ADAM metallopeptidase domain 23
Liver	ENSAMEG00000002954	MLLT3	MLLT3, super elongation complex subunit
Liver	ENSAMEG00000002998	VIL1	villin 1
Liver	ENSAMEG00000003019	TMEM30B	transmembrane protein 30B
Liver	ENSAMEG00000003024	DPYSL3	dihydropyrimidinase like 3
Liver	ENSAMEG00000003097	LIPC	lipase C, hepatic type
Liver	ENSAMEG00000003107	LOC100468534	cytochrome P450 2C23-like
Liver	ENSAMEG00000003132	CYP2E1	cytochrome P450 family 2 subfamily E member 1
Liver	ENSAMEG00000003162	OR13G1	olfactory receptor 13G1
Liver	ENSAMEG00000003167	SCART1	scavenger receptor family member expressed on T cells 1
Liver	ENSAMEG00000003191	PFKP	phosphofructokinase, platelet
Liver	ENSAMEG00000003262	PRAP1	proline rich acidic protein 1
Liver	ENSAMEG00000003353	LCN9	lipocalin 9
Liver	ENSAMEG00000003367	--	--

Liver	ENSAMEG00000003375	--	--
Liver	ENSAMEG00000003470	SLC22A16	solute carrier family 22 member 16
Liver	ENSAMEG00000003480	C4BPA	complement component 4 binding protein alpha
Liver	ENSAMEG00000003490	PECR	peroxisomal trans-2-enoyl-CoA reductase
Liver	ENSAMEG00000003516	C4BPB	complement component 4 binding protein beta
Liver	ENSAMEG00000003517	FN1	fibronectin 1
Liver	ENSAMEG00000003557	IL13RA2	interleukin 13 receptor subunit alpha 2
Liver	ENSAMEG00000003564	FNDC1	fibronectin type III domain containing 1
Liver	ENSAMEG00000003566	TMEM86B	transmembrane protein 86B
Liver	ENSAMEG00000003628	C1orf116	chromosome 1 open reading frame 116
Liver	ENSAMEG00000003674	SERPINA7	serpin family A member 7
Liver	ENSAMEG00000003716	--	--
Liver	ENSAMEG00000003736	ACE	angiotensin I converting enzyme
Liver	ENSAMEG00000003852	CYB561	cytochrome b561
Liver	ENSAMEG00000003871	PTGER3	prostaglandin E receptor 3
Liver	ENSAMEG00000003941	APOF	apolipoprotein F
Liver	ENSAMEG00000004006	SLC15A1	solute carrier family 15 member 1
Liver	ENSAMEG00000004014	GLS2	glutaminase 2
Liver	ENSAMEG00000004046	--	--
Liver	ENSAMEG00000004055	C8A	complement C8 alpha chain
Liver	ENSAMEG00000004085	CORO6	coronin 6
Liver	ENSAMEG00000004223	PLXDC1	plexin domain containing 1
Liver	ENSAMEG00000004329	ABCC11	ATP binding cassette subfamily C member 11
Liver	ENSAMEG00000004376	LOC105235077	cytochrome P450 3A12-like
Liver	ENSAMEG00000004402	LOC100464453	contactin-associated protein-like 2
Liver	ENSAMEG00000004409	B4GALNT3	beta-1,4-N-acetyl-galactosaminyltransferase 3
Liver	ENSAMEG00000004584	SLC6A12	solute carrier family 6 member 12
Liver	ENSAMEG00000004675	SHMT1	serine hydroxymethyltransferase 1
Liver	ENSAMEG00000004701	SORT1	sortilin 1
Liver	ENSAMEG00000004767	HGD	homogentisate 1,2-dioxygenase
Liver	ENSAMEG00000004847	AFM	afamin
Liver	ENSAMEG00000004873	INHBC	inhibin beta C subunit
Liver	ENSAMEG00000004875	INHBE	inhibin beta E subunit
Liver	ENSAMEG00000004925	ZNF548	zinc finger protein 548
Liver	ENSAMEG00000005051	VTN	vitronectin
Liver	ENSAMEG00000005080	CPS1	carbamoyl-phosphate synthase 1
Liver	ENSAMEG00000005085	F11	coagulation factor XI
Liver	ENSAMEG00000005093	SLC12A2	solute carrier family 12 member 2
Liver	ENSAMEG00000005097	KLKB1	kallikrein B1
Liver	ENSAMEG00000005176	RASGEF1B	RasGEF domain family member 1B
Liver	ENSAMEG00000005288	--	--
Liver	ENSAMEG00000005374	DSTN	destrin, actin depolymerizing factor
Liver	ENSAMEG00000005464	PFKFB1	6-phosphofructo-2-kinase/fructose-2,6-bisphosphatase 1

Liver	ENSAMEG00000005568	SYNM	synemin
Liver	ENSAMEG00000005596	MSMO1	methylsterol monooxygenase 1
Liver	ENSAMEG00000005651	AQP11	aquaporin 11
Liver	ENSAMEG00000005664	SLCO3A1	solute carrier organic anion transporter family member 3A1
Liver	ENSAMEG00000005696	BHMT	betaine--homocysteine S-methyltransferase
Liver	ENSAMEG00000005723	ESRP1	epithelial splicing regulatory protein 1
Liver	ENSAMEG00000005730	LOC100466123	UDP-glucuronosyltransferase 2B31
Liver	ENSAMEG00000005769	ABCG5	ATP binding cassette subfamily G member 5
Liver	ENSAMEG00000005817	SLC22A9	solute carrier family 22 member 9
Liver	ENSAMEG00000005956	SLC22A10	solute carrier family 22 member 10
Liver	ENSAMEG00000005960	TMTC1	transmembrane and tetratricopeptide repeat containing 1
Liver	ENSAMEG00000006059	TREH	trehalase
Liver	ENSAMEG00000006080	GC	GC, vitamin D binding protein
Liver	ENSAMEG00000006108	--	--
Liver	ENSAMEG00000006218	LOC100480295	multidrug resistance-associated protein 9
Liver	ENSAMEG00000006243	LOC100471407	cytochrome P450 2C41
Liver	ENSAMEG00000006300	SERPINA6	serpin family A member 6
Liver	ENSAMEG00000006318	SERPINA1	serpin family A member 1
Liver	ENSAMEG00000006330	--	--
Liver	ENSAMEG00000006332	ARMC3	armadillo repeat containing 3
Liver	ENSAMEG00000006363	SERPINA11	serpin family A member 11
Liver	ENSAMEG00000006400	SDK1	sidekick cell adhesion molecule 1
Liver	ENSAMEG00000006404	PAH	phenylalanine hydroxylase
Liver	ENSAMEG00000006405	SERPINA4	serpin family A member 4
Liver	ENSAMEG00000006411	SERPINA5	serpin family A member 5
Liver	ENSAMEG00000006430	SERPINA3	serpin family A member 3
Liver	ENSAMEG00000006448	UROC1	urocanate hydratase 1
Liver	ENSAMEG00000006475	SULT1C3	sulfotransferase 1C1
Liver	ENSAMEG00000006497	EGFLAM	EGF like, fibronectin type III and laminin G domains
Liver	ENSAMEG00000006694	PNPLA3	patatin like phospholipase domain containing 3
Liver	ENSAMEG00000006737	UCK2	uridine-cytidine kinase 2
Liver	ENSAMEG00000006748	SLC41A2	solute carrier family 41 member 2
Liver	ENSAMEG00000006830	LOC105234899	anosmin-1
Liver	ENSAMEG00000006884	SLC38A4	solute carrier family 38 member 4
Liver	ENSAMEG00000006910	F2	coagulation factor II, thrombin
Liver	ENSAMEG00000006922	ERLIN1	ER lipid raft associated 1
Liver	ENSAMEG00000006944	--	--
Liver	ENSAMEG00000006951	CPN1	carboxypeptidase N subunit 1
Liver	ENSAMEG00000006981	FAM189A2	family with sequence similarity 189 member A2
Liver	ENSAMEG00000007016	C8B	complement C8 beta chain
Liver	ENSAMEG00000007018	ABCC2	ATP binding cassette subfamily C member 2
Liver	ENSAMEG00000007061	FKBP11	FK506 binding protein 11

Liver	ENSAMEG00000007080	SLC13A5	solute carrier family 13 member 5
Liver	ENSAMEG00000007121	OTC	ornithine carbamoyltransferase
Liver	ENSAMEG00000007161	C3	complement C3
Liver	ENSAMEG00000007319	MS4A15	membrane spanning 4-domains A15
Liver	ENSAMEG00000007338	LOC100478101	lysozyme C, milk isozyme
Liver	ENSAMEG00000007391	SYT9	synaptotagmin 9
Liver	ENSAMEG00000007398	SLC35E4	solute carrier family 35 member E4
Liver	ENSAMEG00000007407	A1BG	alpha-1-B glycoprotein
Liver	ENSAMEG00000007412	RAP1GAP2	RAP1 GTPase activating protein 2
Liver	ENSAMEG00000007428	ONECUT1	one cut homeobox 1
Liver	ENSAMEG00000007460	SLC27A5	solute carrier family 27 member 5
Liver	ENSAMEG00000007512	HSD3B7	hydroxy-delta-5-steroid dehydrogenase, 3 beta- and steroid delta-isomerase 7
Liver	ENSAMEG00000007532	TMED6	transmembrane p24 trafficking protein 6
Liver	ENSAMEG00000007549	CACNA1H	calcium voltage-gated channel subunit alpha1 H
Liver	ENSAMEG00000007593	--	--
Liver	ENSAMEG00000007612	PRICKLE2	prickle planar cell polarity protein 2
Liver	ENSAMEG00000007809	SERPINF1	serpin family F member 1
Liver	ENSAMEG00000007834	SLC1A2	solute carrier family 1 member 2
Liver	ENSAMEG00000007848	WFIKKN2	WAP, follistatin/kazal, immunoglobulin, kunitz and netrin domain containing 2
Liver	ENSAMEG00000007857	SERPINF2	serpin family F member 2
Liver	ENSAMEG00000008014	POU2F3	POU class 2 homeobox 3
Liver	ENSAMEG00000008089	PIP5K1B	phosphatidylinositol-4-phosphate 5-kinase type 1 beta
Liver	ENSAMEG00000008108	ITIH4	inter-alpha-trypsin inhibitor heavy chain family member 4
Liver	ENSAMEG00000008119	TUSC3	tumor suppressor candidate 3
Liver	ENSAMEG00000008201	ITIH3	inter-alpha-trypsin inhibitor heavy chain 3
Liver	ENSAMEG00000008282	ITIH1	inter-alpha-trypsin inhibitor heavy chain 1
Liver	ENSAMEG00000008373	MBOAT2	membrane bound O-acyltransferase domain containing 2
Liver	ENSAMEG00000008599	PKHD1	PKHD1 ciliary IPT domain containing fibrocystin/polyductin
Liver	ENSAMEG00000008612	SDSL	serine dehydratase like
Liver	ENSAMEG00000008632	SDS	serine dehydratase
Liver	ENSAMEG00000008662	APOA1	Apolipoprotein A-I Proapolipoprotein A-I Truncated apolipoprotein A-I
Liver	ENSAMEG00000008670	APOC3	apolipoprotein C3
Liver	ENSAMEG00000008780	SERPIND1	serpin family D member 1
Liver	ENSAMEG00000008782	SEMA3G	semaphorin 3G
Liver	ENSAMEG00000008818	RASAL1	RAS protein activator like 1
Liver	ENSAMEG00000008842	CYP1A2	cytochrome P450 family 1 subfamily A member 2
Liver	ENSAMEG00000008863	ACSM2B	acyl-CoA synthetase medium chain family member 2B
Liver	ENSAMEG00000009122	RHOU	ras homolog family member U
Liver	ENSAMEG00000009303	MYH11	myosin heavy chain 11
Liver	ENSAMEG00000009385	--	--

Liver	ENSAMEG00000009436	LOC100474489	uncharacterized LOC100474489
Liver	ENSAMEG00000009440	LOC100472452	cytochrome P450 2D15
Liver	ENSAMEG00000009485	MASP2	mannan binding lectin serine peptidase 2
Liver	ENSAMEG00000009516	--	--
Liver	ENSAMEG00000009545	GALP	galanin like peptide
Liver	ENSAMEG00000009551	--	--
Liver	ENSAMEG00000009713	AGT	angiotensinogen
Liver	ENSAMEG00000009798	PROM2	prominin 2
Liver	ENSAMEG00000009854	FAM151A	family with sequence similarity 151 member A
Liver	ENSAMEG00000009861	SLC17A3	solute carrier family 17 member 3
Liver	ENSAMEG00000009877	CIB3	calcium and integrin binding family member 3
Liver	ENSAMEG00000009948	SLC17A4	solute carrier family 17 member 4
Liver	ENSAMEG00000009952	DHCR24	24-dehydrocholesterol reductase
Liver	ENSAMEG00000009962	ITIH2	inter-alpha-trypsin inhibitor heavy chain 2
Liver	ENSAMEG00000009978	SLC30A10	solute carrier family 30 member 10
Liver	ENSAMEG00000010170	TRIM9	tripartite motif containing 9
Liver	ENSAMEG00000010217	FOXJ1	forkhead box J1
Liver	ENSAMEG00000010227	ARG1	arginase 1
Liver	ENSAMEG00000010248	F10	coagulation factor X
Liver	ENSAMEG00000010256	PON3	paraoxonase 3
Liver	ENSAMEG00000010261	ALDH5A1	aldehyde dehydrogenase 5 family member A1
Liver	ENSAMEG00000010289	F7	coagulation factor VII
Liver	ENSAMEG00000010291	SPART	spartin
Liver	ENSAMEG00000010294	GPLD1	glycosylphosphatidylinositol specific phospholipase D1
Liver	ENSAMEG00000010324	ENPP3	ectonucleotide pyrophosphatase/phosphodiesterase 3
Liver	ENSAMEG00000010395	TM4SF4	transmembrane 4 L six family member 4
Liver	ENSAMEG00000010411	BAIAP2L2	BAI1 associated protein 2 like 2
Liver	ENSAMEG00000010415	--	--
Liver	ENSAMEG00000010479	--	--
Liver	ENSAMEG00000010547	MOXD1	monooxygenase DBH like 1
Liver	ENSAMEG00000010616	VNN1	vanin 1
Liver	ENSAMEG00000010632	CDHR5	cadherin related family member 5
Liver	ENSAMEG00000010658	FAM20A	FAM20A, golgi associated secretory pathway pseudokinase
Liver	ENSAMEG00000010706	PCDHGB4	protocadherin gamma subfamily B, 4
Liver	ENSAMEG00000010784	ABCB11	ATP binding cassette subfamily B member 11
Liver	ENSAMEG00000010793	ANGPTL8	angiopoietin like 8
Liver	ENSAMEG00000010817	NR0B2	nuclear receptor subfamily 0 group B member 2
Liver	ENSAMEG00000011075	AVP	arginine vasopressin
Liver	ENSAMEG00000011089	LHX2	LIM homeobox 2
Liver	ENSAMEG00000011125	SLC25A47	solute carrier family 25 member 47
Liver	ENSAMEG00000011178	GDF15	growth differentiation factor 15
Liver	ENSAMEG00000011252	AZGP1	alpha-2-glycoprotein 1, zinc-binding
Liver	ENSAMEG00000011259	MBNL3	muscleblind like splicing regulator 3

Liver	ENSAMEG00000011289	CLTCL1	clathrin heavy chain like 1
Liver	ENSAMEG00000011329	PELI3	pellino E3 ubiquitin protein ligase family member 3
Liver	ENSAMEG00000011547	IL1RAP	interleukin 1 receptor accessory protein
Liver	ENSAMEG00000011605	ADAMTS13	ADAM metallopeptidase with thrombospondin type 1 motif 13
Liver	ENSAMEG00000011718	LOC100464961	UDP-glucuronosyltransferase 2B31
Liver	ENSAMEG00000011723	SLC25A15	solute carrier family 25 member 15
Liver	ENSAMEG00000011739	LOC100471847	phosphatidylinositol 3,4,5-trisphosphate 3-phosphatase TPTE2
Liver	ENSAMEG00000011749	LOC100464709	UDP-glucuronosyltransferase 2C1
Liver	ENSAMEG00000011842	APOH	apolipoprotein H
Liver	ENSAMEG00000011854	HEBP2	heme binding protein 2
Liver	ENSAMEG00000011870	SLC38A3	solute carrier family 38 member 3
Liver	ENSAMEG00000011881	AGMAT	agmatinase
Liver	ENSAMEG00000011898	CFH	complement factor H
Liver	ENSAMEG00000012004	TLN2	talin 2
Liver	ENSAMEG00000012016	ANGPTL6	angiopoietin like 6
Liver	ENSAMEG00000012033	LOC100470692	complement C3
Liver	ENSAMEG00000012066	ACOX2	acyl-CoA oxidase 2
Liver	ENSAMEG00000012078	LBP	lipopolysaccharide binding protein
Liver	ENSAMEG00000012096	UGT2A1	UDP glucuronosyltransferase family 2 member A1 complex locus
Liver	ENSAMEG00000012122	GLP2R	glucagon like peptide 2 receptor
Liver	ENSAMEG00000012141	SOAT2	sterol O-acyltransferase 2
Liver	ENSAMEG00000012216	LOC100468789	sulfotransferase 1 family member D1
Liver	ENSAMEG00000012233	VLDLR	very low density lipoprotein receptor
Liver	ENSAMEG00000012249	SLC6A20	solute carrier family 6 member 20
Liver	ENSAMEG00000012296	PDE7A	phosphodiesterase 7A
Liver	ENSAMEG00000012313	NADK2	NAD kinase 2, mitochondrial
Liver	ENSAMEG00000012319	LMCD1	LIM and cysteine rich domains 1
Liver	ENSAMEG00000012393	MST1	macrophage stimulating 1
Liver	ENSAMEG00000012397	LOC100477732	UDP-glucuronosyltransferase 3A2
Liver	ENSAMEG00000012408	DACH1	dachshund family transcription factor 1
Liver	ENSAMEG00000012477	RTP3	receptor transporter protein 3
Liver	ENSAMEG00000012541	VIPR1	vasoactive intestinal peptide receptor 1
Liver	ENSAMEG00000012553	--	--
Liver	ENSAMEG00000012556	LOC100474871	alpha-1-acid glycoprotein
Liver	ENSAMEG00000012650	NR5A2	nuclear receptor subfamily 5 group A member 2
Liver	ENSAMEG00000012730	APOC2	apolipoprotein C2
Liver	ENSAMEG00000012734	APOC4	apolipoprotein C4
Liver	ENSAMEG00000012737	APOC1	apolipoprotein C1
Liver	ENSAMEG00000012740	APOE	apolipoprotein E

Liver	ENSAMEG00000012783	MAGI2	membrane associated guanylate kinase, WW and PDZ domain containing 2
Liver	ENSAMEG00000012797	PRDX4	peroxiredoxin 4
Liver	ENSAMEG00000012834	KIF12	kinesin family member 12
Liver	ENSAMEG00000012851	AMBP	alpha-1-microglobulin/bikunin precursor
Liver	ENSAMEG00000012883	HAPLN3	hyaluronan and proteoglycan link protein 3
Liver	ENSAMEG00000012894	MFGE8	milk fat globule-EGF factor 8 protein
Liver	ENSAMEG00000013016	LOC100482856	pulmonary surfactant-associated protein A
Liver	ENSAMEG00000013025	MAT1A	methionine adenosyltransferase 1A
Liver	ENSAMEG00000013049	LIMCH1	LIM and calponin homology domains 1
Liver	ENSAMEG00000013084	--	--
Liver	ENSAMEG00000013086	APOB	apolipoprotein B
Liver	ENSAMEG00000013126	KYNU	kynureninase
Liver	ENSAMEG00000013144	CPB2	carboxypeptidase B2
Liver	ENSAMEG00000013281	NNMT	nicotinamide N-methyltransferase
Liver	ENSAMEG00000013288	LRAT	lecithin retinol acyltransferase
Liver	ENSAMEG00000013326	C8G	complement C8 gamma chain
Liver	ENSAMEG00000013516	OMD	osteomodulin
Liver	ENSAMEG00000013530	UCMA	upper zone of growth plate and cartilage matrix associated
Liver	ENSAMEG00000013550	--	--
Liver	ENSAMEG00000013562	ATF5	activating transcription factor 5
Liver	ENSAMEG00000013566	C1R	complement C1r
Liver	ENSAMEG00000013581	DNASE2B	deoxyribonuclease 2 beta
Liver	ENSAMEG00000013593	RAPGEF3	Rap guanine nucleotide exchange factor 3
Liver	ENSAMEG00000013668	C1S	complement C1s
Liver	ENSAMEG00000013690	SLC7A2	solute carrier family 7 member 2
Liver	ENSAMEG00000013784	LOC100480698	glutathione S-transferase theta-1
Liver	ENSAMEG00000013822	RBP4	retinol binding protein 4
Liver	ENSAMEG00000013869	A2M	alpha-2-macroglobulin
Liver	ENSAMEG00000013900	LOC100473316	alkylglycerol monooxygenase
Liver	ENSAMEG00000013958	SLC6A11	sodium- and chloride-dependent GABA transporter 3
Liver	ENSAMEG00000013968	MFSD6	major facilitator superfamily domain containing 6
Liver	ENSAMEG00000014005	TRIB3	tribbles pseudokinase 3
Liver	ENSAMEG00000014024	HSPA12A	heat shock protein family A (Hsp70) member 12A
Liver	ENSAMEG00000014093	CORO2B	coronin 2B
Liver	ENSAMEG00000014110	BMP10	bone morphogenetic protein 10
Liver	ENSAMEG00000014137	HSD17B2	hydroxysteroid 17-beta dehydrogenase 2
Liver	ENSAMEG00000014369	PARM1	prostate androgen-regulated mucin-like protein 1
Liver	ENSAMEG00000014373	NQO2	N-ribosyldihydronicotinamide:quinone reductase 2
Liver	ENSAMEG00000014441	SEC16B	SEC16 homolog B, endoplasmic reticulum export factor
Liver	ENSAMEG00000014444	PRG2	proteoglycan 2, pro eosinophil major basic protein
Liver	ENSAMEG00000014526	SERPING1	serpin family G member 1
Liver	ENSAMEG00000014693	NPY1R	neuropeptide Y receptor Y1

Liver	ENSAMEG00000014698	HSD17B14	hydroxysteroid 17-beta dehydrogenase 14
Liver	ENSAMEG00000014737	NTRK1	neurotrophic receptor tyrosine kinase 1
Liver	ENSAMEG00000014756	--	--
Liver	ENSAMEG00000014759	FGF21	fibroblast growth factor 21
Liver	ENSAMEG00000014791	--	--
Liver	ENSAMEG00000014806	ADCY1	adenylate cyclase 1
Liver	ENSAMEG00000014809	ONECUT2	one cut homeobox 2
Liver	ENSAMEG00000014815	HGFAC	HGF activator
Liver	ENSAMEG00000014822	RASL10B	RAS like family 10 member B
Liver	ENSAMEG00000014828	TFR2	transferrin receptor 2
Liver	ENSAMEG00000014850	ENPEP	glutamyl aminopeptidase
Liver	ENSAMEG00000014871	PRRT2	proline rich transmembrane protein 2
Liver	ENSAMEG00000014889	ATF7IP2	activating transcription factor 7 interacting protein 2
Liver	ENSAMEG00000014891	PCOLCE	procollagen C-endopeptidase enhancer
Liver	ENSAMEG00000014904	CCL16	C-C motif chemokine ligand 16
Liver	ENSAMEG00000014967	ANGPTL3	angiopoietin like 3
Liver	ENSAMEG00000015076	--	--
Liver	ENSAMEG00000015085	ROR1	receptor tyrosine kinase like orphan receptor 1
Liver	ENSAMEG00000015162	WEE2	Wee1-like protein kinase 2
Liver	ENSAMEG00000015180	GATA2	GATA binding protein 2
Liver	ENSAMEG00000015230	C6	complement C6
Liver	ENSAMEG00000015302	NRAP	nebulin related anchoring protein
Liver	ENSAMEG00000015349	HABP2	hyaluronan binding protein 2
Liver	ENSAMEG00000015360	FEZF2	FEZ family zinc finger 2
Liver	ENSAMEG00000015443	APOA2	apolipoprotein A2
Liver	ENSAMEG00000015468	TDO2	tryptophan 2,3-dioxygenase
Liver	ENSAMEG00000015595	--	--
Liver	ENSAMEG00000015644	SERPINC1	serpin family C member 1
Liver	ENSAMEG00000015680	CRP	C-reactive protein
Liver	ENSAMEG00000015688	APCS	amyloid P component, serum
Liver	ENSAMEG00000015845	LNX2	ligand of numb-protein X 2
Liver	ENSAMEG00000015866	ATP2B2	ATPase plasma membrane Ca2+ transporting 2
Liver	ENSAMEG00000015867	ACOT12	acyl-CoA thioesterase 12
Liver	ENSAMEG00000015875	SLC17A2	solute carrier family 17 member 2
Liver	ENSAMEG00000015924	EYA4	EYA transcriptional coactivator and phosphatase 4
Liver	ENSAMEG00000015960	PLG	plasminogen
Liver	ENSAMEG00000016027	HAO1	hydroxyacid oxidase 1
Liver	ENSAMEG00000016034	--	--
Liver	ENSAMEG00000016100	TM4SF5	transmembrane 4 L six family member 5
Liver	ENSAMEG00000016104	LOC100470213	cytochrome P450 2C21
Liver	ENSAMEG00000016164	UGT1A1	UDP glucuronosyltransferase family 1 member A1
Liver	ENSAMEG00000016189	F9	coagulation factor IX
Liver	ENSAMEG00000016198	GYS2	glycogen synthase 2

Liver	ENSAMEG00000016273	FREM2	FRAS1 related extracellular matrix protein 2
Liver	ENSAMEG00000016356	CLEC10A	C-type lectin domain containing 10A
Liver	ENSAMEG00000016372	HPX	hemopexin
Liver	ENSAMEG00000016377	ASGR2	asialoglycoprotein receptor 2
Liver	ENSAMEG00000016382	ASGR1	asialoglycoprotein receptor 1
Liver	ENSAMEG00000016434	--	--
Liver	ENSAMEG00000016440	--	--
Liver	ENSAMEG00000016453	DAO	D-amino acid oxidase
Liver	ENSAMEG00000016464	SPIC	Spi-C transcription factor
Liver	ENSAMEG00000016482	CNKSR2	connector enhancer of kinase suppressor of Ras 2
Liver	ENSAMEG00000016507	PCK2	phosphoenolpyruvate carboxykinase 2, mitochondrial
Liver	ENSAMEG00000016667	--	--
Liver	ENSAMEG00000016675	SNED1	sushi, nidogen and EGF like domains 1
Liver	ENSAMEG00000016798	PROC	protein C, inactivator of coagulation factors Va and VIIIa
Liver	ENSAMEG00000016952	CA10	carbonic anhydrase 10
Liver	ENSAMEG00000016994	THPO	thrombopoietin
Liver	ENSAMEG00000017034	PLA2G12B	phospholipase A2 group XIIB
Liver	ENSAMEG00000017044	OIT3	oncoprotein induced transcript 3
Liver	ENSAMEG00000017094	C9	complement C9
Liver	ENSAMEG00000017136	CDH16	cadherin 16
Liver	ENSAMEG00000017149	CHRDL2	chordin like 2
Liver	ENSAMEG00000017207	CES3	carboxylesterase 3
Liver	ENSAMEG00000017292	ADAMTS5	ADAM metallopeptidase with thrombospondin type 1 motif 5
Liver	ENSAMEG00000017360	NTSR1	neurotensin receptor 1
Liver	ENSAMEG00000017407	PYCR1	pyrroline-5-carboxylate reductase 1
Liver	ENSAMEG00000017498	PDZK1	PDZ domain containing 1
Liver	ENSAMEG00000017501	SLC25A18	solute carrier family 25 member 18
Liver	ENSAMEG00000017524	CFHR5	complement factor H related 5
Liver	ENSAMEG00000017529	F13B	coagulation factor XIII B chain
Liver	ENSAMEG00000017590	LCAT	lecithin-cholesterol acyltransferase
Liver	ENSAMEG00000017641	HFE2	hemochromatosis type 2 (juvenile)
Liver	ENSAMEG00000017643	GDF2	growth differentiation factor 2
Liver	ENSAMEG00000017661	HMGCS2	3-hydroxy-3-methylglutaryl-CoA synthase 2
Liver	ENSAMEG00000017673	NPC1L1	NPC1 like intracellular cholesterol transporter 1
Liver	ENSAMEG00000017684	HSD3B1	hydroxy-delta-5-steroid dehydrogenase, 3 beta- and steroid delta-isomerase 1
Liver	ENSAMEG00000017763	LEAP2	liver enriched antimicrobial peptide 2
Liver	ENSAMEG00000017827	PALD1	phosphatase domain containing, paladin 1
Liver	ENSAMEG00000017883	SLC22A12	solute carrier family 22 member 12
Liver	ENSAMEG00000017902	LECT2	leukocyte cell derived chemotaxin 2
Liver	ENSAMEG00000018006	SVEP1	sushi, von Willebrand factor type A, EGF and pentraxin domain containing 1

Liver	ENSAMEG00000018008	AKR1D1	aldo-keto reductase family 1 member D1
Liver	ENSAMEG00000018041	GSTO2	glutathione S-transferase omega 2
Liver	ENSAMEG00000018075	PBLD	phenazine biosynthesis like protein domain containing
Liver	ENSAMEG00000018103	CCDC80	coiled-coil domain containing 80
Liver	ENSAMEG00000018129	F12	coagulation factor XII
Liver	ENSAMEG00000018202	CA5A	carbonic anhydrase 5A
Liver	ENSAMEG00000018206	AMN	amnion associated transmembrane protein
Liver	ENSAMEG00000018209	PAPLN	papilin, proteoglycan like sulfated glycoprotein
Liver	ENSAMEG00000018237	TES	testin LIM domain protein
Liver	ENSAMEG00000018286	LTBP2	latent transforming growth factor beta binding protein 2
Liver	ENSAMEG00000018300	NAGS	N-acetylglutamate synthase
Liver	ENSAMEG00000018343	ERG28	ergosterol biosynthesis 28 homolog
Liver	ENSAMEG00000018429	PRSS48	serine protease 48
Liver	ENSAMEG00000018437	COLEC11	collectin subfamily member 11
Liver	ENSAMEG00000018454	SFRP2	secreted frizzled related protein 2
Liver	ENSAMEG00000018459	FGB	fibrinogen beta chain
Liver	ENSAMEG00000018462	FGA	fibrinogen alpha chain
Liver	ENSAMEG00000018463	FGG	fibrinogen gamma chain
Liver	ENSAMEG00000018551	BEX3	brain expressed X-linked 3
Liver	ENSAMEG00000018554	TCEAL9	transcription elongation factor A like 9
Liver	ENSAMEG00000018562	TRAM1L1	translocation associated membrane protein 1 like 1
Liver	ENSAMEG00000018660	ANG	angiogenin
Liver	ENSAMEG00000018665	RNASE4	ribonuclease A family member 4
Liver	ENSAMEG00000018735	CLDN14	claudin 14
Liver	ENSAMEG00000019249	FFAR3	free fatty acid receptor 3
Liver	ENSAMEG00000019276	CYP8B1	cytochrome P450 family 8 subfamily B member 1
Liver	ENSAMEG00000019355	HIST3H2A	histone cluster 3 H2A
Liver	ENSAMEG00000019531	TMEM200A	transmembrane protein 200A
Liver	ENSAMEG00000019546	GJB1	gap junction protein beta 1
Liver	ENSAMEG00000019652	--	--
Liver	ENSAMEG00000020071	LOC100474272	neuropeptide Y receptor type 4
Liver	ENSAMEG00000020133	PCDHB15	protocadherin beta-15
Liver	Novel00025	--	hypothetical protein EGM_19048, partial
Liver	Novel00227	--	--
Liver	Novel00245	--	hypothetical protein PAL_GLEAN10015091
Liver	Novel00657	--	hypothetical protein M91_03766
Liver	Novel00679	--	PREDICTED: phosphatidylinositol 4-phosphate 5-kinase type-1 beta isoform X1
Liver	Novel00753	--	PREDICTED: stimulated by retinoic acid gene 6 protein-like isoform X4
Liver	Novel00766	--	--
Liver	Novel00768	--	--
Liver	Novel00769	--	--

Liver	Novel00939	--	--
Liver	Novel00942	--	unnamed protein product
Liver	Novel00961	--	--
Liver	Novel01001	--	unknown
Liver	Novel01117	--	PREDICTED: synaptotagmin-9-like
Liver	Novel01143	--	--
Liver	Novel01307	--	PREDICTED: ankyrin repeat domain-containing protein 45
Liver	Novel01333	--	--
Liver	Novel01450	--	--
Liver	Novel01563	--	PREDICTED: CD99 antigen-like protein 2 isoform X3
Liver	Novel01568	--	--
Liver	Novel01605	--	--
Liver	Novel01798	--	PREDICTED: probable C-mannosyltransferase DPY19L2
Liver	Novel01816	--	--
Liver	Novel01887	--	--
Liver	Novel01923	--	--
Liver	Novel01981	--	--
Liver	Novel01987	--	PREDICTED: neuronal regeneration-related protein-like
Liver	Novel02034	--	--
Liver	Novel02133	--	--
Liver	Novel02138	--	--
Liver	Novel02294	--	PREDICTED: adenosine kinase-like, partial
Liver	Novel02310	--	--
Liver	Novel02311	--	PREDICTED: serpin A3-6
Liver	Novel02319	--	PREDICTED: uncharacterized protein LOC100483682
Liver	Novel02464	--	--
Liver	Novel02477	--	--
Liver	Novel02489	--	--
Liver	Novel02561	--	--
Liver	Novel02590	--	--
Liver	Novel02687	--	hypothetical protein M91_01570
Liver	Novel02759	--	--
Liver	Novel02777	--	--
Liver	Novel02826	--	hypothetical protein
Liver	Novel02865	--	--
Liver	Novel02896	--	--
Liver	Novel02972	--	--
Liver	Novel03099	--	hypothetical protein PANDA_012351
Liver	Novel03108	--	--
Liver	Novel03143	--	PREDICTED: UDP-glucuronosyltransferase 3A2 isoform X3
Liver	Novel03408	--	hypothetical protein
Liver	Novel03458	--	--
Liver	Novel03492	--	PREDICTED: olfactory receptor 6C3-like

Liver	Novel03541	--	PREDICTED: uncharacterized protein LOC105260882
Liver	Novel03547	--	hypothetical protein PANDA_014163
Liver	Novel03553	--	endonuclease/reverse transcriptase
Liver	Novel03621	--	PREDICTED: uncharacterized protein LOC105498491 isoform X2
Liver	Novel03667	--	PREDICTED: Wilms tumor protein 1-interacting protein
Liver	Novel03670	--	--
Liver	Novel03672	--	PREDICTED: CD99 antigen-like protein 2 isoform X3
Liver	Novel03726	--	--
Liver	Novel03797	--	--
Liver	Novel04234	--	--
Liver	Novel04282	--	--
Liver	Novel04298	--	PREDICTED: chymotrypsinogen B-like
Liver	Novel04335	--	PREDICTED: phospholipid-transporting ATPase IB-like
Liver	Novel04388	--	PREDICTED: LOW QUALITY PROTEIN: zinc finger protein 665-like
Liver	Novel04421	--	--
Liver	Novel04434	--	hypothetical protein M91_01570
Liver	Novel04441	--	PREDICTED: uncharacterized protein LOC102160087
Liver	Novel04509	--	--
Liver	Novel04515	--	--
Liver	Novel04616	--	--
Liver	Novel04659	--	--
Liver	Novel04705	--	unnamed protein product
Liver	Novel04706	--	PREDICTED: alpha-fetoprotein-like
Liver	Novel04720	--	--
Liver	Novel04748	--	--
Liver	Novel04903	--	--
Liver	Novel04908	--	--
Liver	Novel04923	--	--
Liver	Novel05062	--	--
Liver	Novel05211	--	--
Liver	Novel05222	--	--
Liver	Novel05274	--	hypothetical protein EGK_20962
Liver	Novel05285	--	PREDICTED: plexin-A4
Liver	Novel05311	--	PREDICTED: complement component C8 beta chain
Liver	Novel05323	--	--
Liver	Novel05345	--	PREDICTED: E3 ubiquitin-protein ligase TRIM9 isoform X4
Liver	Novel05366	--	--
Liver	Novel05391	--	--
Liver	Novel05419	--	hypothetical protein EGM_19048, partial
Liver	Novel05452	--	--
Liver	Novel05517	--	PREDICTED: UDP-glucuronosyltransferase 3A1-like, partial

Liver	Novel05518	--	--
Liver	Novel05561	--	--
Liver	Novel05606	--	PREDICTED: LOW QUALITY PROTEIN: mammaglobin-A-like
Liver	Novel05704	--	PREDICTED: cytochrome P450 4A6-like
Liver	Novel05780	--	--
Liver	Novel05953	--	--
Liver	Novel06002	--	PREDICTED: trehalase, partial
Liver	Novel06011	--	--
Spleen	ENSAMEG00000000057	CXCR5	C-X-C motif chemokine receptor 5
Spleen	ENSAMEG00000000067	--	--
Spleen	ENSAMEG00000000120	--	--
Spleen	ENSAMEG00000000135	PBK	PDZ binding kinase
Spleen	ENSAMEG00000000191	HPN	hepsin
Spleen	ENSAMEG00000000267	CDCA7	cell division cycle associated 7
Spleen	ENSAMEG00000000269	CD22	CD22 molecule
Spleen	ENSAMEG00000000271	LOC100482046	cytokine SCM-1 beta
Spleen	ENSAMEG00000000363	RORC	RAR related orphan receptor C
Spleen	ENSAMEG00000000371	CDT1	chromatin licensing and DNA replication factor 1
Spleen	ENSAMEG00000000396	MS4A1	membrane spanning 4-domains A1
Spleen	ENSAMEG00000000432	HFM1	HFM1, ATP dependent DNA helicase homolog
Spleen	ENSAMEG00000000438	MS4A4A	membrane spanning 4-domains A4A
Spleen	ENSAMEG00000000460	--	--
Spleen	ENSAMEG00000000539	CLNK	cytokine dependent hematopoietic cell linker
Spleen	ENSAMEG00000000546	TECRL	trans-2,3-enoyl-CoA reductase like
Spleen	ENSAMEG00000000558	CLPS	colipase
Spleen	ENSAMEG00000000607	SEMA3A	semaphorin 3A
Spleen	ENSAMEG00000000651	SEMA3D	semaphorin 3D
Spleen	ENSAMEG00000000693	NPHS1	NPHS1, nephrin
Spleen	ENSAMEG00000000709	STMND1	stathmin domain containing 1
Spleen	ENSAMEG00000000749	PADI6	peptidyl arginine deiminase 6
Spleen	ENSAMEG00000000751	TP53I11	tumor protein p53 inducible protein 11
Spleen	ENSAMEG00000000832	LAX1	lymphocyte transmembrane adaptor 1
Spleen	ENSAMEG00000000855	PTPRCAP	protein tyrosine phosphatase, receptor type C associated protein
Spleen	ENSAMEG00000000874	LOC100471652	T-cell surface glycoprotein CD1a
Spleen	ENSAMEG00000000898	SLC37A2	solute carrier family 37 member 2
Spleen	ENSAMEG00000000910	ETNK2	ethanolamine kinase 2
Spleen	ENSAMEG00000001125	CPNE7	copine 7
Spleen	ENSAMEG00000001169	--	--
Spleen	ENSAMEG00000001172	--	--
Spleen	ENSAMEG00000001438	--	--
Spleen	ENSAMEG00000001440	LOC100465875	zinc finger protein 671
Spleen	ENSAMEG00000001443	--	--

Spleen	ENSAMEG00000001479	CDH11	cadherin 11
Spleen	ENSAMEG00000001490	PTCHD4	patched domain containing 4
Spleen	ENSAMEG00000001508	ADAM28	ADAM metallopeptidase domain 28
Spleen	ENSAMEG00000001530	KCNQ2	potassium voltage-gated channel subfamily Q member 2
Spleen	ENSAMEG00000001613	MEP1A	meprin A subunit alpha
Spleen	ENSAMEG00000001681	ENTPD3	ectonucleoside triphosphate diphosphohydrolase 3
Spleen	ENSAMEG00000001875	SLC4A8	solute carrier family 4 member 8
Spleen	ENSAMEG00000001908	HLA-DOA	major histocompatibility complex, class II, DO alpha
Spleen	ENSAMEG00000001948	DKK2	dickkopf WNT signaling pathway inhibitor 2
Spleen	ENSAMEG00000001963	OPCML	opioid binding protein/cell adhesion molecule like
Spleen	ENSAMEG00000002090	PIK3R6	phosphoinositide-3-kinase regulatory subunit 6
Spleen	ENSAMEG00000002238	DRC1	dynein regulatory complex subunit 1
Spleen	ENSAMEG00000002259	IL17REL	interleukin 17 receptor E like
Spleen	ENSAMEG00000002276	RXFP1	relaxin/insulin like family peptide receptor 1
Spleen	ENSAMEG00000002422	LOC105234503	dynein heavy chain 5, axonemal
Spleen	ENSAMEG00000002478	SSPO	SCO-spondin
Spleen	ENSAMEG00000002513	CXCL13	C-X-C motif chemokine ligand 13
Spleen	ENSAMEG00000002553	CAPSL	calcyphosine like
Spleen	ENSAMEG00000002608	PLCH1	phospholipase C eta 1
Spleen	ENSAMEG00000002626	--	--
Spleen	ENSAMEG00000002668	MADCAM1	mucosal vascular addressin cell adhesion molecule 1
Spleen	ENSAMEG00000002723	ANKDD1A	ankyrin repeat and death domain containing 1A
Spleen	ENSAMEG00000002725	--	--
Spleen	ENSAMEG00000002754	MILR1	mast cell immunoglobulin like receptor 1
Spleen	ENSAMEG00000002762	--	--
Spleen	ENSAMEG00000002804	SDC1	syndecan 1
Spleen	ENSAMEG00000002860	ITGA8	integrin subunit alpha 8
Spleen	ENSAMEG00000002874	VSIG1	V-set and immunoglobulin domain containing 1
Spleen	ENSAMEG00000002885	CD40LG	CD40 ligand
Spleen	ENSAMEG00000002909	SCN4A	sodium voltage-gated channel alpha subunit 4
Spleen	ENSAMEG00000002977	MAPK4	mitogen-activated protein kinase 4
Spleen	ENSAMEG00000003033	CD79B	CD79b molecule
Spleen	ENSAMEG00000003038	GH1	Ailuropoda melanoleuca somatotropin (GH1), mRNA.
Spleen	ENSAMEG00000003057	--	--
Spleen	ENSAMEG00000003064	GPR39	G protein-coupled receptor 39
Spleen	ENSAMEG00000003084	PPM1K	protein phosphatase, Mg2+/Mn2+ dependent 1K
Spleen	ENSAMEG00000003144	IL12A	interleukin 12A
Spleen	ENSAMEG00000003153	STK32A	serine/threonine kinase 32A
Spleen	ENSAMEG00000003167	SCART1	scavenger receptor family member expressed on T cells 1
Spleen	ENSAMEG00000003170	TXK	TXK tyrosine kinase
Spleen	ENSAMEG00000003205	PLA2G2D	phospholipase A2 group IID
Spleen	ENSAMEG00000003290	LIMD2	LIM domain containing 2
Spleen	ENSAMEG00000003427	ERICH3	glutamate rich 3

Spleen	ENSAMEG00000003432	--	--
Spleen	ENSAMEG00000003438	CR2	complement C3d receptor 2
Spleen	ENSAMEG00000003452	DDO	D-aspartate oxidase
Spleen	ENSAMEG00000003473	TAFA3	TAFA chemokine like family member 3
Spleen	ENSAMEG00000003557	IL13RA2	interleukin 13 receptor subunit alpha 2
Spleen	ENSAMEG00000003576	SLC35F1	solute carrier family 35 member F1
Spleen	ENSAMEG00000003624	FAM184A	family with sequence similarity 184 member A
Spleen	ENSAMEG00000003676	--	--
Spleen	ENSAMEG00000003697	TNFRSF9	TNF receptor superfamily member 9
Spleen	ENSAMEG00000003730	CTNND2	catenin delta 2
Spleen	ENSAMEG00000003770	NRROS	negative regulator of reactive oxygen species
Spleen	ENSAMEG00000003831	MYBL2	MYB proto-oncogene like 2
Spleen	ENSAMEG00000003850	SBK2	SH3 domain binding kinase family member 2
Spleen	ENSAMEG00000003951	TLX3	T cell leukemia homeobox 3
Spleen	ENSAMEG00000004167	C11orf52	chromosome 11 open reading frame 52
Spleen	ENSAMEG00000004195	ARL5C	ADP ribosylation factor like GTPase 5C
Spleen	ENSAMEG00000004338	POU2AF1	POU class 2 associating factor 1
Spleen	ENSAMEG00000004450	--	--
Spleen	ENSAMEG00000004457	CD5L	CD5 molecule like
Spleen	ENSAMEG00000004479	CD79A	CD79a molecule
Spleen	ENSAMEG00000004534	TMEM255B	transmembrane protein 255B
Spleen	ENSAMEG00000004633	DCLK1	doublecortin like kinase 1
Spleen	ENSAMEG00000004634	KLRD1	killer cell lectin like receptor D1
Spleen	ENSAMEG00000004661	TBX21	T-box 21
Spleen	ENSAMEG00000004693	CLDN11	claudin 11
Spleen	ENSAMEG00000004721	EDIL3	EGF like repeats and discoidin domains 3
Spleen	ENSAMEG00000004752	GRTP1	growth hormone regulated TBC protein 1
Spleen	ENSAMEG00000004848	--	--
Spleen	ENSAMEG00000004883	IGHM	immunoglobulin heavy constant mu
Spleen	ENSAMEG00000004905	--	--
Spleen	ENSAMEG00000005020	PLAC8	placenta specific 8
Spleen	ENSAMEG00000005154	NOS2	nitric oxide synthase 2
Spleen	ENSAMEG00000005181	ZAP70	zeta chain of T cell receptor associated protein kinase 70
Spleen	ENSAMEG00000005207	NOX4	NADPH oxidase 4
Spleen	ENSAMEG00000005233	PDLIM3	PDZ and LIM domain 3
Spleen	ENSAMEG00000005259	FOLH1	folate hydrolase 1
Spleen	ENSAMEG00000005310	ARHGEF37	Rho guanine nucleotide exchange factor 37
Spleen	ENSAMEG00000005530	BICDL2	BICD family like cargo adaptor 2
Spleen	ENSAMEG00000005829	--	--
Spleen	ENSAMEG00000005851	ROS1	ROS proto-oncogene 1, receptor tyrosine kinase
Spleen	ENSAMEG00000005863	JCHAIN	joining chain of multimeric IgA and IgM
Spleen	ENSAMEG00000005942	MSC	musculin
Spleen	ENSAMEG00000005998	ITK	IL2 inducible T cell kinase

Spleen	ENSAMEG00000006136	BLK	BLK proto-oncogene, Src family tyrosine kinase
Spleen	ENSAMEG00000006223	EPCAM	epithelial cell adhesion molecule
Spleen	ENSAMEG00000006268	NIPAL4	NIPA like domain containing 4
Spleen	ENSAMEG00000006286	EPHA5	EPH receptor A5
Spleen	ENSAMEG00000006292	--	--
Spleen	ENSAMEG00000006302	--	--
Spleen	ENSAMEG00000006311	TRBV16	T cell receptor beta variable 16 (gene/pseudogene)
Spleen	ENSAMEG00000006312	TRBV19	T cell receptor beta variable 19
Spleen	ENSAMEG00000006313	--	--
Spleen	ENSAMEG00000006400	SDK1	sidekick cell adhesion molecule 1
Spleen	ENSAMEG00000006445	KCNIP4	potassium voltage-gated channel interacting protein 4
Spleen	ENSAMEG00000006673	NAV3	neuron navigator 3
Spleen	ENSAMEG00000006749	GABRA1	gamma-aminobutyric acid type A receptor alpha1 subunit
Spleen	ENSAMEG00000006799	NYX	nyctalopin
Spleen	ENSAMEG00000006830	LOC105234899	anosmin-1
Spleen	ENSAMEG00000006927	CPLX2	complexin 2
Spleen	ENSAMEG00000006972	WWC1	WW and C2 domain containing 1
Spleen	ENSAMEG00000006985	PCED1B	PC-esterase domain containing 1B
Spleen	ENSAMEG00000007018	ABCC2	ATP binding cassette subfamily C member 2
Spleen	ENSAMEG00000007039	--	--
Spleen	ENSAMEG00000007082	AICDA	activation induced cytidine deaminase
Spleen	ENSAMEG00000007133	TRDC	T cell receptor delta constant
Spleen	ENSAMEG00000007135	--	--
Spleen	ENSAMEG00000007231	AK5	adenylate kinase 5
Spleen	ENSAMEG00000007274	BRINP1	BMP/retinoic acid inducible neural specific 1
Spleen	ENSAMEG00000007277	NKX2-3	NK2 homeobox 3
Spleen	ENSAMEG00000007409	GLB1L2	galactosidase beta 1 like 2
Spleen	ENSAMEG00000007493	CD6	CD6 molecule
Spleen	ENSAMEG00000007547	CD5	CD5 molecule
Spleen	ENSAMEG00000007637	CACNA1E	calcium voltage-gated channel subunit alpha1 E
Spleen	ENSAMEG00000007639	PLD4	phospholipase D family member 4
Spleen	ENSAMEG00000007672	LCK	LCK proto-oncogene, Src family tyrosine kinase
Spleen	ENSAMEG00000007735	IGHV6-1	immunoglobulin heavy variable 6-1
Spleen	ENSAMEG00000007862	COBL	cordon-bleu WH2 repeat protein
Spleen	ENSAMEG00000007865	SEPTIN1	septin 1
Spleen	ENSAMEG00000007896	KCNQ5	potassium voltage-gated channel subfamily Q member 5
Spleen	ENSAMEG00000007935	DSC2	desmocollin 2
Spleen	ENSAMEG00000008065	SCML4	Scm polycomb group protein like 4
Spleen	ENSAMEG00000008079	--	--
Spleen	ENSAMEG00000008093	SLC36A2	solute carrier family 36 member 2
Spleen	ENSAMEG00000008164	DSG2	desmoglein 2
Spleen	ENSAMEG00000008188	--	--
Spleen	ENSAMEG00000008189	--	--

Spleen	ENSAMEG00000008190	--	--
Spleen	ENSAMEG00000008217	--	--
Spleen	ENSAMEG00000008247	--	--
Spleen	ENSAMEG00000008265	LOC105238776	uncharacterized LOC105238776
Spleen	ENSAMEG00000008395	CRTAM	cytotoxic and regulatory T cell molecule
Spleen	ENSAMEG00000008423	--	--
Spleen	ENSAMEG00000008427	--	--
Spleen	ENSAMEG00000008447	CLMP	CXADR like membrane protein
Spleen	ENSAMEG00000008550	--	--
Spleen	ENSAMEG00000008683	--	--
Spleen	ENSAMEG00000008750	--	--
Spleen	ENSAMEG00000008793	CPLX3	complexin 3
Spleen	ENSAMEG00000008796	LMAN1L	lectin, mannose binding 1 like
Spleen	ENSAMEG00000008811	ISM1	isthmin 1
Spleen	ENSAMEG00000008823	CNTNAP5	contactin associated protein like 5
Spleen	ENSAMEG00000008842	CYP1A2	cytochrome P450 family 1 subfamily A member 2
Spleen	ENSAMEG00000008865	TNFSF11	TNF superfamily member 11
Spleen	ENSAMEG00000008876	BAIAP2L1	BAI1 associated protein 2 like 1
Spleen	ENSAMEG00000008898	CYP1A1	cytochrome P450 family 1 subfamily A member 1
Spleen	ENSAMEG00000008911	--	--
Spleen	ENSAMEG00000008913	--	--
Spleen	ENSAMEG00000009037	NOX1	NADPH oxidase 1
Spleen	ENSAMEG00000009069	CYP11A1	cytochrome P450 family 11 subfamily A member 1
Spleen	ENSAMEG00000009121	XKRX	XK related, X-linked
Spleen	ENSAMEG00000009237	TP73	tumor protein p73
Spleen	ENSAMEG00000009264	C15orf59	chromosome 15 open reading frame 59
Spleen	ENSAMEG00000009342	NKX3-2	NK3 homeobox 2
Spleen	ENSAMEG00000009362	TNFRSF13C	TNF receptor superfamily member 13C
Spleen	ENSAMEG00000009399	P2RX1	purinergic receptor P2X 1
Spleen	ENSAMEG00000009412	ATP2A3	ATPase sarcoplasmic/endoplasmic reticulum Ca2+ transporting 3
Spleen	ENSAMEG00000009419	ECHDC2	enoyl-CoA hydratase domain containing 2
Spleen	ENSAMEG00000009437	STYK1	serine/threonine/tyrosine kinase 1
Spleen	ENSAMEG00000009440	LOC100472452	cytochrome P450 2D15
Spleen	ENSAMEG00000009473	CD72	CD72 molecule
Spleen	ENSAMEG00000009479	--	--
Spleen	ENSAMEG00000009488	LTA	lymphotoxin alpha
Spleen	ENSAMEG00000009525	--	--
Spleen	ENSAMEG00000009547	POLE2	DNA polymerase epsilon 2, accessory subunit
Spleen	ENSAMEG00000009613	TNFRSF17	TNF receptor superfamily member 17
Spleen	ENSAMEG00000009652	PGBD5	piggyBac transposable element derived 5
Spleen	ENSAMEG00000009730	ALDH8A1	aldehyde dehydrogenase 8 family member A1
Spleen	ENSAMEG00000009859	LGALS4	galectin 4

Spleen	ENSAMEG00000009888	IKZF3	IKAROS family zinc finger 3
Spleen	ENSAMEG00000010008	CD8A	CD8a molecule
Spleen	ENSAMEG00000010032	TMEM156	transmembrane protein 156
Spleen	ENSAMEG00000010071	NCMAP	non-compact myelin associated protein
Spleen	ENSAMEG00000010102	RUNX3	runt related transcription factor 3
Spleen	ENSAMEG00000010113	SLA2	Src like adaptor 2
Spleen	ENSAMEG00000010127	PAX5	paired box 5
Spleen	ENSAMEG00000010156	PERP	PERP, TP53 apoptosis effector
Spleen	ENSAMEG00000010166	--	--
Spleen	ENSAMEG00000010173	--	--
Spleen	ENSAMEG00000010182	MAP4K1	mitogen-activated protein kinase kinase kinase kinase 1
Spleen	ENSAMEG00000010185	--	--
Spleen	ENSAMEG00000010189	--	--
Spleen	ENSAMEG00000010191	TRAT1	T cell receptor associated transmembrane adaptor 1
Spleen	ENSAMEG00000010196	F2RL3	F2R like thrombin or trypsin receptor 3
Spleen	ENSAMEG00000010234	SOX21	SRY-box 21
Spleen	ENSAMEG00000010299	PLPP4	phospholipid phosphatase 4
Spleen	ENSAMEG00000010347	CNTN1	contactin 1
Spleen	ENSAMEG00000010373	PKP3	plakophilin 3
Spleen	ENSAMEG00000010399	GRPR	gastrin releasing peptide receptor
Spleen	ENSAMEG00000010411	BAIAP2L2	BAI1 associated protein 2 like 2
Spleen	ENSAMEG00000010441	--	--
Spleen	ENSAMEG00000010598	TSPEAR	thrombospondin type laminin G domain and EAR repeats
Spleen	ENSAMEG00000010631	FAM129C	family with sequence similarity 129 member C
Spleen	ENSAMEG00000010650	--	--
Spleen	ENSAMEG00000010658	FAM20A	FAM20A, golgi associated secretory pathway pseudokinase
Spleen	ENSAMEG00000010676	NLRC3	NLR family CARD domain containing 3
Spleen	ENSAMEG00000010699	EPS8L2	EPS8 like 2
Spleen	ENSAMEG00000010758	FCHO1	FCH domain only 1
Spleen	ENSAMEG00000010800	--	--
Spleen	ENSAMEG00000010836	SIGLEC1	sialic acid binding Ig like lectin 1
Spleen	ENSAMEG00000011003	GRAP2	GRB2-related adaptor protein 2
Spleen	ENSAMEG00000011008	IL12RB1	interleukin 12 receptor subunit beta 1
Spleen	ENSAMEG00000011163	IL21R	interleukin 21 receptor
Spleen	ENSAMEG00000011166	NR5A1	nuclear receptor subfamily 5 group A member 1
Spleen	ENSAMEG00000011277	CUNH4orf19	chromosome unknown C4orf19 homolog
Spleen	ENSAMEG00000011326	STC2	stanniocalcin 2
Spleen	ENSAMEG00000011394	--	--
Spleen	ENSAMEG00000011397	--	--
Spleen	ENSAMEG00000011400	--	--
Spleen	ENSAMEG00000011427	LMO7	LIM domain 7
Spleen	ENSAMEG00000011477	--	--
Spleen	ENSAMEG00000011507	--	--

Spleen	ENSAMEG00000011508	CCK	cholecystokinin
Spleen	ENSAMEG00000011515	DOCK10	dedicator of cytokinesis 10
Spleen	ENSAMEG00000011517	CERS1	ceramide synthase 1
Spleen	ENSAMEG00000011584	WNT5A	Wnt family member 5A
Spleen	ENSAMEG00000011595	HOMER3	homer scaffolding protein 3
Spleen	ENSAMEG00000011640	TMEM91	transmembrane protein 91
Spleen	ENSAMEG00000011678	RNF43	ring finger protein 43
Spleen	ENSAMEG00000011779	HSPB7	heat shock protein family B (small) member 7
Spleen	ENSAMEG00000011835	LOC100470434	proline dehydrogenase 1, mitochondrial
Spleen	ENSAMEG00000011844	SLC25A34	solute carrier family 25 member 34
Spleen	ENSAMEG00000011859	ARFGEF3	ARFGEF family member 3
Spleen	ENSAMEG00000011870	SLC38A3	solute carrier family 38 member 3
Spleen	ENSAMEG00000011916	CDH2	cadherin 2
Spleen	ENSAMEG00000011924	LOC100470574	receptor-type tyrosine-protein phosphatase V-like
Spleen	ENSAMEG00000011943	KRT8	keratin 8
Spleen	ENSAMEG00000011948	LOC105241797	killer cell lectin-like receptor 5
Spleen	ENSAMEG00000011977	FCRL5	Fc receptor like 5
Spleen	ENSAMEG00000011981	KRT18	keratin 18
Spleen	ENSAMEG00000011985	--	--
Spleen	ENSAMEG00000011988	KLRK1	killer cell lectin like receptor K1
Spleen	ENSAMEG00000012007	--	--
Spleen	ENSAMEG00000012009	FCRL3	Fc receptor like 3
Spleen	ENSAMEG00000012066	ACOX2	acyl-CoA oxidase 2
Spleen	ENSAMEG00000012147	LAD1	ladinin 1
Spleen	ENSAMEG00000012265	TNFRSF13B	TNF receptor superfamily member 13B
Spleen	ENSAMEG00000012342	SSUH2	ssu-2 homolog (C. elegans)
Spleen	ENSAMEG00000012376	CMTM8	CKLF like MARVEL transmembrane domain containing 8
Spleen	ENSAMEG00000012439	FMOD	fibromodulin
Spleen	ENSAMEG00000012458	EDN3	endothelin 3
Spleen	ENSAMEG00000012579	PSTPIP2	proline-serine-threonine phosphatase interacting protein 2
Spleen	ENSAMEG00000012617	--	--
Spleen	ENSAMEG00000012699	AVPR1B	arginine vasopressin receptor 1B
Spleen	ENSAMEG00000012729	--	--
Spleen	ENSAMEG00000012731	EOMES	eomesodermin
Spleen	ENSAMEG00000012746	CAMK4	calcium/calmodulin dependent protein kinase IV
Spleen	ENSAMEG00000012764	NEGR1	neuronal growth regulator 1
Spleen	ENSAMEG00000012786	PTCRA	pre T cell antigen receptor alpha
Spleen	ENSAMEG00000012856	SLA	Src like adaptor
Spleen	ENSAMEG00000012919	KIF21A	kinesin family member 21A
Spleen	ENSAMEG00000013028	RGN	regucalcin
Spleen	ENSAMEG00000013107	SPIB	Spi-B transcription factor
Spleen	ENSAMEG00000013141	CD3G	CD3g molecule
Spleen	ENSAMEG00000013155	CD3D	CD3d molecule

Spleen	ENSAMEG00000013166	CD3E	CD3e molecule
Spleen	ENSAMEG00000013306	MYH14	myosin heavy chain 14
Spleen	ENSAMEG00000013475	CCDC3	coiled-coil domain containing 3
Spleen	ENSAMEG00000013498	CLSTN3	calsyntenin 3
Spleen	ENSAMEG00000013523	GABRD	gamma-aminobutyric acid type A receptor delta subunit
Spleen	ENSAMEG00000013547	--	--
Spleen	ENSAMEG00000013613	TNFRSF4	TNF receptor superfamily member 4
Spleen	ENSAMEG00000013628	--	--
Spleen	ENSAMEG00000013800	TMC8	transmembrane channel like 8
Spleen	ENSAMEG00000013858	--	--
Spleen	ENSAMEG00000013909	LOC105241990	contactin-associated protein-like 5
Spleen	ENSAMEG00000014075	CYP4B1	cytochrome P450 family 4 subfamily B member 1
Spleen	ENSAMEG00000014082	LAMB4	laminin subunit beta 4
Spleen	ENSAMEG00000014087	--	--
Spleen	ENSAMEG00000014088	SLAMF7	SLAM family member 7
Spleen	ENSAMEG00000014107	GKN2	gastrokine 2
Spleen	ENSAMEG00000014126	RSPO4	R-spondin 4
Spleen	ENSAMEG00000014283	--	--
Spleen	ENSAMEG00000014285	--	--
Spleen	ENSAMEG00000014350	AREG	amphiregulin
Spleen	ENSAMEG00000014444	PRG2	proteoglycan 2, pro eosinophil major basic protein
Spleen	ENSAMEG00000014466	CD27	CD27 molecule
Spleen	ENSAMEG00000014507	SH3GL3	SH3 domain containing GRB2 like 3, endophilin A3
Spleen	ENSAMEG00000014522	GUCY1A2	guanylate cyclase 1 soluble subunit alpha 2
Spleen	ENSAMEG00000014612	MAPT	microtubule associated protein tau
Spleen	ENSAMEG00000014643	PI15	peptidase inhibitor 15
Spleen	ENSAMEG00000014693	NPY1R	neuropeptide Y receptor Y1
Spleen	ENSAMEG00000014699	NPY5R	neuropeptide Y receptor Y5
Spleen	ENSAMEG00000014704	ITGB3	integrin subunit beta 3
Spleen	ENSAMEG00000014728	MMD	monocyte to macrophage differentiation associated
Spleen	ENSAMEG00000014806	ADCY1	adenylate cyclase 1
Spleen	ENSAMEG00000014889	ATF7IP2	activating transcription factor 7 interacting protein 2
Spleen	ENSAMEG00000015007	SH2D2A	SH2 domain containing 2A
Spleen	ENSAMEG00000015066	GDNF	glial cell derived neurotrophic factor
Spleen	ENSAMEG00000015075	--	--
Spleen	ENSAMEG00000015225	ELL3	elongation factor for RNA polymerase II 3
Spleen	ENSAMEG00000015232	NR1H4	nuclear receptor subfamily 1 group H member 4
Spleen	ENSAMEG00000015552	ZP3	zona pellucida glycoprotein 3
Spleen	ENSAMEG00000015558	SCN1A	sodium voltage-gated channel alpha subunit 1
Spleen	ENSAMEG00000015664	RORB	RAR related orphan receptor B
Spleen	ENSAMEG00000015679	CD19	CD19 molecule
Spleen	ENSAMEG00000015681	LY9	lymphocyte antigen 9
Spleen	ENSAMEG00000015682	--	--

Spleen	ENSAMEG00000015726	SH2D1A	SH2 domain containing 1A
Spleen	ENSAMEG00000015734	RYR3	ryanodine receptor 3
Spleen	ENSAMEG00000015813	BCL11A	B cell CLL/lymphoma 11A
Spleen	ENSAMEG00000015837	STXBP6	syntaxin binding protein 6
Spleen	ENSAMEG00000015997	LAMP3	lysosomal associated membrane protein 3
Spleen	ENSAMEG00000016011	PRRX2	paired related homeobox 2
Spleen	ENSAMEG00000016044	LOC100468389	immunoglobulin omega chain
Spleen	ENSAMEG00000016128	WSCD2	WSC domain containing 2
Spleen	ENSAMEG00000016211	CNTNAP4	contactin associated protein like 4
Spleen	ENSAMEG00000016331	MAOB	monoamine oxidase B
Spleen	ENSAMEG00000016382	ASGR1	asialoglycoprotein receptor 1
Spleen	ENSAMEG00000016415	IRX6	iroquois homeobox 6
Spleen	ENSAMEG00000016420	SPTBN5	spectrin beta, non-erythrocytic 5
Spleen	ENSAMEG00000016464	SPIC	Spi-C transcription factor
Spleen	ENSAMEG00000016521	OLFML2A	olfactomedin like 2A
Spleen	ENSAMEG00000016556	ITGBL1	integrin subunit beta like 1
Spleen	ENSAMEG00000017019	DLL1	delta like canonical Notch ligand 1
Spleen	ENSAMEG00000017060	ADGRG5	adhesion G protein-coupled receptor G5
Spleen	ENSAMEG00000017094	C9	complement C9
Spleen	ENSAMEG00000017114	TMEM151A	transmembrane protein 151A
Spleen	ENSAMEG00000017246	DDAH1	dimethylarginine dimethylaminohydrolase 1
Spleen	ENSAMEG00000017354	DSP	desmoplakin
Spleen	ENSAMEG00000017416	LY86	lymphocyte antigen 86
Spleen	ENSAMEG00000017460	CARMIL2	capping protein regulator and myosin 1 linker 2
Spleen	ENSAMEG00000017469	FCMR	Fc fragment of IgM receptor
Spleen	ENSAMEG00000017474	IL24	interleukin 24
Spleen	ENSAMEG00000017521	CD160	CD160 molecule
Spleen	ENSAMEG00000017527	STMN2	stathmin 2
Spleen	ENSAMEG00000017567	SH3RF2	SH3 domain containing ring finger 2
Spleen	ENSAMEG00000017604	TLX1	T cell leukemia homeobox 1
Spleen	ENSAMEG00000017709	--	--
Spleen	ENSAMEG00000018010	CAMK1G	calcium/calmodulin dependent protein kinase IG
Spleen	ENSAMEG00000018026	PTPN3	protein tyrosine phosphatase, non-receptor type 3
Spleen	ENSAMEG00000018031	LEPR	leptin receptor
Spleen	ENSAMEG00000018076	PTN	pleiotrophin
Spleen	ENSAMEG00000018086	ASPG	asparaginase
Spleen	ENSAMEG00000018123	BTLA	B and T lymphocyte associated
Spleen	ENSAMEG00000018144	GCSAM	germinal center associated signaling and motility
Spleen	ENSAMEG00000018148	TAGLN3	transgelin 3
Spleen	ENSAMEG00000018170	CD96	CD96 molecule
Spleen	ENSAMEG00000018202	CA5A	carbonic anhydrase 5A
Spleen	ENSAMEG00000018311	RPS6KL1	ribosomal protein S6 kinase like 1
Spleen	ENSAMEG00000018338	BATF	basic leucine zipper ATF-like transcription factor

Spleen	ENSAMEG00000018458	ARHGAP4	Rho GTPase activating protein 4
Spleen	ENSAMEG00000018488	TLR10	toll like receptor 10
Spleen	ENSAMEG00000018603	KCNE4	potassium voltage-gated channel subfamily E regulatory subunit 4
Spleen	ENSAMEG00000018668	--	--
Spleen	ENSAMEG00000018677	LOC100482217	profilin-1
Spleen	ENSAMEG00000018739	--	--
Spleen	ENSAMEG00000018766	--	--
Spleen	ENSAMEG00000018799	--	--
Spleen	ENSAMEG00000018807	CHST1	carbohydrate sulfotransferase 1
Spleen	ENSAMEG00000018810	GPR55	G protein-coupled receptor 55
Spleen	ENSAMEG00000018822	TMEM125	transmembrane protein 125
Spleen	ENSAMEG00000018850	PLN	phospholamban
Spleen	ENSAMEG00000018888	--	--
Spleen	ENSAMEG00000018906	LOC100474158	olfactory receptor 10G9
Spleen	ENSAMEG00000019079	P2RY13	purinergic receptor P2Y13
Spleen	ENSAMEG00000019089	DIRAS2	DIRAS family GTPase 2
Spleen	ENSAMEG00000019097	LINGO4	leucine rich repeat and Ig domain containing 4
Spleen	ENSAMEG00000019299	--	--
Spleen	ENSAMEG00000019310	--	--
Spleen	ENSAMEG00000019396	XCR1	X-C motif chemokine receptor 1
Spleen	ENSAMEG00000019512	--	--
Spleen	ENSAMEG00000019552	--	--
Spleen	ENSAMEG00000019556	--	--
Spleen	ENSAMEG00000019561	--	--
Spleen	ENSAMEG00000019586	AKAP5	A-kinase anchoring protein 5
Spleen	ENSAMEG00000019595	CCR6	C-C motif chemokine receptor 6
Spleen	ENSAMEG00000019672	--	--
Spleen	ENSAMEG00000019767	CXCR3	C-X-C motif chemokine receptor 3
Spleen	ENSAMEG00000019777	TLR7	toll like receptor 7
Spleen	ENSAMEG00000019880	LRRN2	leucine rich repeat neuronal 2
Spleen	ENSAMEG00000019904	GPR65	G protein-coupled receptor 65
Spleen	ENSAMEG00000019956	--	--
Spleen	ENSAMEG00000020056	RNASE13	ribonuclease A family member 13 (inactive)
Spleen	ENSAMEG00000020100	CH25H	cholesterol 25-hydroxylase
Spleen	ENSAMEG00000020118	GPR18	G protein-coupled receptor 18
Spleen	ENSAMEG00000020141	SMIM33	small integral membrane protein 33
Spleen	ENSAMEG00000020150	--	--
Spleen	ENSAMEG00000020169	P2RY10	P2Y receptor family member 10
Spleen	ENSAMEG00000020171	GPR174	G protein-coupled receptor 174
Spleen	ENSAMEG00000020236	--	--
Spleen	Novel00102	--	neurotrimin, isoform CRA_b
Spleen	Novel00110	--	hypothetical protein

Spleen	Novel00149	--	--
Spleen	Novel00192	--	PREDICTED: collagen alpha-1(III) chain-like
Spleen	Novel00204	--	--
Spleen	Novel00228	--	PREDICTED: fibrous sheath-interacting protein 1 isoform X2
Spleen	Novel00253	--	hypothetical protein EGM_18659, partial
Spleen	Novel00314	--	--
Spleen	Novel00336	--	--
Spleen	Novel00372	--	--
Spleen	Novel00416	--	hypothetical protein, conserved
Spleen	Novel00421	--	--
Spleen	Novel00422	--	--
Spleen	Novel00494	--	--
Spleen	Novel00495	--	PREDICTED: uncharacterized protein LOC100464472, partial
Spleen	Novel00505	--	unnamed protein product
Spleen	Novel00507	--	PREDICTED: alpha-(1,6)-fucosyltransferase isoform X3
Spleen	Novel00589	--	--
Spleen	Novel00654	--	--
Spleen	Novel00706	--	--
Spleen	Novel00889	--	--
Spleen	Novel00969	--	--
Spleen	Novel00996	--	--
Spleen	Novel01035	--	PREDICTED: N-acetyllactosaminide beta-1,6-N-acetylglucosaminyl-transferase, isoform A-like
Spleen	Novel01061	--	--
Spleen	Novel01062	--	PREDICTED: uncharacterized protein LOC100737626
Spleen	Novel01068	--	--
Spleen	Novel01085	--	--
Spleen	Novel01169	--	--
Spleen	Novel01410	--	--
Spleen	Novel01424	--	--
Spleen	Novel01454	--	--
Spleen	Novel01457	--	PREDICTED: uncharacterized protein LOC609006
Spleen	Novel01464	--	--
Spleen	Novel01475	--	--
Spleen	Novel01503	--	--
Spleen	Novel01507	--	--
Spleen	Novel01591	--	--
Spleen	Novel01600	--	--
Spleen	Novel01602	--	--
Spleen	Novel01636	--	PREDICTED: homeodomain-only protein isoform X1
Spleen	Novel01645	--	--
Spleen	Novel01659	--	--

Spleen	Novel01676	--	--
Spleen	Novel01687	--	--
Spleen	Novel01710	--	hypothetical protein M91_18372, partial
Spleen	Novel01738	--	--
Spleen	Novel01758	--	PREDICTED: uncharacterized protein LOC105582071
Spleen	Novel01759	--	--
Spleen	Novel01840	--	--
Spleen	Novel01866	--	PREDICTED: proline-rich protein 2-like
Spleen	Novel01924	--	LRRGT00194
Spleen	Novel02011	--	--
Spleen	Novel02030	--	--
Spleen	Novel02036	--	--
Spleen	Novel02053	--	--
Spleen	Novel02089	--	--
Spleen	Novel02091	--	LRRG00130
Spleen	Novel02106	--	--
Spleen	Novel02146	--	hypothetical protein M91_20222, partial
Spleen	Novel02157	--	PREDICTED: unconventional myosin-XVB-like
Spleen	Novel02195	--	PREDICTED: LOW QUALITY PROTEIN: liprin-alpha-2
Spleen	Novel02220	--	--
Spleen	Novel02221	--	--
Spleen	Novel02237	--	PREDICTED: Fc receptor-like A isoform X1
Spleen	Novel02253	--	--
Spleen	Novel02301	--	--
Spleen	Novel02535	--	PREDICTED: LOW QUALITY PROTEIN: uncharacterized protein LOC105944309
Spleen	Novel02538	--	--
Spleen	Novel02558	--	--
Spleen	Novel02560	--	PREDICTED: solute carrier family 40 member 1-like
Spleen	Novel02588	--	--
Spleen	Novel02653	--	T cell receptor beta chain, partial
Spleen	Novel02686	--	--
Spleen	Novel02794	--	--
Spleen	Novel02809	--	--
Spleen	Novel02810	--	--
Spleen	Novel02813	--	--
Spleen	Novel02814	--	--
Spleen	Novel02827	--	LRRGT00191
Spleen	Novel02845	--	--
Spleen	Novel02947	--	hCG1820541, partial
Spleen	Novel03051	--	--
Spleen	Novel03124	--	PREDICTED: catenin delta-2-like
Spleen	Novel03158	--	--

Spleen	Novel03163	--	unnamed protein product
Spleen	Novel03239	--	--
Spleen	Novel03265	--	--
Spleen	Novel03268	--	--
Spleen	Novel03320	--	unnamed protein product
Spleen	Novel03410	--	--
Spleen	Novel03428	--	hypothetical protein EGM_19048, partial
Spleen	Novel03429	--	PREDICTED: uncharacterized protein LOC101134168
Spleen	Novel03492	--	PREDICTED: olfactory receptor 6C3-like
Spleen	Novel03495	--	--
Spleen	Novel03508	--	--
Spleen	Novel03532	--	--
Spleen	Novel03543	--	--
Spleen	Novel03551	--	protein Shroom4-like protein
Spleen	Novel03601	--	--
Spleen	Novel03646	--	--
Spleen	Novel03675	--	--
Spleen	Novel03806	--	--
Spleen	Novel03845	--	hCG1759600
Spleen	Novel03909	--	hypothetical protein M91_11495, partial
Spleen	Novel03973	--	endonuclease/reverse transcriptase
Spleen	Novel03975	--	--
Spleen	Novel03976	--	--
Spleen	Novel03977	--	hypothetical protein PANDA_015862
Spleen	Novel03988	--	unnamed protein product
Spleen	Novel03989	--	PREDICTED: tigger transposable element-derived protein 1-like
Spleen	Novel04004	--	hypothetical protein EGM_06233, partial
Spleen	Novel04047	--	--
Spleen	Novel04067	--	PREDICTED: tripartite motif-containing protein 34-like isoform X3
Spleen	Novel04068	--	--
Spleen	Novel04101	--	--
Spleen	Novel04235	--	PREDICTED: stathmin domain-containing protein 1
Spleen	Novel04286	--	--
Spleen	Novel04346	--	--
Spleen	Novel04355	--	--
Spleen	Novel04371	--	--
Spleen	Novel04445	--	PREDICTED: LOW QUALITY PROTEIN: collagen alpha-4(VI) chain-like
Spleen	Novel04465	--	--
Spleen	Novel04479	--	hypothetical protein M91_12176, partial
Spleen	Novel04539	--	hypothetical protein M91_00785, partial

Spleen	Novel04574	--	--
Spleen	Novel04611	--	--
Spleen	Novel04690	--	--
Spleen	Novel04692	--	similar to ribosomal protein L17
Spleen	Novel04723	--	endonuclease/reverse transcriptase
Spleen	Novel04760	--	PREDICTED: T-lymphocyte surface antigen Ly-9-like, partial
Spleen	Novel04792	--	--
Spleen	Novel04888	--	--
Spleen	Novel04891	--	--
Spleen	Novel04892	--	PREDICTED: calmodulin
Spleen	Novel04915	--	--
Spleen	Novel04961	--	--
Spleen	Novel05049	--	PREDICTED: receptor-type tyrosine-protein phosphatase U-like
Spleen	Novel05192	--	PREDICTED: uncharacterized protein LOC105234449
Spleen	Novel05267	--	PREDICTED: T-cell receptor gamma chain C region C10.5
Spleen	Novel05268	--	hypothetical protein PANDA_020698, partial
Spleen	Novel05320	--	--
Spleen	Novel05363	--	PREDICTED: guanylate cyclase soluble subunit alpha-2-like
Spleen	Novel05368	--	hypothetical protein PANDA_020949, partial
Spleen	Novel05482	--	--
Spleen	Novel05519	--	--
Spleen	Novel05586	--	PREDICTED: CMRF35-like molecule 7
Spleen	Novel05601	--	--
Spleen	Novel05602	--	PREDICTED: Ig mu chain C region membrane-bound form isoform X5
Spleen	Novel05630	--	--
Spleen	Novel05699	--	hypothetical protein PANDA_022568, partial
Spleen	Novel05700	--	hypothetical protein M91_07955
Spleen	Novel05756	--	hypothetical protein PANDA_022245, partial
Spleen	Novel05789	--	hypothetical protein PANDA_022245, partial
Spleen	Novel05913	--	hypothetical protein PANDA_022433, partial
Spleen	Novel05962	--	--
Spleen	Novel06007	--	Ig lambda chain V-I region BL2
Lung	ENSAMEG00000000066	ACP5	acid phosphatase 5, tartrate resistant
Lung	ENSAMEG00000000092	--	--
Lung	ENSAMEG00000000119	--	--
Lung	ENSAMEG00000000120	--	--
Lung	ENSAMEG00000000260	TACSTD2	tumor associated calcium signal transducer 2
Lung	ENSAMEG00000000330	ZNF385B	zinc finger protein 385B
Lung	ENSAMEG00000000473	TEKT2	tektin 2
Lung	ENSAMEG00000000567	UPK1A	uroplakin 1A
Lung	ENSAMEG00000000586	FGF10	fibroblast growth factor 10

Lung	ENSAMEG00000000761	VSIG2	V-set and immunoglobulin domain containing 2
Lung	ENSAMEG00000000776	KIAA1324L	KIAA1324 like
Lung	ENSAMEG00000000840	LOC100470906	T-cell surface glycoprotein CD1a
Lung	ENSAMEG00000000847	LOC100471155	T-cell surface glycoprotein CD1a
Lung	ENSAMEG00000000874	LOC100471652	T-cell surface glycoprotein CD1a
Lung	ENSAMEG00000000931	FHOD3	formin homology 2 domain containing 3
Lung	ENSAMEG00000000944	TMEM212	transmembrane protein 212
Lung	ENSAMEG00000000972	SCGB3A1	secretoglobin family 3A member 1
Lung	ENSAMEG00000000994	KNG1	kininogen 1
Lung	ENSAMEG00000001045	C5	complement C5
Lung	ENSAMEG00000001068	RSPO1	R-spondin 1
Lung	ENSAMEG00000001184	CFTR	cystic fibrosis transmembrane conductance regulator
Lung	ENSAMEG00000001313	--	--
Lung	ENSAMEG00000001329	KIF19	kinesin family member 19
Lung	ENSAMEG00000001508	ADAM28	ADAM metallopeptidase domain 28
Lung	ENSAMEG00000001535	SHC4	SHC adaptor protein 4
Lung	ENSAMEG00000001573	LOC100481102	keratin, type II cuticular Hb1
Lung	ENSAMEG00000001642	EFCAB1	EF-hand calcium binding domain 1
Lung	ENSAMEG00000001650	ANKRD66	ankyrin repeat domain 66
Lung	ENSAMEG00000001677	FCRLB	Fc receptor like B
Lung	ENSAMEG00000001683	KRT7	keratin 7
Lung	ENSAMEG00000001688	GPR68	G protein-coupled receptor 68
Lung	ENSAMEG00000001725	C1orf54	chromosome 1 open reading frame 54
Lung	ENSAMEG00000001738	IQCA1	IQ motif containing with AAA domain 1
Lung	ENSAMEG00000001749	--	--
Lung	ENSAMEG00000001762	SMIM41	small integral membrane protein 41
Lung	ENSAMEG00000001798	FAM183A	family with sequence similarity 183 member A
Lung	ENSAMEG00000001888	TSHR	thyroid stimulating hormone receptor
Lung	ENSAMEG00000001940	RSPH1	radial spoke head 1 homolog
Lung	ENSAMEG00000002023	TFF1	trefoil factor 1
Lung	ENSAMEG00000002265	METTL7A	methyltransferase like 7A
Lung	ENSAMEG00000002295	SLC22A18	solute carrier family 22 member 18
Lung	ENSAMEG00000002301	RASSF10	Ras association domain family member 10
Lung	ENSAMEG00000002304	--	--
Lung	ENSAMEG00000002317	SFTPB	surfactant protein B
Lung	ENSAMEG00000002492	AGER	advanced glycosylation end-product specific receptor
Lung	ENSAMEG00000002502	KNDC1	kinase non-catalytic C-lobe domain containing 1
Lung	ENSAMEG00000002503	APOBEC4	apolipoprotein B mRNA editing enzyme catalytic polypeptide like 4
Lung	ENSAMEG00000002575	CFAP65	cilia and flagella associated protein 65
Lung	ENSAMEG00000002584	LOC105234745	mucin-17-like
Lung	ENSAMEG00000002619	MAK	male germ cell associated kinase
Lung	ENSAMEG00000002620	ATP6V0D2	ATPase H+ transporting V0 subunit d2

Lung	ENSAMEG00000002626	--	--
Lung	ENSAMEG00000002682	SLC1A1	solute carrier family 1 member 1
Lung	ENSAMEG00000002708	OSR1	odd-skipped related transciption factor 1
Lung	ENSAMEG00000002754	MILR1	mast cell immunoglobulin like receptor 1
Lung	ENSAMEG00000002762	--	--
Lung	ENSAMEG00000002782	IDO1	indoleamine 2,3-dioxygenase 1
Lung	ENSAMEG00000002800	ALOX15B	arachidonate 15-lipoxygenase, type B
Lung	ENSAMEG00000002840	C5orf49	chromosome 5 open reading frame 49
Lung	ENSAMEG00000002893	NKX2-1	NK2 homeobox 1
Lung	ENSAMEG00000003066	PDE1A	phosphodiesterase 1A
Lung	ENSAMEG00000003080	CATIP	ciliogenesis associated TTC17 interacting protein
Lung	ENSAMEG00000003140	PLA2G2E	phospholipase A2 group IIE
Lung	ENSAMEG00000003248	TRPC6	transient receptor potential cation channel subfamily C member 6
Lung	ENSAMEG00000003331	SFTPC	surfactant protein C
Lung	ENSAMEG00000003480	C4BPA	complement component 4 binding protein alpha
Lung	ENSAMEG00000003624	FAM184A	family with sequence similarity 184 member A
Lung	ENSAMEG00000003646	RTKN2	rhotekin 2
Lung	ENSAMEG00000003796	LOC105234588	IQ and AAA domain-containing protein 1
Lung	ENSAMEG00000003811	TM4SF19	transmembrane 4 L six family member 19
Lung	ENSAMEG00000003959	SLC6A4	solute carrier family 6 member 4
Lung	ENSAMEG00000003989	TESPA1	thymocyte expressed, positive selection associated 1
Lung	ENSAMEG00000004046	--	--
Lung	ENSAMEG00000004122	C11orf16	chromosome 11 open reading frame 16
Lung	ENSAMEG00000004298	FAT3	FAT atypical cadherin 3
Lung	ENSAMEG00000004323	C11orf88	chromosome 11 open reading frame 88
Lung	ENSAMEG00000004329	ABCC11	ATP binding cassette subfamily C member 11
Lung	ENSAMEG00000004338	POU2AF1	POU class 2 associating factor 1
Lung	ENSAMEG00000004432	C13orf46	chromosome 13 open reading frame 46
Lung	ENSAMEG00000004479	CD79A	CD79a molecule
Lung	ENSAMEG00000004515	MUC15	mucin 15, cell surface associated
Lung	ENSAMEG00000004557	KIAA1324	KIAA1324
Lung	ENSAMEG00000004693	CLDN11	claudin 11
Lung	ENSAMEG00000004775	CSGALNACT1	chondroitin sulfate N-acetylgalactosaminyltransferase 1
Lung	ENSAMEG00000004777	EDARADD	EDAR associated death domain
Lung	ENSAMEG00000004866	TEX52	testis expressed 52
Lung	ENSAMEG00000004883	IGHM	immunoglobulin heavy constant mu
Lung	ENSAMEG00000005003	CXCL17	C-X-C motif chemokine ligand 17
Lung	ENSAMEG00000005020	PLAC8	placenta specific 8
Lung	ENSAMEG00000005065	PLB1	phospholipase B1
Lung	ENSAMEG00000005188	PTGFRN	prostaglandin F2 receptor inhibitor
Lung	ENSAMEG00000005230	BMP3	bone morphogenetic protein 3
Lung	ENSAMEG00000005284	TFCP2L1	transcription factor CP2 like 1

Lung	ENSAMEG00000005292	ANGPT2	angiopoietin 2
Lung	ENSAMEG00000005528	DTHD1	death domain containing 1
Lung	ENSAMEG00000005665	PIFO	primary cilia formation
Lung	ENSAMEG00000005685	CALHM5	calcium homeostasis modulator family member 5
Lung	ENSAMEG00000005788	CCL13	C-C motif chemokine ligand 13
Lung	ENSAMEG00000005819	FAM162B	family with sequence similarity 162 member B
Lung	ENSAMEG00000005826	AMTN	amelotin
Lung	ENSAMEG00000005851	ROS1	ROS proto-oncogene 1, receptor tyrosine kinase
Lung	ENSAMEG00000005863	JCHAIN	joining chain of multimeric IgA and IgM
Lung	ENSAMEG00000005871	FAR2	fatty acyl-CoA reductase 2
Lung	ENSAMEG00000005892	HOXA6	homeobox A6
Lung	ENSAMEG00000005927	ATP10A	ATPase phospholipid transporting 10A (putative)
Lung	ENSAMEG00000005934	--	--
Lung	ENSAMEG00000006146	CFAP161	cilia and flagella associated protein 161
Lung	ENSAMEG00000006169	CCDC81	coiled-coil domain containing 81
Lung	ENSAMEG00000006173	GRAMD2A	GRAM domain containing 2A
Lung	ENSAMEG00000006234	CLDN18	claudin 18
Lung	ENSAMEG00000006313	--	--
Lung	ENSAMEG00000006446	SULT1C2	sulfotransferase family 1C member 2
Lung	ENSAMEG00000006640	COLEC12	collectin subfamily member 12
Lung	ENSAMEG00000006653	PTTG1	PTTG1 regulator of sister chromatid separation, securin
Lung	ENSAMEG00000006741	NOD2	nucleotide binding oligomerization domain containing 2
Lung	ENSAMEG00000006809	SCUBE1	signal peptide, CUB domain and EGF like domain containing 1
Lung	ENSAMEG00000006864	TCAF2	TRPM8 channel associated factor 2
Lung	ENSAMEG00000006960	AQP8	aquaporin 8
Lung	ENSAMEG00000007039	--	--
Lung	ENSAMEG00000007135	--	--
Lung	ENSAMEG00000007150	--	--
Lung	ENSAMEG00000007198	PLA2G3	phospholipase A2 group III
Lung	ENSAMEG00000007209	INPP5J	inositol polyphosphate-5-phosphatase J
Lung	ENSAMEG00000007288	A3GALT2	alpha 1,3-galactosyltransferase 2
Lung	ENSAMEG00000007300	UPK1B	uroplakin 1B
Lung	ENSAMEG00000007480	SEC14L4	SEC14 like lipid binding 4
Lung	ENSAMEG00000007483	SEC14L3	SEC14 like lipid binding 3
Lung	ENSAMEG00000007517	HAPLN1	hyaluronan and proteoglycan link protein 1
Lung	ENSAMEG00000007545	CDKN2B	cyclin dependent kinase inhibitor 2B
Lung	ENSAMEG00000007593	--	--
Lung	ENSAMEG00000007633	--	--
Lung	ENSAMEG00000007821	DCDC2B	doublecortin domain containing 2B
Lung	ENSAMEG00000007894	CAPS	calcyphosine
Lung	ENSAMEG00000007915	MCTP1	multiple C2 and transmembrane domain containing 1
Lung	ENSAMEG00000007952	SLC22A31	solute carrier family 22 member 31

Lung	ENSAMEG00000008000	DDC	dopa decarboxylase
Lung	ENSAMEG00000008079	--	--
Lung	ENSAMEG00000008174	SPATA18	spermatogenesis associated 18
Lung	ENSAMEG00000008217	--	--
Lung	ENSAMEG00000008258	NKD2	naked cuticle homolog 2
Lung	ENSAMEG00000008427	--	--
Lung	ENSAMEG00000008448	VIPR2	vasoactive intestinal peptide receptor 2
Lung	ENSAMEG00000008534	HOPX	HOP homeobox
Lung	ENSAMEG00000008555	LOC105234723	sciellin
Lung	ENSAMEG00000008599	PKHD1	PKHD1 ciliary IPT domain containing fibrocystin/polyductin
Lung	ENSAMEG00000008662	APOA1	Apolipoprotein A-I
Lung	ENSAMEG00000008683	--	--
Lung	ENSAMEG00000008789	FAM196B	family with sequence similarity 196 member B
Lung	ENSAMEG00000008802	TBX4	T-box 4
Lung	ENSAMEG00000008863	ACSM2B	acyl-CoA synthetase medium chain family member 2B
Lung	ENSAMEG00000008871	FAM216B	family with sequence similarity 216 member B
Lung	ENSAMEG00000008913	--	--
Lung	ENSAMEG00000008921	LOC105242279	thrombospondin type-1 domain-containing protein 7B
Lung	ENSAMEG00000008938	RIBC2	RIB43A domain with coiled-coils 2
Lung	ENSAMEG00000008984	GP2	glycoprotein 2
Lung	ENSAMEG00000009007	LOC100478867	radial spoke head 10 homolog B
Lung	ENSAMEG00000009009	TSPAN19	tetraspanin 19
Lung	ENSAMEG00000009010	BICD1	BICD cargo adaptor 1
Lung	ENSAMEG00000009043	--	--
Lung	ENSAMEG00000009264	C15orf59	chromosome 15 open reading frame 59
Lung	ENSAMEG00000009278	TBXT	T-box transcription factor T
Lung	ENSAMEG00000009292	GDPD2	glycerophosphodiester phosphodiesterase domain containing 2
Lung	ENSAMEG00000009399	P2RX1	purinergic receptor P2X 1
Lung	ENSAMEG00000009440	LOC100472452	cytochrome P450 2D15
Lung	ENSAMEG00000009442	CCNO	cyclin O
Lung	ENSAMEG00000009525	--	--
Lung	ENSAMEG00000009526	ACOXL	acyl-CoA oxidase like
Lung	ENSAMEG00000009530	C8orf34	chromosome 8 open reading frame 34
Lung	ENSAMEG00000009646	NPHP1	nephrocystin 1
Lung	ENSAMEG00000009731	LDLRAD1	low density lipoprotein receptor class A domain containing 1
Lung	ENSAMEG00000009849	CD93	CD93 molecule
Lung	ENSAMEG00000009876	GRID1	glutamate ionotropic receptor delta type subunit 1
Lung	ENSAMEG00000009981	ANGPTL5	angiopoietin like 5
Lung	ENSAMEG00000009985	CEP126	centrosomal protein 126
Lung	ENSAMEG00000010030	PNMT	phenylethanolamine N-methyltransferase
Lung	ENSAMEG00000010062	LOC100477293	annexin A8

Lung	ENSAMEG00000010166	--	--
Lung	ENSAMEG00000010185	--	--
Lung	ENSAMEG00000010189	--	--
Lung	ENSAMEG00000010232	KIAA0319	KIAA0319
Lung	ENSAMEG00000010289	F7	coagulation factor VII
Lung	ENSAMEG00000010294	GPLD1	glycosylphosphatidylinositol specific phospholipase D1
Lung	ENSAMEG00000010479	--	--
Lung	ENSAMEG00000010522	CFAP46	cilia and flagella associated protein 46
Lung	ENSAMEG00000010541	AGR2	anterior gradient 2, protein disulphide isomerase family member
Lung	ENSAMEG00000010687	GSG1	germ cell associated 1
Lung	ENSAMEG00000010738	GPRC5A	G protein-coupled receptor class C group 5 member A
Lung	ENSAMEG00000010782	ST8SIA2	ST8 alpha-N-acetyl-neuraminide alpha-2,8-sialyltransferase 2
Lung	ENSAMEG00000010788	SPEF1	sperm flagellar 1
Lung	ENSAMEG00000010815	--	--
Lung	ENSAMEG00000010825	SPTBN2	spectrin beta, non-erythrocytic 2
Lung	ENSAMEG00000010938	NRG2	neuregulin 2
Lung	ENSAMEG00000010959	FGFR2	fibroblast growth factor receptor 2
Lung	ENSAMEG00000010989	CCL26	C-C motif chemokine ligand 26
Lung	ENSAMEG00000011029	SOX2	SRY-box 2
Lung	ENSAMEG00000011032	CCDC39	coiled-coil domain containing 39
Lung	ENSAMEG00000011038	LRRC74B	leucine rich repeat containing 74B
Lung	ENSAMEG00000011045	TM4SF18	transmembrane 4 L six family member 18
Lung	ENSAMEG00000011060	--	--
Lung	ENSAMEG00000011277	CUNH4orf19	chromosome unknown C4orf19 homolog
Lung	ENSAMEG00000011375	CRLF1	cytokine receptor like factor 1
Lung	ENSAMEG00000011494	FBP1	fructose-bisphosphatase 1
Lung	ENSAMEG00000011512	NYAP2	neuronal tyrosine-phosphorylated phosphoinositide-3-kinase adaptor 2
Lung	ENSAMEG00000011544	--	--
Lung	ENSAMEG00000011666	ZMYND10	zinc finger MYND-type containing 10
Lung	ENSAMEG00000011683	--	--
Lung	ENSAMEG00000011776	TCTE1	t-complex-associated-testis-expressed 1
Lung	ENSAMEG00000011844	SLC25A34	solute carrier family 25 member 34
Lung	ENSAMEG00000011870	SLC38A3	solute carrier family 38 member 3
Lung	ENSAMEG00000011898	CFH	complement factor H
Lung	ENSAMEG00000011913	RIMS3	regulating synaptic membrane exocytosis 3
Lung	ENSAMEG00000011916	CDH2	cadherin 2
Lung	ENSAMEG00000011960	LOC100465091	cytochrome P450 2A13-like
Lung	ENSAMEG00000012034	VWA5A	von Willebrand factor A domain containing 5A
Lung	ENSAMEG00000012233	VLDLR	very low density lipoprotein receptor
Lung	ENSAMEG00000012290	CDHR4	cadherin related family member 4

Lung	ENSAMEG00000012467	PRX	periaxin
Lung	ENSAMEG00000012556	LOC100474871	alpha-1-acid glycoprotein
Lung	ENSAMEG00000012696	C20orf85	chromosome 20 open reading frame 85
Lung	ENSAMEG00000012956	SLC44A4	solute carrier family 44 member 4
Lung	ENSAMEG00000012974	--	--
Lung	ENSAMEG00000012994	--	--
Lung	ENSAMEG00000013003	SFTPD	surfactant protein D
Lung	ENSAMEG00000013016	LOC100482856	pulmonary surfactant-associated protein A
Lung	ENSAMEG00000013142	--	--
Lung	ENSAMEG00000013166	CD3E	CD3e molecule
Lung	ENSAMEG00000013243	NAPSA	napsin A aspartic peptidase
Lung	ENSAMEG00000013319	CDHR3	cadherin related family member 3
Lung	ENSAMEG00000013403	CLIC3	chloride intracellular channel 3
Lung	ENSAMEG00000013419	--	--
Lung	ENSAMEG00000013508	VWA3B	von Willebrand factor A domain containing 3B
Lung	ENSAMEG00000013516	OMD	osteomodulin
Lung	ENSAMEG00000013530	UCMA	upper zone of growth plate and cartilage matrix associated
Lung	ENSAMEG00000013547	--	--
Lung	ENSAMEG00000013757	SLC24A4	solute carrier family 24 member 4
Lung	ENSAMEG00000013814	TSPAN1	tetraspanin 1
Lung	ENSAMEG00000013822	RBP4	retinol binding protein 4
Lung	ENSAMEG00000013869	A2M	alpha-2-macroglobulin
Lung	ENSAMEG00000013885	LRRC23	leucine rich repeat containing 23
Lung	ENSAMEG00000014075	CYP4B1	cytochrome P450 family 4 subfamily B member 1
Lung	ENSAMEG00000014219	AQP4	aquaporin 4
Lung	ENSAMEG00000014281	--	--
Lung	ENSAMEG00000014282	IGLON5	IgLON family member 5
Lung	ENSAMEG00000014494	KCNA7	potassium voltage-gated channel subfamily A member 7
Lung	ENSAMEG00000014501	LHB	luteinizing hormone beta polypeptide (LHB)
Lung	ENSAMEG00000014666	TPO	thyroid peroxidase
Lung	ENSAMEG00000014671	C9orf24	chromosome 9 open reading frame 24
Lung	ENSAMEG00000014693	NPY1R	neuropeptide Y receptor Y1
Lung	ENSAMEG00000014699	NPY5R	neuropeptide Y receptor Y5
Lung	ENSAMEG00000014750	GRIA3	glutamate ionotropic receptor AMPA type subunit 3
Lung	ENSAMEG00000014774	GAS2	growth arrest specific 2
Lung	ENSAMEG00000014824	KANK4	KN motif and ankyrin repeat domains 4
Lung	ENSAMEG00000014850	ENPEP	glutamyl aminopeptidase
Lung	ENSAMEG00000014938	TC2N	tandem C2 domains, nuclear
Lung	ENSAMEG00000015118	INSC	INSC, spindle orientation adaptor protein
Lung	ENSAMEG00000015132	--	--
Lung	ENSAMEG00000015141	CCDC40	coiled-coil domain containing 40
Lung	ENSAMEG00000015212	ALPK2	alpha kinase 2
Lung	ENSAMEG00000015296	SNTN	sentan, cilia apical structure protein

Lung	ENSAMEG00000015366	--	--
Lung	ENSAMEG00000015481	SUSD4	sushi domain containing 4
Lung	ENSAMEG00000015616	CD7	CD7 molecule
Lung	ENSAMEG00000015682	--	--
Lung	ENSAMEG00000015711	PRDM6	PR/SET domain 6
Lung	ENSAMEG00000015799	LRRK2	leucine rich repeat kinase 2
Lung	ENSAMEG00000015997	LAMP3	lysosomal associated membrane protein 3
Lung	ENSAMEG00000016264	STOML3	stomatin like 3
Lung	ENSAMEG00000016308	WNT3A	Wnt family member 3A
Lung	ENSAMEG00000016313	PLA2G4D	phospholipase A2 group IVD
Lung	ENSAMEG00000016338	PLA2G4E	phospholipase A2 group IVE
Lung	ENSAMEG00000016356	CLEC10A	C-type lectin domain containing 10A
Lung	ENSAMEG00000016488	SLC6A2	solute carrier family 6 member 2
Lung	ENSAMEG00000016510	GFRA3	GDNF family receptor alpha 3
Lung	ENSAMEG00000016548	FOXN4	forkhead box N4
Lung	ENSAMEG00000016613	PPP1R32	protein phosphatase 1 regulatory subunit 32
Lung	ENSAMEG00000016719	SEMA3E	semaphorin 3E
Lung	ENSAMEG00000016732	ETV5	ETS variant 5
Lung	ENSAMEG00000016881	TMTC2	transmembrane and tetratricopeptide repeat containing 2
Lung	ENSAMEG00000016897	EPHB3	EPH receptor B3
Lung	ENSAMEG00000016999	CHST8	carbohydrate sulfotransferase 8
Lung	ENSAMEG00000017015	--	--
Lung	ENSAMEG00000017168	PGM2L1	phosphoglucomutase 2 like 1
Lung	ENSAMEG00000017190	P4HA3	prolyl 4-hydroxylase subunit alpha 3
Lung	ENSAMEG00000017238	HSF4	heat shock transcription factor 4
Lung	ENSAMEG00000017246	DDAH1	dimethylarginine dimethylaminohydrolase 1
Lung	ENSAMEG00000017265	WDR63	WD repeat domain 63
Lung	ENSAMEG00000017389	HSD11B2	hydroxysteroid 11-beta dehydrogenase 2
Lung	ENSAMEG00000017432	--	--
Lung	ENSAMEG00000017435	ARHGEF38	Rho guanine nucleotide exchange factor 38
Lung	ENSAMEG00000017793	DLEC1	deleted in lung and esophageal cancer 1
Lung	ENSAMEG00000017827	PALD1	phosphatase domain containing, paladin 1
Lung	ENSAMEG00000017862	ABCA3	ATP binding cassette subfamily A member 3
Lung	ENSAMEG00000017954	ATP6V0A4	ATPase H+ transporting V0 subunit a4
Lung	ENSAMEG00000018020	C9orf152	chromosome 9 open reading frame 152
Lung	ENSAMEG00000018041	GSTO2	glutathione S-transferase omega 2
Lung	ENSAMEG00000018105	SLC34A2	solute carrier family 34 member 2
Lung	ENSAMEG00000018118	TTPA	alpha tocopherol transfer protein
Lung	ENSAMEG00000018130	--	--
Lung	ENSAMEG00000018266	FOXF1	forkhead box F1
Lung	ENSAMEG00000018312	CD300LG	CD300 molecule like family member g
Lung	ENSAMEG00000018389	HIGD1B	HIG1 hypoxia inducible domain family member 1B
Lung	ENSAMEG00000018442	FHDC1	FH2 domain containing 1

Lung	ENSAMEG00000018799	--	--
Lung	ENSAMEG00000018847	LOC100482402	olfactory receptor 51L1
Lung	ENSAMEG00000019453	LOC100477677	carbohydrate sulfotransferase 6
Lung	ENSAMEG00000019512	--	--
Lung	ENSAMEG00000019577	LOC100477491	olfactory receptor 1F1
Lung	ENSAMEG00000019607	--	--
Lung	ENSAMEG00000019729	FPR2	formyl peptide receptor 2
Lung	ENSAMEG00000019739	FLRT3	fibronectin leucine rich transmembrane protein 3
Lung	ENSAMEG00000019773	--	--
Lung	ENSAMEG00000019810	LOC100469231	neuroendocrine secretory protein 55
Lung	ENSAMEG00000020025	APLNR	apelin receptor
Lung	Novel00020	--	--
Lung	Novel00064	--	PREDICTED: alpha-1B adrenergic receptor
Lung	Novel00066	--	--
Lung	Novel00082	--	hypothetical protein PANDA_000426, partial
Lung	Novel00113	--	--
Lung	Novel00139	--	--
Lung	Novel00140	--	hypothetical protein M91_05014, partial
Lung	Novel00207	--	rCG65845
Lung	Novel00269	--	hypothetical protein PANDA_001238, partial
Lung	Novel00281	--	Histone acetyltransferase MYST3
Lung	Novel00283	--	--
Lung	Novel00387	--	perforin
Lung	Novel00436	--	--
Lung	Novel00460	--	unnamed protein product
Lung	Novel00601	--	--
Lung	Novel00610	--	PREDICTED: spermatogenesis-associated serine-rich protein 1
Lung	Novel00660	--	PREDICTED: LOW QUALITY PROTEIN: papilin
Lung	Novel00670	--	hypothetical protein PANDA_002650, partial
Lung	Novel00688	--	PREDICTED: transmembrane protein 2 isoform X2
Lung	Novel00693	--	--
Lung	Novel00727	--	hypothetical protein EGK_05727, partial
Lung	Novel00780	--	--
Lung	Novel00801	--	--
Lung	Novel00814	--	PREDICTED: cyclin-related protein FAM58A, partial
Lung	Novel00833	--	--
Lung	Novel01080	--	endonuclease/reverse transcriptase
Lung	Novel01105	--	--
Lung	Novel01152	--	--
Lung	Novel01196	--	--
Lung	Novel01234	--	PREDICTED: transmembrane and TPR repeat-containing protein 1 isoform X6

Lung	Novel01296	--	--
Lung	Novel01299	--	--
Lung	Novel01330	--	hypothetical protein BRAFLDRAFT_124569
Lung	Novel01419	--	--
Lung	Novel01480	--	hypothetical protein M91_16134, partial
Lung	Novel01485	--	--
Lung	Novel01486	--	--
Lung	Novel01492	--	--
Lung	Novel01495	--	--
Lung	Novel01540	--	--
Lung	Novel01595	--	--
Lung	Novel01634	--	--
Lung	Novel01636	--	PREDICTED: homeodomain-only protein isoform X1
Lung	Novel01637	--	hypothetical protein M91_18560, partial
Lung	Novel01638	--	--
Lung	Novel01693	--	hypothetical protein EGM_15239, partial
Lung	Novel01709	--	--
Lung	Novel01711	--	PREDICTED: protein N-terminal asparagine amidohydrolase isoform 1
Lung	Novel01715	--	--
Lung	Novel01834	--	--
Lung	Novel01854	--	--
Lung	Novel01889	--	--
Lung	Novel02130	--	hypothetical protein M91_14620
Lung	Novel02146	--	hypothetical protein M91_20222, partial
Lung	Novel02191	--	--
Lung	Novel02323	--	--
Lung	Novel02344	--	PREDICTED: T-box transcription factor TBX1
Lung	Novel02444	--	PREDICTED: granulocyte-macrophage colony-stimulating factor receptor subunit alpha-like
Lung	Novel02477	--	--
Lung	Novel02527	--	--
Lung	Novel02539	--	--
Lung	Novel02594	--	--
Lung	Novel02602	--	PREDICTED: adaptin ear-binding coat-associated protein 1
Lung	Novel02625	--	--
Lung	Novel02646	--	PREDICTED: LOW QUALITY PROTEIN: protein phosphatase 1 regulatory subunit 3A
Lung	Novel02648	--	hypothetical protein M91_14620
Lung	Novel02695	--	Retrovirus-related Pol polyprotein LINE-1
Lung	Novel02723	--	PREDICTED: LOW QUALITY PROTEIN: transmembrane protein 200C
Lung	Novel02724	--	putative p150

Lung	Novel02727	--	--
Lung	Novel02742	--	--
Lung	Novel02751	--	--
Lung	Novel02778	--	--
Lung	Novel02904	--	hypothetical protein PANDA_011658, partial
Lung	Novel02931	--	hCG1813636
Lung	Novel02943	--	--
Lung	Novel02947	--	hCG1820541, partial
Lung	Novel02993	--	--
Lung	Novel02996	--	--
Lung	Novel03051	--	--
Lung	Novel03130	--	PREDICTED: thioredoxin domain-containing protein 6 isoform X3
Lung	Novel03271	--	--
Lung	Novel03466	--	PREDICTED: T-box transcription factor TBX4
Lung	Novel03507	--	--
Lung	Novel03542	--	PREDICTED: killer cell lectin-like receptor 2
Lung	Novel03583	--	PREDICTED: killer cell lectin-like receptor 2
Lung	Novel03612	--	--
Lung	Novel03615	--	--
Lung	Novel03748	--	60S ribosomal protein L32-like protein
Lung	Novel03749	--	--
Lung	Novel03779	--	PREDICTED: sciellin
Lung	Novel03823	--	PREDICTED: LOW QUALITY PROTEIN: putative histone-lysine N-methyltransferase PRDM6, partial
Lung	Novel03933	--	--
Lung	Novel03958	--	--
Lung	Novel03963	--	RIKEN cDNA 5330421F07, partial
Lung	Novel04021	--	--
Lung	Novel04123	--	--
Lung	Novel04135	--	PREDICTED: collagen alpha-1(II) chain-like
Lung	Novel04198	--	LINE-1 element ORF2 (predicted)
Lung	Novel04201	--	unnamed protein product
Lung	Novel04296	--	PREDICTED: LOW QUALITY PROTEIN: proto-oncogene tyrosine-protein kinase ROS-like
Lung	Novel04309	--	hypothetical protein H671_1g0331
Lung	Novel04317	--	--
Lung	Novel04441	--	PREDICTED: uncharacterized protein LOC102160087
Lung	Novel04485	--	--
Lung	Novel04529	--	--
Lung	Novel04546	--	ORF4
Lung	Novel04723	--	endonuclease/reverse transcriptase
Lung	Novel04727	--	hypothetical protein PAL_GLEAN10005188

Lung	Novel04728	--	--
Lung	Novel04772	--	--
Lung	Novel04785	--	PREDICTED: surfactant-associated protein 2
Lung	Novel04813	--	PREDICTED: dynein heavy chain 9, axonemal
Lung	Novel04861	--	--
Lung	Novel04862	--	--
Lung	Novel04891	--	--
Lung	Novel04997	--	--
Lung	Novel05003	--	--
Lung	Novel05019	--	--
Lung	Novel05123	--	PREDICTED: uncharacterized protein LOC105242333
Lung	Novel05140	--	PREDICTED: acyl-coenzyme A oxidase-like protein, partial
Lung	Novel05184	--	--
Lung	Novel05228	--	transposase
Lung	Novel05240	--	PREDICTED: LOW QUALITY PROTEIN: gamma-aminobutyric acid type B receptor subunit 1
Lung	Novel05290	--	PREDICTED: LOW QUALITY PROTEIN: pregnancy zone protein-like, partial
Lung	Novel05332	--	--
Lung	Novel05360	--	PREDICTED: uncharacterized protein LOC102471881 isoform X2
Lung	Novel05372	--	hypothetical protein M91_17975
Lung	Novel05383	--	--
Lung	Novel05444	--	PREDICTED: sphingosine-1-phosphate phosphatase 2-like
Lung	Novel05575	--	--
Lung	Novel05619	--	PREDICTED: carcinoembryonic antigen-related cell adhesion molecule 21-like isoform X1
Lung	Novel05630	--	--
Lung	Novel05683	--	PREDICTED: putative 60S ribosomal protein L37a
Lung	Novel05700	--	hypothetical protein M91_07955
Lung	Novel05701	--	--
Lung	Novel05713	--	--
Lung	Novel05741	--	--
Lung	Novel05756	--	hypothetical protein PANDA_022245, partial
Lung	Novel05789	--	hypothetical protein PANDA_022245, partial
Lung	Novel05889	--	PREDICTED: leucine-rich repeat-containing protein-like
Lung	Novel05913	--	hypothetical protein PANDA_022433, partial
Lung	Novel05950	--	PREDICTED: putative solute carrier family 22 member 31 isoform X3
Lung	Novel06033	--	PREDICTED: cadherin-15-like
Kidney	ENSAMEG00000000008	SLC13A3	solute carrier family 13 member 3
Kidney	ENSAMEG00000000025	CCDC148	coiled-coil domain containing 148
Kidney	ENSAMEG00000000030	PDZD3	PDZ domain containing 3

Kidney	ENSAMEG00000000089	SFRP5	secreted frizzled related protein 5
Kidney	ENSAMEG00000000107	HOGA1	4-hydroxy-2-oxoglutarate aldolase 1
Kidney	ENSAMEG00000000246	CLRN3	clarin 3
Kidney	ENSAMEG00000000277	FGGY	FGGY carbohydrate kinase domain containing
Kidney	ENSAMEG00000000342	CYP2J2	cytochrome P450 family 2 subfamily J member 2
Kidney	ENSAMEG00000000405	UNC13C	unc-13 homolog C
Kidney	ENSAMEG00000000519	OSR2	odd-skipped related transciption factor 2
Kidney	ENSAMEG00000000560	ZMYND12	zinc finger MYND-type containing 12
Kidney	ENSAMEG00000000609	MATN2	matrilin 2
Kidney	ENSAMEG00000000614	GUCA2A	guanylate cyclase activator 2A
Kidney	ENSAMEG00000000616	GUCA2B	guanylate cyclase activator 2B
Kidney	ENSAMEG00000000673	HOXC10	homeobox C10
Kidney	ENSAMEG00000000678	HOXC9	homeobox C9
Kidney	ENSAMEG00000000683	HOXC8	homeobox C8
Kidney	ENSAMEG00000000686	HOXC6	homeobox C6
Kidney	ENSAMEG00000000691	HOXC5	homeobox C5
Kidney	ENSAMEG00000000693	NPHS1	NPHS1, nephrin
Kidney	ENSAMEG00000000697	HOXC4	homeobox C4
Kidney	ENSAMEG00000000790	SCUBE3	signal peptide, CUB domain and EGF like domain containing 3
Kidney	ENSAMEG00000000843	MASP1	mannan binding lectin serine peptidase 1
Kidney	ENSAMEG00000000867	C1QL4	complement C1q like 4
Kidney	ENSAMEG00000000927	REN	renin
Kidney	ENSAMEG00000000942	MLANA	melan-A
Kidney	ENSAMEG00000000982	GOLT1A	golgi transport 1A
Kidney	ENSAMEG00000000994	KNG1	kininogen 1
Kidney	ENSAMEG00000001013	HRG	histidine rich glycoprotein
Kidney	ENSAMEG00000001033	MTTP	microsomal triglyceride transfer protein
Kidney	ENSAMEG00000001045	C5	complement C5
Kidney	ENSAMEG00000001078	LOC100467242	double C2-like domain-containing protein gamma
Kidney	ENSAMEG00000001087	AQP6	aquaporin 6
Kidney	ENSAMEG00000001285	--	--
Kidney	ENSAMEG00000001406	CCDC160	coiled-coil domain containing 160
Kidney	ENSAMEG00000001422	HHLA2	HERV-H LTR-associating 2
Kidney	ENSAMEG00000001432	GPC4	glypican 4
Kidney	ENSAMEG00000001438	--	--
Kidney	ENSAMEG00000001461	NLGN1	neuroligin 1
Kidney	ENSAMEG00000001516	FOXI1	forkhead box I1
Kidney	ENSAMEG00000001556	ADGRF1	adhesion G protein-coupled receptor F1
Kidney	ENSAMEG00000001643	SLC30A3	solute carrier family 30 member 3
Kidney	ENSAMEG00000001683	KRT7	keratin 7
Kidney	ENSAMEG00000001809	F2RL1	F2R like trypsin receptor 1
Kidney	ENSAMEG00000001828	HOXD1	homeobox D1

Kidney	ENSAMEG00000001830	HOXD3	homeobox D3
Kidney	ENSAMEG00000001837	HOXD4	homeobox D4
Kidney	ENSAMEG00000001838	HOXD8	homeobox D8
Kidney	ENSAMEG00000001843	--	--
Kidney	ENSAMEG00000001849	HOXD10	homeobox D10
Kidney	ENSAMEG00000001856	HOXD11	homeobox D11
Kidney	ENSAMEG00000001883	CER1	cerberus 1, DAN family BMP antagonist
Kidney	ENSAMEG00000001889	FREM1	FRAS1 related extracellular matrix 1
Kidney	ENSAMEG00000001894	SLC12A1	solute carrier family 12 member 1
Kidney	ENSAMEG00000001973	USP43	ubiquitin specific peptidase 43
Kidney	ENSAMEG00000001989	TMPRSS3	transmembrane serine protease 3
Kidney	ENSAMEG00000002088	DCDC2	doublecortin domain containing 2
Kidney	ENSAMEG00000002100	LONRF3	LON peptidase N-terminal domain and ring finger 3
Kidney	ENSAMEG00000002115	AMDHD1	amidohydrolase domain containing 1
Kidney	ENSAMEG00000002202	CLDN19	claudin 19
Kidney	ENSAMEG00000002230	SLC35F2	solute carrier family 35 member F2
Kidney	ENSAMEG00000002328	CA12	carbonic anhydrase 12
Kidney	ENSAMEG00000002371	RALYL	RALY RNA binding protein like
Kidney	ENSAMEG00000002423	CCDC198	coiled-coil domain containing 198
Kidney	ENSAMEG00000002538	SLC23A3	solute carrier family 23 member 3
Kidney	ENSAMEG00000002558	TFAP2A	transcription factor AP-2 alpha
Kidney	ENSAMEG00000002567	CA2	carbonic anhydrase 2
Kidney	ENSAMEG00000002582	RYR2	ryanodine receptor 2
Kidney	ENSAMEG00000002620	ATP6V0D2	ATPase H+ transporting V0 subunit d2
Kidney	ENSAMEG00000002691	ELOVL2	ELOVL fatty acid elongase 2
Kidney	ENSAMEG00000002751	RIPPLY1	ripply transcriptional repressor 1
Kidney	ENSAMEG00000002772	SLC51B	solute carrier family 51 beta subunit
Kidney	ENSAMEG00000002832	SLC16A2	solute carrier family 16 member 2
Kidney	ENSAMEG00000002869	UCP1	uncoupling protein 1
Kidney	ENSAMEG00000002890	MIOX	myo-inositol oxygenase
Kidney	ENSAMEG00000002970	LOC100478661	ras-associated and pleckstrin homology domains-containing protein 1
Kidney	ENSAMEG00000002977	MAPK4	mitogen-activated protein kinase 4
Kidney	ENSAMEG00000002998	VIL1	villin 1
Kidney	ENSAMEG00000003069	NDNF	neuron derived neurotrophic factor
Kidney	ENSAMEG00000003156	SPP1	secreted phosphoprotein 1
Kidney	ENSAMEG00000003181	USH1C	USH1 protein network component harmonin
Kidney	ENSAMEG00000003324	TTC23L	tetratricopeptide repeat domain 23 like
Kidney	ENSAMEG00000003417	TNNT1	troponin T1, slow skeletal type
Kidney	ENSAMEG00000003459	DMTN	dematin actin binding protein
Kidney	ENSAMEG00000003513	RAB15	RAB15, member RAS oncogene family
Kidney	ENSAMEG00000003602	RAB3C	RAB3C, member RAS oncogene family
Kidney	ENSAMEG00000003646	RTKN2	rhotekin 2

Kidney	ENSAMEG00000003734	FXYD2	FXYD domain containing ion transport regulator 2
Kidney	ENSAMEG00000003870	TMEM52B	transmembrane protein 52B
Kidney	ENSAMEG00000003893	ADGRG7	adhesion G protein-coupled receptor G7
Kidney	ENSAMEG00000003930	TMIGD1	transmembrane and immunoglobulin domain containing 1
Kidney	ENSAMEG00000004034	--	--
Kidney	ENSAMEG00000004040	MUC20	mucin 20, cell surface associated
Kidney	ENSAMEG00000004095	STAC2	SH3 and cysteine rich domain 2
Kidney	ENSAMEG00000004261	LOC109489886	multidrug and toxin extrusion protein 2
Kidney	ENSAMEG00000004329	ABCC11	ATP binding cassette subfamily C member 11
Kidney	ENSAMEG00000004335	SLC47A2	solute carrier family 47 member 2
Kidney	ENSAMEG00000004350	SLC5A12	solute carrier family 5 member 12
Kidney	ENSAMEG00000004372	OVCH2	ovochymase 2 (gene/pseudogene)
Kidney	ENSAMEG00000004389	ALDH3A1	aldehyde dehydrogenase 3 family member A1
Kidney	ENSAMEG00000004466	SLC13A1	solute carrier family 13 member 1
Kidney	ENSAMEG00000004515	MUC15	mucin 15, cell surface associated
Kidney	ENSAMEG00000004560	TMC4	transmembrane channel like 4
Kidney	ENSAMEG00000004566	SLC5A10	solute carrier family 5 member 10
Kidney	ENSAMEG00000004574	EFCAB12	EF-hand calcium binding domain 12
Kidney	ENSAMEG00000004582	CNDP1	carnosine dipeptidase 1
Kidney	ENSAMEG00000004593	LYPD6B	LY6/PLAUR domain containing 6B
Kidney	ENSAMEG00000004675	SHMT1	serine hydroxymethyltransferase 1
Kidney	ENSAMEG00000004767	HGD	homogentisate 1,2-dioxygenase
Kidney	ENSAMEG00000004778	LOC100474931	C-type lectin domain family 18 member A
Kidney	ENSAMEG00000004783	SYPL2	synaptophysin like 2
Kidney	ENSAMEG00000004937	ANGPTL4	angiopoietin like 4
Kidney	ENSAMEG00000004949	SLC13A2	solute carrier family 13 member 2
Kidney	ENSAMEG00000004971	HOXB6	homeobox B6
Kidney	ENSAMEG00000004977	HOXB7	homeobox B7
Kidney	ENSAMEG00000004982	HOXB8	homeobox B8
Kidney	ENSAMEG00000004990	HOXB9	homeobox B9
Kidney	ENSAMEG00000005003	CXCL17	C-X-C motif chemokine ligand 17
Kidney	ENSAMEG00000005030	SPOCK3	SPARC (osteonectin), cwcv and kazal like domains proteoglycan 3
Kidney	ENSAMEG00000005157	SCG5	secretogranin V
Kidney	ENSAMEG00000005199	PRKG2	protein kinase, cGMP-dependent, type II
Kidney	ENSAMEG00000005229	SLC6A17	solute carrier family 6 member 17
Kidney	ENSAMEG00000005259	FOLH1	folate hydrolase 1
Kidney	ENSAMEG00000005284	TFCP2L1	transcription factor CP2 like 1
Kidney	ENSAMEG00000005463	FRAS1	Fraser extracellular matrix complex subunit 1
Kidney	ENSAMEG00000005554	LRRC19	leucine rich repeat containing 19
Kidney	ENSAMEG00000005588	SIM2	single-minded family bHLH transcription factor 2
Kidney	ENSAMEG00000005615	--	--
Kidney	ENSAMEG00000005696	BHMT	betaine--homocysteine S-methyltransferase

Kidney	ENSAMEG00000005823	GPRC6A	G protein-coupled receptor class C group 6 member A
Kidney	ENSAMEG00000005833	CITED1	Cbp/p300 interacting transactivator with Glu/Asp rich carboxy-terminal domain 1
Kidney	ENSAMEG00000005872	EVX1	even-skipped homeobox 1
Kidney	ENSAMEG00000005879	HOXA11	homeobox A11
Kidney	ENSAMEG00000005883	HOXA10	homeobox A10
Kidney	ENSAMEG00000005884	GADL1	glutamate decarboxylase like 1
Kidney	ENSAMEG00000005885	HOXA9	homeobox A9
Kidney	ENSAMEG00000005889	SLC3A1	solute carrier family 3 member 1
Kidney	ENSAMEG00000005891	HOXA7	homeobox A7
Kidney	ENSAMEG00000005892	HOXA6	homeobox A6
Kidney	ENSAMEG00000005900	TRPV4	transient receptor potential cation channel subfamily V member 4
Kidney	ENSAMEG00000005926	BMPR1B	bone morphogenetic protein receptor type 1B
Kidney	ENSAMEG00000005964	CDH6	cadherin 6
Kidney	ENSAMEG00000005972	SLC4A4	solute carrier family 4 member 4
Kidney	ENSAMEG00000005988	CRYM	crystallin mu
Kidney	ENSAMEG00000005991	SLC22A8	solute carrier family 22 member 8
Kidney	ENSAMEG00000006012	SLC22A6	solute carrier family 22 member 6
Kidney	ENSAMEG00000006080	GC	GC, vitamin D binding protein
Kidney	ENSAMEG00000006222	ACSM3	acyl-CoA synthetase medium chain family member 3
Kidney	ENSAMEG00000006310	KCNK12	potassium two pore domain channel subfamily K member 12
Kidney	ENSAMEG00000006404	PAH	phenylalanine hydroxylase
Kidney	ENSAMEG00000006408	CFAP100	cilia and flagella associated protein 100
Kidney	ENSAMEG00000006446	SULT1C2	sulfotransferase family 1C member 2
Kidney	ENSAMEG00000006457	NECAB2	N-terminal EF-hand calcium binding protein 2
Kidney	ENSAMEG00000006473	TRPV5	transient receptor potential cation channel subfamily V member 5
Kidney	ENSAMEG00000006494	GBA3	glucosylceramidase beta 3 (gene/pseudogene)
Kidney	ENSAMEG00000006694	PNPLA3	patatin like phospholipase domain containing 3
Kidney	ENSAMEG00000006743	AQP1	aquaporin 1 (Colton blood group)
Kidney	ENSAMEG00000006807	--	--
Kidney	ENSAMEG00000006995	SLC26A7	solute carrier family 26 member 7
Kidney	ENSAMEG00000007018	ABCC2	ATP binding cassette subfamily C member 2
Kidney	ENSAMEG00000007057	TMEM174	transmembrane protein 174
Kidney	ENSAMEG00000007062	TMEM171	transmembrane protein 171
Kidney	ENSAMEG00000007091	CDHR2	cadherin related family member 2
Kidney	ENSAMEG00000007125	PRR35	proline rich 35
Kidney	ENSAMEG00000007150	--	--
Kidney	ENSAMEG00000007238	SMTNL2	smoothelin like 2
Kidney	ENSAMEG00000007244	GGT6	gamma-glutamyltransferase 6
Kidney	ENSAMEG00000007288	A3GALT2	alpha 1,3-galactosyltransferase 2

Kidney	ENSAMEG00000007361	OSBP2	oxysterol binding protein 2
Kidney	ENSAMEG00000007593	--	--
Kidney	ENSAMEG00000007809	SERPINF1	serpin family F member 1
Kidney	ENSAMEG00000007857	SERPINF2	serpin family F member 2
Kidney	ENSAMEG00000007874	--	--
Kidney	ENSAMEG00000008083	TMEM252	transmembrane protein 252
Kidney	ENSAMEG00000008196	CA4	carbonic anhydrase 4
Kidney	ENSAMEG00000008282	ITIH1	inter-alpha-trypsin inhibitor heavy chain 1
Kidney	ENSAMEG00000008305	LHX1	LIM homeobox 1
Kidney	ENSAMEG00000008369	ENPP6	ectonucleotide pyrophosphatase/phosphodiesterase 6
Kidney	ENSAMEG00000008457	--	--
Kidney	ENSAMEG00000008518	SCTR	secretin receptor
Kidney	ENSAMEG00000008599	PKHD1	PKHD1 ciliary IPT domain containing fibrocystin/polyductin
Kidney	ENSAMEG00000008667	--	--
Kidney	ENSAMEG00000008675	--	--
Kidney	ENSAMEG00000008680	TFAP2B	transcription factor AP-2 beta
Kidney	ENSAMEG00000008758	--	--
Kidney	ENSAMEG00000008769	ASB5	ankyrin repeat and SOCS box containing 5
Kidney	ENSAMEG00000008786	DUSP9	dual specificity phosphatase 9
Kidney	ENSAMEG00000008863	ACSM2B	acyl-CoA synthetase medium chain family member 2B
Kidney	ENSAMEG00000008967	UMOD	uromodulin
Kidney	ENSAMEG00000008984	GP2	glycoprotein 2
Kidney	ENSAMEG00000009006	STK31	serine/threonine kinase 31
Kidney	ENSAMEG00000009066	ASPA	aspartoacylase
Kidney	ENSAMEG00000009123	STRA6	stimulated by retinoic acid 6
Kidney	ENSAMEG00000009140	RAB3B	RAB3B, member RAS oncogene family
Kidney	ENSAMEG00000009252	--	--
Kidney	ENSAMEG00000009338	KL	klotho
Kidney	ENSAMEG00000009432	BSND	barttin CLCNK type accessory beta subunit
Kidney	ENSAMEG00000009440	LOC100472452	cytochrome P450 2D15
Kidney	ENSAMEG00000009520	--	--
Kidney	ENSAMEG00000009527	CA9	carbonic anhydrase 9
Kidney	ENSAMEG00000009531	MCCD1	mitochondrial coiled-coil domain 1
Kidney	ENSAMEG00000009559	ALKAL2	ALK and LTK ligand 2
Kidney	ENSAMEG00000009588	--	--
Kidney	ENSAMEG00000009612	CLEC3A	C-type lectin domain family 3 member A
Kidney	ENSAMEG00000009628	VAT1L	vesicle amine transport 1 like
Kidney	ENSAMEG00000009638	TPD52L1	tumor protein D52 like 1
Kidney	ENSAMEG00000009675	FBXO2	F-box protein 2
Kidney	ENSAMEG00000009712	FBXO44	F-box protein 44
Kidney	ENSAMEG00000009729	MAL	mal, T cell differentiation protein
Kidney	ENSAMEG00000009798	PROM2	prominin 2
Kidney	ENSAMEG00000009826	SCIN	scinderin

Kidney	ENSAMEG00000009840	CDKL1	cyclin dependent kinase like 1
Kidney	ENSAMEG00000009861	SLC17A3	solute carrier family 17 member 3
Kidney	ENSAMEG00000009915	SLC17A1	solute carrier family 17 member 1
Kidney	ENSAMEG00000009948	SLC17A4	solute carrier family 17 member 4
Kidney	ENSAMEG00000010023	MAP7	microtubule associated protein 7
Kidney	ENSAMEG00000010068	MMP7	matrix metallopeptidase 7
Kidney	ENSAMEG00000010239	ABCC4	ATP binding cassette subfamily C member 4
Kidney	ENSAMEG00000010289	F7	coagulation factor VII
Kidney	ENSAMEG00000010306	DAPL1	death associated protein like 1
Kidney	ENSAMEG00000010356	SLC6A19	solute carrier family 6 member 19
Kidney	ENSAMEG00000010360	PTER	phosphotriesterase related
Kidney	ENSAMEG00000010366	SLC30A2	solute carrier family 30 member 2
Kidney	ENSAMEG00000010368	CUBN	cubilin
Kidney	ENSAMEG00000010388	S100G	S100 calcium binding protein G
Kidney	ENSAMEG00000010403	SLC6A18	solute carrier family 6 member 18
Kidney	ENSAMEG00000010567	RGL3	ral guanine nucleotide dissociation stimulator like 3
Kidney	ENSAMEG00000010585	--	--
Kidney	ENSAMEG00000010632	CDHR5	cadherin related family member 5
Kidney	ENSAMEG00000010654	LRP2	LDL receptor related protein 2
Kidney	ENSAMEG00000010662	PAX8	paired box 8
Kidney	ENSAMEG00000010682	RERGL	RERG like
Kidney	ENSAMEG00000010732	ARSG	arylsulfatase G
Kidney	ENSAMEG00000010737	KCNMB2	potassium calcium-activated channel subfamily M regulatory beta subunit 2
Kidney	ENSAMEG00000010744	C11orf86	chromosome 11 open reading frame 86
Kidney	ENSAMEG00000010825	SPTBN2	spectrin beta, non-erythrocytic 2
Kidney	ENSAMEG00000010847	JAKMIP3	Janus kinase and microtubule interacting protein 3
Kidney	ENSAMEG00000010945	FGF9	fibroblast growth factor 9
Kidney	ENSAMEG00000011056	CRB2	crumbs 2, cell polarity complex component
Kidney	ENSAMEG00000011064	OCA2	OCA2 melanosomal transmembrane protein
Kidney	ENSAMEG00000011075	AVP	arginine vasopressin
Kidney	ENSAMEG00000011090	SLC4A9	solute carrier family 4 member 9
Kidney	ENSAMEG00000011139	TRIM50	tripartite motif containing 50
Kidney	ENSAMEG00000011167	DEGS2	delta 4-desaturase, sphingolipid 2
Kidney	ENSAMEG00000011200	PSD2	pleckstrin and Sec7 domain containing 2
Kidney	ENSAMEG00000011284	DEFB129	defensin beta 129
Kidney	ENSAMEG00000011508	CCK	cholecystokinin
Kidney	ENSAMEG00000011529	CLDN16	claudin 16
Kidney	ENSAMEG00000011558	C3orf18	chromosome 3 open reading frame 18
Kidney	ENSAMEG00000011588	ERICH4	glutamate rich 4
Kidney	ENSAMEG00000011729	FAM131C	family with sequence similarity 131 member C
Kidney	ENSAMEG00000011751	WDR72	WD repeat domain 72
Kidney	ENSAMEG00000011842	APOH	apolipoprotein H

Kidney	ENSAMEG00000011859	ARFGEF3	ARFGEF family member 3
Kidney	ENSAMEG00000011870	SLC38A3	solute carrier family 38 member 3
Kidney	ENSAMEG00000011881	AGMAT	agmatinase
Kidney	ENSAMEG00000011913	RIMS3	regulating synaptic membrane exocytosis 3
Kidney	ENSAMEG00000012066	ACOX2	acyl-CoA oxidase 2
Kidney	ENSAMEG00000012070	SYT2	synaptotagmin 2
Kidney	ENSAMEG00000012077	CLCN5	chloride voltage-gated channel 5
Kidney	ENSAMEG00000012147	LAD1	ladinin 1
Kidney	ENSAMEG00000012216	LOC100468789	sulfotransferase 1 family member D1
Kidney	ENSAMEG00000012249	SLC6A20	solute carrier family 6 member 20
Kidney	ENSAMEG00000012393	MST1	macrophage stimulating 1
Kidney	ENSAMEG00000012498	TLDC2	TBC/LysM-associated domain containing 2
Kidney	ENSAMEG00000012504	WFDC3	WAP four-disulfide core domain 3
Kidney	ENSAMEG00000012586	MYL3	myosin light chain 3
Kidney	ENSAMEG00000012602	PTH1R	parathyroid hormone 1 receptor
Kidney	ENSAMEG00000012664	LZTS1	leucine zipper tumor suppressor 1
Kidney	ENSAMEG00000012709	--	--
Kidney	ENSAMEG00000012726	DEFB1	beta-defensin 103
Kidney	ENSAMEG00000012834	KIF12	kinesin family member 12
Kidney	ENSAMEG00000012842	LOC100470802	chondroitin sulfate proteoglycan 4
Kidney	ENSAMEG00000012851	AMBP	alpha-1-microglobulin/bikunin precursor
Kidney	ENSAMEG00000012934	C19orf81	chromosome 19 open reading frame 81
Kidney	ENSAMEG00000012953	--	--
Kidney	ENSAMEG00000012956	SLC44A4	solute carrier family 44 member 4
Kidney	ENSAMEG00000013008	SHISA3	shisa family member 3
Kidney	ENSAMEG00000013063	RHCG	Rh family C glycoprotein
Kidney	ENSAMEG00000013086	APOB	apolipoprotein B
Kidney	ENSAMEG00000013120	ICA1L	islet cell autoantigen 1 like
Kidney	ENSAMEG00000013125	HNF1B	HNF1 homeobox B
Kidney	ENSAMEG00000013160	IL1RL1	interleukin 1 receptor like 1
Kidney	ENSAMEG00000013169	CYP24A1	cytochrome P450 family 24 subfamily A member 1
Kidney	ENSAMEG00000013201	SLC9A4	solute carrier family 9 member A4
Kidney	ENSAMEG00000013210	SLC9A2	solute carrier family 9 member A2
Kidney	ENSAMEG00000013219	PTPRQ	protein tyrosine phosphatase, receptor type Q
Kidney	ENSAMEG00000013223	BCAS1	breast carcinoma amplified sequence 1
Kidney	ENSAMEG00000013224	HS3ST5	heparan sulfate-glucosamine 3-sulfotransferase 5
Kidney	ENSAMEG00000013243	NAPSA	napsin A aspartic peptidase
Kidney	ENSAMEG00000013253	POU3F3	POU class 3 homeobox 3
Kidney	ENSAMEG00000013267	--	--
Kidney	ENSAMEG00000013302	MSI1	musashi RNA binding protein 1
Kidney	ENSAMEG00000013326	C8G	complement C8 gamma chain
Kidney	ENSAMEG00000013342	BICC1	BicC family RNA binding protein 1
Kidney	ENSAMEG00000013473	CRYGN	crystallin gamma N

Kidney	ENSAMEG00000013482	--	--
Kidney	ENSAMEG00000013557	SLC26A4	solute carrier family 26 member 4
Kidney	ENSAMEG00000013572	P2RX2	purinergic receptor P2X 2
Kidney	ENSAMEG00000013675	GATA6	GATA binding protein 6
Kidney	ENSAMEG00000013706	VDR	vitamin D receptor
Kidney	ENSAMEG00000013714	--	--
Kidney	ENSAMEG00000013732	KCNK5	potassium two pore domain channel subfamily K member 5
Kidney	ENSAMEG00000013806	LOC105241896	receptor-type tyrosine-protein phosphatase delta
Kidney	ENSAMEG00000013814	TSPAN1	tetraspanin 1
Kidney	ENSAMEG00000013822	RBP4	retinol binding protein 4
Kidney	ENSAMEG00000013996	--	--
Kidney	ENSAMEG00000014004	PNLIPRP2	pancreatic lipase related protein 2 (gene/pseudogene)
Kidney	ENSAMEG00000014028	RGS7	regulator of G protein signaling 7
Kidney	ENSAMEG00000014044	SLC28A1	solute carrier family 28 member 1
Kidney	ENSAMEG00000014148	EMX2	empty spiracles homeobox 2
Kidney	ENSAMEG00000014170	RBFOX3	RNA binding fox-1 homolog 3
Kidney	ENSAMEG00000014182	PAQR5	progestin and adipoQ receptor family member 5
Kidney	ENSAMEG00000014194	SLC15A2	solute carrier family 15 member 2
Kidney	ENSAMEG00000014244	SCRG1	stimulator of chondrogenesis 1
Kidney	ENSAMEG00000014275	RAP1GAP	RAP1 GTPase activating protein
Kidney	ENSAMEG00000014276	CASR	calcium sensing receptor
Kidney	ENSAMEG00000014315	--	--
Kidney	ENSAMEG00000014423	KLK15	kallikrein related peptidase 15
Kidney	ENSAMEG00000014450	KLK1	kallikrein 1
Kidney	ENSAMEG00000014562	RHBG	Rh family B glycoprotein (gene/pseudogene)
Kidney	ENSAMEG00000014573	UGT8	UDP glycosyltransferase 8
Kidney	ENSAMEG00000014585	ARID3C	AT-rich interaction domain 3C
Kidney	ENSAMEG00000014597	PLEKHG6	pleckstrin homology and RhoGEF domain containing G6
Kidney	ENSAMEG00000014601	ATP12A	ATPase H+/K+ transporting non-gastric alpha2 subunit
Kidney	ENSAMEG00000014698	HSD17B14	hydroxysteroid 17-beta dehydrogenase 14
Kidney	ENSAMEG00000014701	NPHS2	NPHS2, podocin
Kidney	ENSAMEG00000014721	LOC100473915	insulin receptor-related protein-like
Kidney	ENSAMEG00000014736	TDRD5	tudor domain containing 5
Kidney	ENSAMEG00000014737	NTRK1	neurotrophic receptor tyrosine kinase 1
Kidney	ENSAMEG00000014765	SIM1	single-minded family bHLH transcription factor 1
Kidney	ENSAMEG00000014828	TFR2	transferrin receptor 2
Kidney	ENSAMEG00000014901	CRABP2	cellular retinoic acid binding protein 2
Kidney	ENSAMEG00000014904	CCL16	C-C motif chemokine ligand 16
Kidney	ENSAMEG00000014944	CALB1	calbindin 1
Kidney	ENSAMEG00000014957	EGF	epidermal growth factor
Kidney	ENSAMEG00000015034	--	--
Kidney	ENSAMEG00000015045	SLC22A2	solute carrier family 22 member 2

Kidney	ENSAMEG00000015079	STAG3	stromal antigen 3
Kidney	ENSAMEG00000015089	XPNPEP2	X-prolyl aminopeptidase 2
Kidney	ENSAMEG00000015162	WEE2	Wee1-like protein kinase 2
Kidney	ENSAMEG00000015200	--	--
Kidney	ENSAMEG00000015230	C6	complement C6
Kidney	ENSAMEG00000015302	NRAP	nebulin related anchoring protein
Kidney	ENSAMEG00000015305	LOC100473415	creatine kinase U-type, mitochondrial
Kidney	ENSAMEG00000015349	HABP2	hyaluronan binding protein 2
Kidney	ENSAMEG00000015459	SLC7A9	solute carrier family 7 member 9
Kidney	ENSAMEG00000015503	NKAIN3	sodium/potassium transporting ATPase interacting 3
Kidney	ENSAMEG00000015623	EMX1	empty spiracles homeobox 1
Kidney	ENSAMEG00000015644	SERPINC1	serpin family C member 1
Kidney	ENSAMEG00000015680	CRP	C-reactive protein
Kidney	ENSAMEG00000015838	CKMT2	creatine kinase, mitochondrial 2
Kidney	ENSAMEG00000015860	ANKRD53	ankyrin repeat domain 53
Kidney	ENSAMEG00000015864	ATP6V1B1	ATPase H+ transporting V1 subunit B1
Kidney	ENSAMEG00000016034	--	--
Kidney	ENSAMEG00000016164	UGT1A1	UDP glucuronosyltransferase family 1 member A1
Kidney	ENSAMEG00000016218	SLC5A2	solute carrier family 5 member 2
Kidney	ENSAMEG00000016276	AIF1L	allograft inflammatory factor 1 like
Kidney	ENSAMEG00000016281	LAMC3	laminin subunit gamma 3
Kidney	ENSAMEG00000016318	WNT9A	Wnt family member 9A
Kidney	ENSAMEG00000016382	ASGR1	asialoglycoprotein receptor 1
Kidney	ENSAMEG00000016389	SLC34A3	solute carrier family 34 member 3
Kidney	ENSAMEG00000016399	SLC5A8	solute carrier family 5 member 8
Kidney	ENSAMEG00000016427	SLC10A2	solute carrier family 10 member 2
Kidney	ENSAMEG00000016432	CCKBR	cholecystokinin B receptor
Kidney	ENSAMEG00000016453	DAO	D-amino acid oxidase
Kidney	ENSAMEG00000016507	PCK2	phosphoenolpyruvate carboxykinase 2, mitochondrial
Kidney	ENSAMEG00000016517	NCAM1	neural cell adhesion molecule 1
Kidney	ENSAMEG00000016643	LMX1B	LIM homeobox transcription factor 1 beta
Kidney	ENSAMEG00000016731	KIF1A	kinesin family member 1A
Kidney	ENSAMEG00000016743	SLC12A3	solute carrier family 12 member 3
Kidney	ENSAMEG00000016756	CPLX1	complexin 1
Kidney	ENSAMEG00000016843	--	--
Kidney	ENSAMEG00000016861	LOC100470789	receptor-type tyrosine-protein phosphatase delta
Kidney	ENSAMEG00000016879	INHA	inhibin alpha subunit
Kidney	ENSAMEG00000016904	MSS51	MSS51 mitochondrial translational activator
Kidney	ENSAMEG00000016938	FXYD4	FXYD domain containing ion transport regulator 4
Kidney	ENSAMEG00000017078	SLC29A2	solute carrier family 29 member 2
Kidney	ENSAMEG00000017080	DAB2	DAB2, clathrin adaptor protein
Kidney	ENSAMEG00000017081	KLHDC7A	kelch domain containing 7A
Kidney	ENSAMEG00000017136	CDH16	cadherin 16

Kidney	ENSAMEG00000017191	PAK6	p21 (RAC1) activated kinase 6
Kidney	ENSAMEG00000017262	NDRG4	NDRG family member 4
Kidney	ENSAMEG00000017280	MCOLN3	mucolipin 3
Kidney	ENSAMEG00000017343	OVOL1	ovo like transcriptional repressor 1
Kidney	ENSAMEG00000017389	HSD11B2	hydroxysteroid 11-beta dehydrogenase 2
Kidney	ENSAMEG00000017426	TINAG	tubulointerstitial nephritis antigen
Kidney	ENSAMEG00000017498	PDZK1	PDZ domain containing 1
Kidney	ENSAMEG00000017523	PAX2	paired box 2
Kidney	ENSAMEG00000017529	F13B	coagulation factor XIII B chain
Kidney	ENSAMEG00000017648	KCNIP2	potassium voltage-gated channel interacting protein 2
Kidney	ENSAMEG00000017736	SLC22A13	solute carrier family 22 member 13
Kidney	ENSAMEG00000017765	PSD	pleckstrin and Sec7 domain containing
Kidney	ENSAMEG00000017795	HEPACAM2	HEPACAM family member 2
Kidney	ENSAMEG00000017802	SPOCD1	SPOC domain containing 1
Kidney	ENSAMEG00000017846	NODAL	nodal growth differentiation factor
Kidney	ENSAMEG00000017878	CNNM2	cyclin and CBS domain divalent metal cation transport mediator 2
Kidney	ENSAMEG00000017883	SLC22A12	solute carrier family 22 member 12
Kidney	ENSAMEG00000017952	TMEM213	transmembrane protein 213
Kidney	ENSAMEG00000017954	ATP6V0A4	ATPase H+ transporting V0 subunit a4
Kidney	ENSAMEG00000017960	NEURL1	neuralized E3 ubiquitin protein ligase 1
Kidney	ENSAMEG00000018075	PBLD	phenazine biosynthesis like protein domain containing
Kidney	ENSAMEG00000018105	SLC34A2	solute carrier family 34 member 2
Kidney	ENSAMEG00000018145	SLC34A1	solute carrier family 34 member 1
Kidney	ENSAMEG00000018174	CYBA	cytochrome b-245 alpha chain
Kidney	ENSAMEG00000018206	AMN	amnion associated transmembrane protein
Kidney	ENSAMEG00000018209	PAPLN	papilin, proteoglycan like sulfated glycoprotein
Kidney	ENSAMEG00000018323	VSTM2B	V-set and transmembrane domain containing 2B
Kidney	ENSAMEG00000018346	SLC4A1	solute carrier family 4 member 1 (Diego blood group)
Kidney	ENSAMEG00000018387	DOK6	docking protein 6
Kidney	ENSAMEG00000018459	FGB	fibrinogen beta chain
Kidney	ENSAMEG00000018460	AVPR2	arginine vasopressin receptor 2
Kidney	ENSAMEG00000018551	BEX3	brain expressed X-linked 3
Kidney	ENSAMEG00000018554	TCEAL9	transcription elongation factor A like 9
Kidney	ENSAMEG00000018642	CLDN2	claudin 2
Kidney	ENSAMEG00000018725	KCNE1	potassium voltage-gated channel subfamily E regulatory subunit 1
Kidney	ENSAMEG00000018745	PPP1R3C	protein phosphatase 1 regulatory subunit 3C
Kidney	ENSAMEG00000018819	C1orf210	chromosome 1 open reading frame 210
Kidney	ENSAMEG00000018938	--	--
Kidney	ENSAMEG00000019112	--	--
Kidney	ENSAMEG00000019148	KCNJ1	potassium voltage-gated channel subfamily J member 1
Kidney	ENSAMEG00000019203	KCNJ10	potassium voltage-gated channel subfamily J member 10

Kidney	ENSAMEG00000019286	NAT8	N-acetyltransferase 8 (putative)
Kidney	ENSAMEG00000019456	SSTR2	somatostatin receptor 2
Kidney	ENSAMEG00000019474	CALML3	calmodulin like 3
Kidney	ENSAMEG00000019502	KCNJ16	potassium voltage-gated channel subfamily J member 16
Kidney	ENSAMEG00000019520	C21orf62	chromosome 21 open reading frame 62
Kidney	ENSAMEG00000019524	RNF183	ring finger protein 183
Kidney	ENSAMEG00000019546	GJB1	gap junction protein beta 1
Kidney	ENSAMEG00000019800	NPY2R	neuropeptide Y receptor Y2
Kidney	ENSAMEG00000019880	LRRN2	leucine rich repeat neuronal 2
Kidney	ENSAMEG00000019892	THRSP	thyroid hormone responsive
Kidney	ENSAMEG00000019949	CLDN8	claudin 8
Kidney	Novel00005	--	--
Kidney	Novel00025	--	hypothetical protein EGM_19048, partial
Kidney	Novel00059	--	--
Kidney	Novel00061	--	--
Kidney	Novel00108	--	--
Kidney	Novel00111	--	--
Kidney	Novel00182	--	--
Kidney	Novel00235	--	--
Kidney	Novel00292	--	PREDICTED: small lysine-rich protein 1
Kidney	Novel00315	--	--
Kidney	Novel00316	--	PREDICTED: uncharacterized protein LOC105740805
Kidney	Novel00348	--	--
Kidney	Novel00349	--	--
Kidney	Novel00355	--	--
Kidney	Novel00412	--	PREDICTED: paired box protein Pax-2
Kidney	Novel00417	--	PREDICTED: Kv channel-interacting protein 2 isoform X7
Kidney	Novel00420	--	--
Kidney	Novel00435	--	--
Kidney	Novel00438	--	PREDICTED: proline-rich protein 25
Kidney	Novel00471	--	--
Kidney	Novel00547	--	--
Kidney	Novel00549	--	--
Kidney	Novel00596	--	--
Kidney	Novel00615	--	--
Kidney	Novel00629	--	--
Kidney	Novel00634	--	--
Kidney	Novel00639	--	PREDICTED: V-type proton ATPase subunit G 3
Kidney	Novel00651	--	--
Kidney	Novel00656	--	--
Kidney	Novel00660	--	PREDICTED: LOW QUALITY PROTEIN: papilin
Kidney	Novel00708	--	--
Kidney	Novel00709	--	--

Kidney	Novel00743	--	PREDICTED: homeobox protein EMX1
Kidney	Novel00832	--	tetratricopeptide repeat protein 39B-like protein, partial
Kidney	Novel00849	--	--
Kidney	Novel00877	--	PREDICTED: neuronal calcium sensor 1
Kidney	Novel00900	--	--
Kidney	Novel00915	--	--
Kidney	Novel00923	--	--
Kidney	Novel00925	--	--
Kidney	Novel00938	--	--
Kidney	Novel00988	--	--
Kidney	Novel01021	--	--
Kidney	Novel01039	--	--
Kidney	Novel01084	--	--
Kidney	Novel01118	--	--
Kidney	Novel01171	--	--
Kidney	Novel01210	--	PREDICTED: low-density lipoprotein receptor-related protein 2
Kidney	Novel01211	--	--
Kidney	Novel01229	--	PREDICTED: 60S ribosomal protein L19-like
Kidney	Novel01239	--	--
Kidney	Novel01240	--	--
Kidney	Novel01243	--	--
Kidney	Novel01247	--	--
Kidney	Novel01255	--	PREDICTED: solute carrier family 22 member 11
Kidney	Novel01265	--	PREDICTED: tenascin-N-like
Kidney	Novel01276	--	PREDICTED: homeobox protein Hox-D4a-like
Kidney	Novel01335	--	PREDICTED: uncharacterized protein LOC105236658
Kidney	Novel01339	--	PREDICTED: uncharacterized protein LOC103789652
Kidney	Novel01399	--	--
Kidney	Novel01450	--	--
Kidney	Novel01458	--	hypothetical protein EGM_19048, partial
Kidney	Novel01477	--	--
Kidney	Novel01478	--	--
Kidney	Novel01515	--	PREDICTED: EF-hand and coiled-coil domain-containing protein 1
Kidney	Novel01519	--	--
Kidney	Novel01578	--	PREDICTED: LOW QUALITY PROTEIN: uncharacterized protein LOC101564817
Kidney	Novel01677	--	hypothetical protein PANDA_006701
Kidney	Novel01680	--	PREDICTED: uncharacterized protein LOC104867008
Kidney	Novel01703	--	PREDICTED: cAMP-specific 3',5'-cyclic phosphodiesterase 4D-like
Kidney	Novel01712	--	PREDICTED: killer cell lectin-like receptor 2

Kidney	Novel01793	--	--
Kidney	Novel01801	--	--
Kidney	Novel01872	--	--
Kidney	Novel01875	--	--
Kidney	Novel01884	--	--
Kidney	Novel01887	--	--
Kidney	Novel01913	--	--
Kidney	Novel01928	--	--
Kidney	Novel02073	--	--
Kidney	Novel02134	--	PREDICTED: LOW QUALITY PROTEIN: thiamine transporter 2-like
Kidney	Novel02140	--	--
Kidney	Novel02174	--	--
Kidney	Novel02186	--	--
Kidney	Novel02190	--	PREDICTED: uncharacterized protein LOC102148341
Kidney	Novel02218	--	--
Kidney	Novel02224	--	hypothetical protein BRAFLDRAFT_124569
Kidney	Novel02276	--	--
Kidney	Novel02312	--	--
Kidney	Novel02329	--	hypothetical protein PANDA_009295, partial
Kidney	Novel02362	--	--
Kidney	Novel02365	--	--
Kidney	Novel02373	--	--
Kidney	Novel02374	--	--
Kidney	Novel02381	--	--
Kidney	Novel02396	--	--
Kidney	Novel02419	--	--
Kidney	Novel02420	--	PREDICTED: uncharacterized protein LOC105938209
Kidney	Novel02446	--	PREDICTED: glutathione reductase, mitochondrial
Kidney	Novel02510	--	--
Kidney	Novel02531	--	PREDICTED: killer cell lectin-like receptor 2
Kidney	Novel02606	--	--
Kidney	Novel02617	--	PREDICTED: cystin-1
Kidney	Novel02657	--	putative p150
Kidney	Novel02727	--	--
Kidney	Novel02733	--	Retrovirus-related Pol polyprotein LINE-1
Kidney	Novel02855	--	--
Kidney	Novel02912	--	--
Kidney	Novel03014	--	--
Kidney	Novel03041	--	--
Kidney	Novel03059	--	--
Kidney	Novel03146	--	PREDICTED: uncharacterized protein LOC103092605
Kidney	Novel03150	--	PREDICTED: uncharacterized protein LOC103693446

Kidney	Novel03168	--	--
Kidney	Novel03175	--	--
Kidney	Novel03176	--	--
Kidney	Novel03184	--	--
Kidney	Novel03187	--	--
Kidney	Novel03188	--	PREDICTED: plasmolipin
Kidney	Novel03215	--	--
Kidney	Novel03217	--	--
Kidney	Novel03235	--	--
Kidney	Novel03278	--	--
Kidney	Novel03313	--	--
Kidney	Novel03408	--	hypothetical protein
Kidney	Novel03520	--	--
Kidney	Novel03572	--	--
Kidney	Novel03587	--	--
Kidney	Novel03588	--	--
Kidney	Novel03598	--	--
Kidney	Novel03621	--	PREDICTED: uncharacterized protein LOC105498491 isoform X2
Kidney	Novel03622	--	--
Kidney	Novel03665	--	--
Kidney	Novel03696	--	--
Kidney	Novel03730	--	--
Kidney	Novel03739	--	PREDICTED: C2 calcium-dependent domain-containing protein 4B
Kidney	Novel03742	--	--
Kidney	Novel03748	--	60S ribosomal protein L32-like protein
Kidney	Novel03824	--	--
Kidney	Novel03825	--	--
Kidney	Novel03842	--	--
Kidney	Novel03873	--	--
Kidney	Novel03881	--	--
Kidney	Novel03885	--	--
Kidney	Novel03895	--	--
Kidney	Novel03918	--	--
Kidney	Novel03926	--	--
Kidney	Novel04030	--	unnamed protein product
Kidney	Novel04050	--	PREDICTED: CMP-N-acetylneuraminate-beta-galactosamide-alpha-2,3-sialyltransferase 4 isoform X1
Kidney	Novel04114	--	--
Kidney	Novel04142	--	--
Kidney	Novel04149	--	--

Kidney	Novel04155	--	--
Kidney	Novel04156	--	--
Kidney	Novel04225	--	--
Kidney	Novel04242	--	--
Kidney	Novel04248	--	--
Kidney	Novel04274	--	PREDICTED: folate receptor alpha-like
Kidney	Novel04302	--	PREDICTED: LOW QUALITY PROTEIN: homeobox protein HMX3, partial
Kidney	Novel04328	--	--
Kidney	Novel04348	--	Retrovirus-related Pol polyprotein LINE-1
Kidney	Novel04404	--	--
Kidney	Novel04423	--	PREDICTED: zinc finger X-linked protein ZXDA/ZXDB-like
Kidney	Novel04434	--	hypothetical protein M91_01570
Kidney	Novel04437	--	--
Kidney	Novel04474	--	hypothetical protein M91_16983, partial
Kidney	Novel04476	--	hypothetical protein M91_11778
Kidney	Novel04543	--	--
Kidney	Novel04556	--	PREDICTED: formin-like protein 5
Kidney	Novel04562	--	PREDICTED: leucine-rich repeat-containing protein C10orf11 homolog isoform X4
Kidney	Novel04568	--	--
Kidney	Novel04597	--	--
Kidney	Novel04623	--	--
Kidney	Novel04629	--	--
Kidney	Novel04679	--	--
Kidney	Novel04705	--	unnamed protein product
Kidney	Novel04732	--	hypothetical protein M91_17934
Kidney	Novel04735	--	--
Kidney	Novel04775	--	--
Kidney	Novel04780	--	--
Kidney	Novel04785	--	PREDICTED: surfactant-associated protein 2
Kidney	Novel04894	--	--
Kidney	Novel04909	--	PREDICTED: sodium/myo-inositol cotransporter 2 isoform X2
Kidney	Novel04972	--	PREDICTED: pro-epidermal growth factor-like
Kidney	Novel04978	--	--
Kidney	Novel05007	--	PREDICTED: uncharacterized protein LOC105067830 isoform X2
Kidney	Novel05015	--	--
Kidney	Novel05054	--	--
Kidney	Novel05111	--	--
Kidney	Novel05118	--	PREDICTED: kallikrein-1
Kidney	Novel05392	--	unnamed protein product

Kidney	Novel05393	--	--
Kidney	Novel05444	--	PREDICTED: sphingosine-1-phosphate phosphatase 2-like
Kidney	Novel05490	--	--
Kidney	Novel05498	--	PREDICTED: brain and acute leukemia cytoplasmic protein, partial
Kidney	Novel05528	--	--
Kidney	Novel05546	--	--
Kidney	Novel05562	--	PREDICTED: cytosolic beta-glucosidase
Kidney	Novel05578	--	--
Kidney	Novel05704	--	PREDICTED: cytochrome P450 4A6-like
Kidney	Novel05725	--	PREDICTED: LOW QUALITY PROTEIN: sodium/myo-inositol cotransporter 2-like, partial
Kidney	Novel05915	--	hypothetical protein PANDA_022434, partial
Kidney	Novel05948	--	PREDICTED: solute carrier family 22 member 11-like
Kidney	Novel05956	--	PREDICTED: multidrug and toxin extrusion protein 2-like
Kidney	Novel05996	--	PREDICTED: coiled-coil domain-containing protein 151
