# Supplementary material for: Transcriptome Profiling across Five Tissues of Giant Panda
Source: Biomed Res Int. 2020 Aug 10;2020:3852586. doi: 10.1155/2020/3852586 (PMC7436357; doi:10.1155/2020/3852586)
Supplement: Supplementary Materials — is available for this paper at https://new.hindawi.com/journals/bmri/. Supplementary Table S1: individual information of the giant panda used in this study. Supplementary Table S2: quality summary of RNA-seq results in all samples of the giant panda. Supplementary Table S3: details of all genes mapped to the giant panda reference genome in the present study. Supplementary Table S4: list of tissue-specific differentially expressed genes of each tissue of giant panda. Supplementary Table S5: list of significantly enriched GO terms of tissue-specific differentially expressed genes of each tissue. Supplementary Table S6: list of significantly enriched KEGG pathways of tissue-specific differentially expressed genes of each tissue. Supplementary Figure S1: four-way Venn diagram of tissue-specific differentially expressed 466 genes of each tissue. [file 3852586.f1.zip › Supplementary Table S2 Quality summar.pdf]

Supplementary Information

BioMed Research International

Transcriptome profiling across five tissues of giant panda

Feng Li[1,2,4], Chengdong Wang[3,4], Zhongxian Xu[1,4], Mingzhou Li[1], Linhua Deng[3], Ming Wei[3], Hemin Zhang[3], Kai Wu[3], Ruihong Ning[1], Diyan Li[1], Mingyao Yang[1], Mingwang Zhang[1], Qingyong Ni[1], Bo Zeng[1*], Desheng Li[3*] and Ying Li[1*]

[1] Farm Animal Genetic Resources Exploration and Innovation Key Laboratory of Sichuan Province, Sichuan Agricultural University, Chengdu 611130, China.

[2] Key Laboratory of Southwest China Wildlife Resources Conservation (Ministry of Education), China West Normal University, Nanchong 637002, China.

[3] Key Laboratory of SFGA on Conservation Biology of Rare Animals in the Giant Panda National Park (CCRCGP), Dujiangyan 611830, China.

[4] These authors contributed equally to this work.

[*] Correspondence should be addressed to Ying Li, yingli@sicau.edu.cn; Desheng Li, 1050133153@qq.com; Bo Zeng, apollobovey@163.com.

Supplementary Table S2: Quality summary of RNA-seq results in all samples of giant panda.

Sample	Raw reads	Clean reads	Clean bases (Gb)	Q30 (%)	Error rate (%)	Total mapped reads	Uniquely mapped reads
Heart_1	51,744,936	50,726,066	7.61	94.97	0.01	46,634,739	45,648,774
Heart_2	47,851,148	46,089,008	6.91	93.12	0.01	42,228,397	41,273,541
Heart_3	50,628,204	48,708,950	7.31	94.05	0.01	45,779,024	44,894,566
Heart_4	51,844,648	50,187,242	7.53	93.82	0.01	46,795,885	45,836,127
Heart_5	53,270,276	51,646,352	7.75	93.77	0.01	48,673,961	47,849,884
Liver_1	50,465,764	49,475,252	7.42	94.98	0.01	45,920,054	44,917,153
Liver_2	59,693,254	57,194,518	8.58	93.24	0.01	52,588,523	51,344,776
Liver_3	49,170,054	47,299,158	7.09	94.16	0.01	44,400,247	42,384,130
Liver_4	49,991,182	48,422,536	7.26	94.00	0.01	45,171,770	44,249,994
Liver_5	57,827,022	57,302,930	8.60	94.61	0.01	54,315,952	53,057,011
Spleen_1	50,401,038	49,133,374	7.37	94.9	0.01	44,017,687	43,244,089
Spleen_2	50,067,098	47,923,264	7.19	91.83	0.02	41,770,566	41,081,521
Spleen_3	50,460,884	48,556,290	7.28	93.50	0.01	44,372,147	43,580,484
Spleen_4	57,571,712	55,730,934	8.36	93.64	0.01	50,687,962	49,857,154
Spleen_5	62,963,770	60,993,434	9.15	94.14	0.01	55,556,977	54,448,341
Lung_1	54,406,030	53,098,304	7.96	94.84	0.01	47,689,472	46,882,040
Lung_2	55,993,872	53,194,246	7.98	92.76	0.02	47,664,214	47,089,945
Lung_3	53,314,866	51,242,152	7.69	93.75	0.01	47,139,964	46,451,691
Lung_4	59,395,156	57,417,972	8.61	93.64	0.01	52,593,123	51,923,926

Lung_5	57,572,592	55,718,040	8.36	93.75	0.01	51,351,491	50,578,055
Kidney_1	46,951,278	45,665,666	6.85	95.03	0.01	41,475,658	40,855,919
Kidney_2	55,466,166	53,273,152	7.99	92.71	0.02	47,529,941	46,551,630
Kidney_3	54,975,436	52,876,546	7.93	93.60	0.01	49,054,976	48,313,814
Kidney_4	52,772,262	50,726,754	7.61	93.86	0.01	46,636,745	45,698,448
Kidney_5	55,971,720	54,208,226	8.13	94.21	0.01	50,480,440	49,726,761
Mean	53,630,815	51,872,415	7.78	93.88	0.01	47,621,197	46,709,591
