# Supplementary figures and images for: Transcriptome Profiling across Five Tissues of Giant Panda
Source: Biomed Res Int. 2020 Aug 10;2020:3852586. doi: 10.1155/2020/3852586 (PMC7436357; doi:10.1155/2020/3852586)

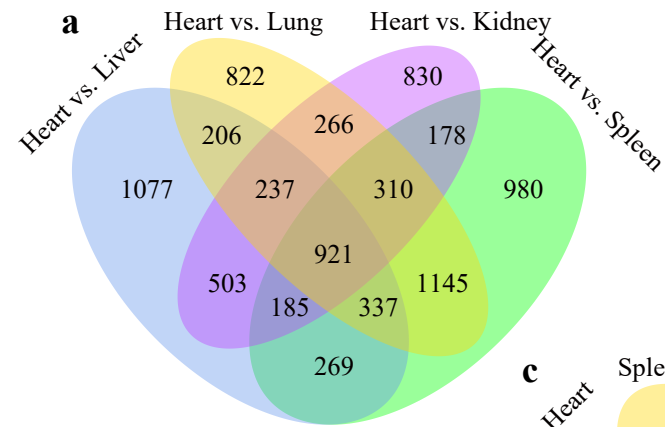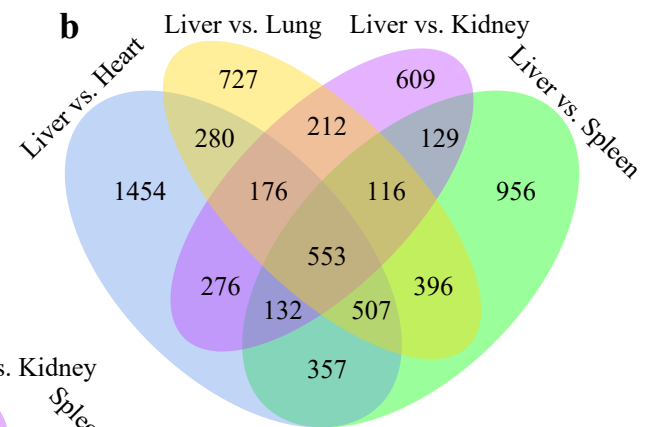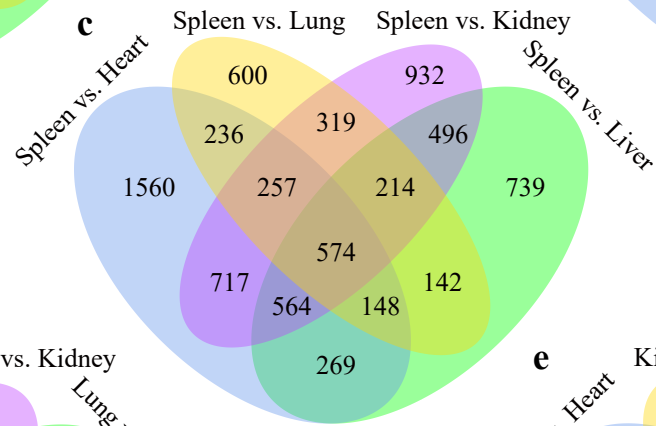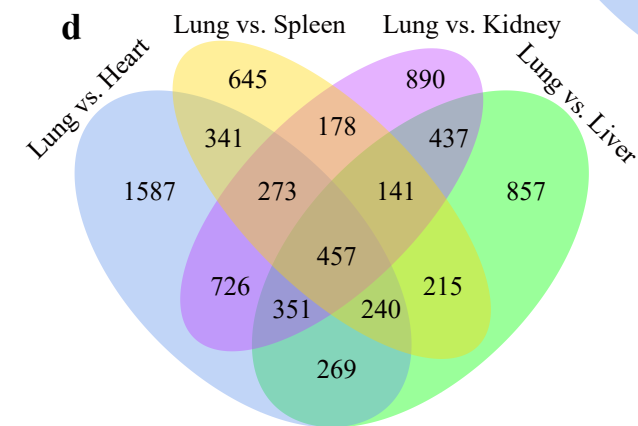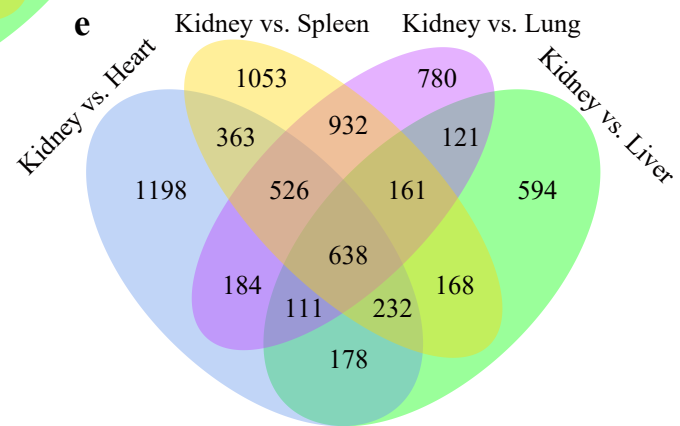

Supplement: Supplementary Materials — is available for this paper at https://new.hindawi.com/journals/bmri/. Supplementary Table S1: individual information of the giant panda used in this study. Supplementary Table S2: quality summary of RNA-seq results in all samples of the giant panda. Supplementary Table S3: details of all genes mapped to the giant panda reference genome in the present study. Supplementary Table S4: list of tissue-specific differentially expressed genes of each tissue of giant panda. Supplementary Table S5: list of significantly enriched GO terms of tissue-specific differentially expressed genes of each tissue. Supplementary Table S6: list of significantly enriched KEGG pathways of tissue-specific differentially expressed genes of each tissue. Supplementary Figure S1: four-way Venn diagram of tissue-specific differentially expressed 466 genes of each tissue. [file 3852586.f1.zip › Supplementary Figure S1 Four-way Venn diagram of tissu.pdf]
